# A systematic scoping review on patients’ perceptions of dignity

**DOI:** 10.1186/s12904-022-01004-4

**Published:** 2022-07-04

**Authors:** Keith Zi Yuan Chua, Elaine Li Ying Quah, Yun Xue Lim, Chloe Keyi Goh, Jieyu Lim, Darius Wei Jun Wan, Simone Meiqi Ong, Chi Sum Chong, Kennan Zhi Guang Yeo, Laura Shih Hui Goh, Ray Meng See, Alexia Sze Inn Lee, Yun Ting Ong, Min Chiam, Eng Koon Ong, Jamie Xuelian Zhou, Crystal Lim, Simon Yew Kuang Ong, Lalit Krishna

**Affiliations:** 1 Yong Loo Lin School of Medicine, National University of Singapore, NUHS Tower Block, 1E Kent Ridge Road, Level 11, Singapore, 119228 Singapore; 2 Division of Supportive and Palliative Care, 11 Hospital Crescent, National Cancer Centre, Singapore, 169610 Singapore; 3 Division of Cancer Education, 11 Hospital Crescent, National Cancer Centre, Singapore, 169610 Singapore; 4 Duke-NUS Medical School, National University of Singapore, 8 College Rd, Singapore, 169857 Singapore; 5 Medical Social Services, Singapore General Hospital, Outram Rd, Singapore, 169608 Singapore; 6 Division of Medical Oncology, National Cancer Centre Singapore, 11 Hospital Crescent, Singapore, 169610 Singapore; 7 Academic Palliative Care Unit, United Kingdom Cancer Research Centre, University of Liverpool, University of Liverpool, 200 London Rd, Liverpool, L3 9TA UK; 8 Centre of Biomedical Ethics, National University of Singapore, 21 Lower Kent Ridge Rd, Singapore, 119077 Singapore; 9 The Palliative Care Centre for Excellence in Research and Education, PalC c/o Dover Park Hospice, 10 Jalan Tan Tock Seng, Singapore, 308436 Singapore

**Keywords:** Dignity, Patients, Review, Medicine

## Abstract

**Background:**

A socioculturally appropriate appreciation of dignity is pivotal to the effective provision of care for dying patients. Yet concepts of dignity remain poorly defined. To address this gap in understanding and enhance dignity conserving end-of-life care, a review of current concepts of dignity is proposed.

**Methods:**

To address its primary research question “How do patients conceive the concept of dignity at the end of life?”, this review appraises regnant concepts and influences of dignity, and evaluates current dignity conserving practices. To enhance accountability, transparency and reproducibility, this review employs the Ring Theory of Personhood (RToP) as its theoretical lens to guide a Systematic Evidence Based Approach guided Systematic Scoping Review (SSR in SEBA) of patient perspectives of dignity. Three independent teams of reviewers independently analysed included articles from a structured search of PubMed, Embase, PsycINFO, Scopus, CINAHL and Cochrane Databases using thematic and content analyses. The themes and categories identified were compared and combined using the Funnelling Process to create domains that guide the discussion that follows.

**Results:**

Seventy-eight thousand five hundred seventy-five abstracts were identified, 645 articles were reviewed, and 127 articles were included. The three domains identified were definitions of dignity, influences upon perceptions of dignity, and dignity conserving care.

**Conclusions:**

This SSR in SEBA affirms the notion that dignity is intimately entwined with self-concepts of personhood and that effective dignity conserving measures at the end of life must be guided by the patient’s concept of dignity. This SSR in SEBA posits that such personalised culturally sensitive, and timely support of patients, their family and loved ones may be possible through the early and longitudinal application of a RToP based tool.

**Supplementary Information:**

The online version contains supplementary material available at 10.1186/s12904-022-01004-4.

## Background

Drawn from the Latin terms dignitus (merit) and dignus (worth) the concept of dignity is seen as the embodiment of an individual’s intrinsic and inalienable right to respect, and a measure of self-worth and honour [1–3]. Yet, the concept of dignity takes a variety of forms in the professional, legal, philosophical and ethics realm. For some it is inextricably tied to the moral, ethical and legal notions of autonomy [4], and individual rights [5] whilst to others dignity is a construct rooted in regnant sociocultural influences and beliefs [6]. In extoling dignity’s evolving, personalized often context dependent nature Chochinov adds a further dimension to current concepts [7]. Indeed, failure to acknowledge dignity as an evolving sociocultural construct shaped by ‘both social and cultural constructs and the interrelationships between them’ that has exposed differences in Eastern and Western concepts of dignity and raised questions as to the efficacy of generic dignity conserving measures in healthcare [6, 8–10].

### Need for this review

With dignity conservation a crucial aspect of end of life care, better understanding of the concept of dignity is crucial to the provision of individualised care for patients, their families, and caregivers [11].

### Theoretical lens

**Fig. 1:**
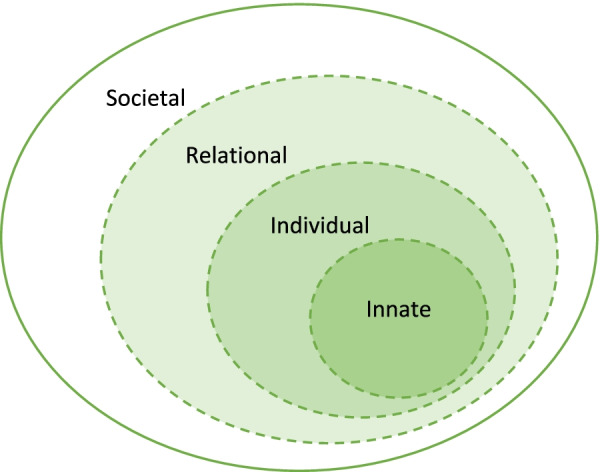
The ring theory of personhood

As a socio-cultural concept influenced by regnant religious beliefs, societal mores, moral and cultural codes, and evolving personal narratives and contextual considerations, the study of current theories of dignity demands a holistic and longitudinal evaluation. Positing that current concepts of dignity are informed by self-concepts of personhood or “what makes you, you”, we adopt Krishna [12] ’s concept of the Ring Theory of Personhood (RToP) to evaluate current ideas on dignity [13–15]. Shown to capture individualised notions of identity, self-worth and respect [16–21] that are intimately associated with current ideas of dignity the RToP provides a robust and evidence-based lens to appraise current this individualised and changing concept (Fig. 1).

The employ of the RToP as a theoretical lens is also based on current characterisation of dignity [22]. Jacobson [23] suggests the existence of human dignity and social dignity. Jacobson [23] posits that human dignity “belongs to every human being simply by virtue of being human” and that it “cannot be created or destroyed”. Social dignity is “generated in the interactions between and amongst individuals, collectives and societies” and confers self-respect and self-worth as well as respect of the individual by the collective and society [24]. Macklin [15] on the other hand suggests that dignity is a function of autonomous action. Ho, Krishna [18], Foo, Zheng [19], Ho, Krishna [20], Chong, Quah [21], Chai, Krishna [14], suggest that dignity and indeed respect for the individual relates to their associations, responsibilities, roles and place within a family unit whilst Ong, Krishna [13], Wei and Krishna [24], Lee, Sim [25], Loh, Tan [26] propose that selfhood, individual dignity, personal rights and respect are tied to wider sociocultural constructs.

Each of these concepts of dignity are captured in the clinically-evidenced RToP’s Innate, Individual, Relational and Societal Rings. Each ring contains specific beliefs, moral values, ethical principles, familial mores, cultural norms, attitudes, thoughts, decisional preferences, roles and responsibilities that create domain-based identities which in turn inform personal concepts of dignity.

Much like Jacobson [23] notion of human dignity, the Innate Ring is anchored in the belief that all humans are deserving of personhood, “irrespective of clinical status, culture, creed, gender, sexual orientation, religion, or appearance” [13–15, 22, 23]. The Innate Ring contains gender, name, family identity, religious and cultural, community and nationality based beliefs, moral values, ethical principles, familial mores, cultural norms, attitudes, thoughts, decisional preferences, roles and responsibilities (henceforth beliefs, values and principles).

Much like Macklin’s [25] notion of dignity being a function of autonomous function, the Individual Ring is informed by the individual’s preferences, biases, beliefs, mores, norms, values and principles which in turn inform personal concepts of dignity. Yet the Individual Ring is also informed by psycho-emotional, experiential, perceptual, and contextual considerations; individual preferences and decision-making styles and biases; and prevailing professional, sociocultural, legal, ethical, and personal considerations. The Individual Ring reveals the evolving and context specific nature of concepts of dignity [27].

The Relational Ring consists of all the relationships that the individual considers close and important to them. As current concepts of dignity acknowledge that concepts of identity, dignity and personhood are shaped by the beliefs, values and principles held by people with whom the individual shares personal and important ties with, the Relational Ring is not exclusively informed by family members and considers the influence of friends with whom the individual determines shares important ties with them [28–30]. The Societal Ring is the outermost ring and encompasses societal, religious, professional and legal expectations and institutional obligations and legal standards of practice. These facets inform the individual’s clinical responsibilities, academic codes of conduct, institutional roles, societal expectations, professional duties, and legal and ethical codes of conduct. It could be said that the Relational and Societal Rings embody Jacobson [23] ’s notion of social dignity.

With concepts of personhood and dignity being personalised and context-dependent, how they are conceived with respect to issues such as withholding and withdrawing treatment [31], care determinations [13], collusion [14], and end-of-life care [32], requires careful consideration. The RToP offers both a reflexive, longitudinal, holistic and evidence-based approach to capture evolving concepts of dignity [12, 32–39]. Using the lens of the RToP it is possible to understand how the Individual Ring and its associated concept of Individual Identity balance sometimes competing preferences, biases, beliefs, mores, norms, values and principles, in a variety of psycho-emotional, experiential, perceptual, and contextual considerations; and prevailing professional, sociocultural, legal, ethical, and personal considerations [16, 17, 40–44].

## Methods

Krishna’s Systematic Evidence-Based Approach (SEBA) is adopted to guide this systematic scoping review (SSR) (henceforth SSR in SEBA) [40, 45–51]. The aim of this review is to identify available data, key characteristics and knowledge gaps in current concepts of dignity in the literature. The SSR in SEBA’s constructivist approach [46, 47, 52–57] and relativist lens [58–62] acknowledges dignity as a sociocultural construct. It also facilitates systematic extraction, synthesis and summary of actionable and applicable information across a diverse range of study formats and overcomes the absence of a common understanding of dignity.

To provide a balanced review, an expert team comprised of a librarian from the National University of Singapore’s (NUS) Yong Loo Lin School of Medicine (YLLSoM) and local educational experts and clinicians at YLLSoM, National Cancer Centre Singapore, Palliative Care Institute Liverpool, and Duke-NUS Medical School (henceforth the expert team) helped to guide the 6 stages of the SEBA process.

**Fig. 2:**
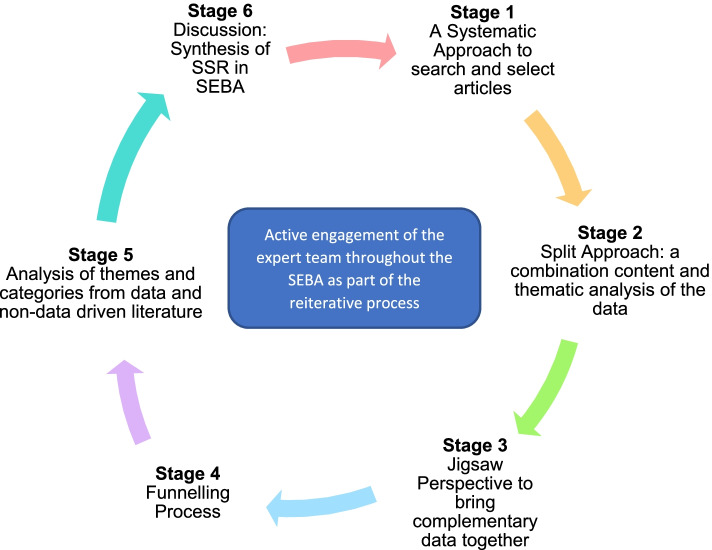
The SEBA Process

The SEBA process consists of the 1) Systematic Approach, 2) Split Approach, 3) Jigsaw Perspective, 4) Funnelling Process 5) Analysis of data and non-data driven literature, and 6) Discussion Synthesis (Fig. 2).

### Stage 1 of SEBA: Systematic approach

Stage 1 of the SEBA methodology involves a systematic search of key databases to answer the primary and secondary research questions established by the research and expert teams.

i. Determining the title and background of the review
  The expert team, stakeholders and the research team determined the goals of the study and confined the study population, context and concept of the systematic scoping review to the perspectives and factors affecting dignity amongst patients. 
ii. Identifying the research question
  Guided by the expert team, the research team determined the primary research question to be: “ How do patients conceive the concept of dignity?” The secondary research questions were: What factors affect patient perceptions of dignity?” and “How are prevailing dignity-conserving care practices perceived by patients?” These questions were designed around the Population, Concept, and Context (PCC) elements of the inclusion criteria [63]. In keeping with the SEBA methodology, the review was guided by the PRISMA-P 2015 (Preferred Reporting Items for Systematic Reviews and Meta-Analyses Protocols) checklist [64]. 
iii. Inclusion criteria
  In keeping with the SEBA methodology, a PICOs (Population, Intervention, Comparison, Outcome, study design) format was adopted to guide the research process (Table 1). Here there is was no comparison group.
iv. Searching
  Seven members of the research team carried out independent searches of five bibliographic databases (PubMed, Embase, PsycINFO, Cochrane Database of Systematic Reviews, CINAHL, Scopus). To facilitate this approach, the search process saw three experienced senior researchers well versed in carrying out systematic reviews and systematic scoping reviews each meet with a team of 2–3 medical students to guide them database searches. This approach was to enhance training of new researchers and to ensure that at least two teams were independently reviewing each database. Each team met regularly and discussed their findings. After a search of the first 100 articles in a particular database, the medical students and the senior researcher compared their findings at an online meeting. Subsequently the teams met at specific time points, often after reviewing a predetermined number of included articles to discuss their concerns, exchange opinions and advance their understanding of the research process and the area of study. Interrater reliability was not evaluated.
  In keeping with Pham, Rajic [65] ’s recommendations on sustaining the research process and accommodating to existing manpower and time constraints, the research team restricted the searches to articles published between 1^st^ January 2000 and 31^st^ December 2020. Quantitative, mixed and qualitative research methodologies meeting the inclusion criteria were included. 
v. Extracting and Charting

**Table 1:** PICOs, Inclusion criteria and exclusion criteria applied to database search

PICOS	Inclusion criteria	Exclusion Criteria
Population	Patients receiving end-of-life care (i.e. palliative care patients) Patients with terminal illnesses or life-limiting conditions	Patients of non-medical specialties such as Veterinary, Dentistry, Alternative and Traditional Medicine Healthcare professionals, defined by and limited to: doctors, nurses, medical social workers Caregivers
Intervention	Provision of dignity-conserving care by healthcare professionals as well as other caregivers including family Seeking to understand patients’ perceptions of their own dignity Seeking to understand factors impacting dignity	Non-dignity focused interventions
Comparison	Various practices in dignity-conserving care in hospital and care settings Factors affecting dignity Comparisons between different forms of dignity-conserving care	N/A
Outcome	Practices of dignity-conserving care Impact of dignity-conserving care practices on patients’ dignity Impact of differences in stakeholders’ perceptions of dignity on patient care	Outcomes not relevant to patient dignity
Study design	Articles in English or translated to English All study designs including: mixed methods research, meta-analyses, systematic reviews, randomized controlled trials, cohort studies, case–control studies, cross-sectional studies, and descriptive papers Years of Publication: between 1^st^ January 2000 and 31^st^ December 2020 Databases: PubMed, Embase, PsycINFO, Cochrane Database of Systematic Reviews, Scopus, CINAHL	Articles in languages other than English Publications before 1^st^ January 2000 or after 31^st^ December 2020

Working in teams of three medical students and a senior reviewer, the teams reviewed the abstracts and titles and discussed their findings at regular meetings. The findings of the three teams were then discussed at online meetings where Sandelowski and Barroso [66] ’s ‘negotiated consensual validation’ was used to achieve consensus on the final list of titles to be reviewed. The three research teams repeated this process, independently studying all the full text articles on the final list of titles, creating their own lists of articles to be included and discussing their findings online at research meetings. Consensus was achieved on the final list of articles to be analysed.

### Stage 2 of SEBA: Split approach

Krishna’s ‘Split Approach’ [65–70] was employed to enhance the reliability of the data analyses. This saw three groups of researchers independently analysing the included articles.

The first team summarised and tabulated the included full-text articles in keeping with recommendations drawn from Wong, Greenhalgh [71] ’s RAMESES publication standards: meta-narrative reviews and Popay, Roberts [58] ’s “Guidance on the conduct of narrative synthesis in systematic reviews”. The tabulated summaries served to ensure that key aspects of included articles were not lost (Supplementary File 1).

Concurrently, the second team analysed the included articles using Braun and Clarke [72] ’s approach to thematic analysis. In phase 1, the research team carried out independent reviews, ‘actively’ reading the included articles to find meaning and patterns in the data. In phase 2, ‘codes’ were constructed from the ‘surface’ meaning and collated into a code book to code and analyse the rest of the articles using an iterative step-by-step process. As new codes emerged, these were associated with previous codes and concepts. In phase 3, the categories were organised into themes that best depict the data. An inductive approach allowed themes to be “defined from the raw data without any predetermined classification” [73]. In phase 4, the themes were refined to best represent the whole data set and discussed. In phase 5, the research team discussed the results of their independent analysis online and at reviewer meetings. ‘Negotiated consensual validation’ was used to determine a final list of themes approach and ensure the final themes.

A third team of researchers employed Hsieh and Shannon [74] ’s approach to directed content analysis [74] to analyse the included articles. Analysis using the directed content analysis approach involved “identifying and operationalizing a priori coding categories”. The first stage saw the research team draw categories from Chochinov [75] ’s “Dignity-Conserving Care – A New Model for Palliative Care” to guide the coding of the articles. Any data not captured by these codes were assigned a new code.

### Stage 3 of SEBA: Jigsaw perspective

In keeping with SEBA’s reiterative process, the themes and categories were reviewed by the expert and research teams. Overlaps between the categories and themes were viewed as pieces of a jigsaw puzzle with the intention of combining overlapping/complementary pieces to create a bigger piece of the puzzle referred to as themes/categories. To create themes/categories the Jigsaw Perspective adopted Phases 4 to 6 of France, Uny [76] ’s adaptation of Noblit, Hare [77] ’s seven phases of meta-ethnography. As per Phase 4, the themes and the categories identified in the Split Approach are grouped together according to their focus. These groupings of categories and themes were then contextualized through the review of the articles from which they were drawn from. Reciprocal translation was used to determine if the themes and categories can be used interchangeably. This allows the themes and categories to be combined to form themes/categories.

### Stage 4 of SEBA: Funnelling process

The Funnelling Process employs Phases 3 to 5. To begin, the themes/categories identified in the Jigsaw Approach are juxtaposed with key messages identified in the tabulated summaries to create domains. The process sees the goals, approaches and assessment themes combined within the categories of patient care and procedural skills, interpersonal communication skills, professionalism, knowledge and enablers and barriers. These domains form the basis for ‘the line of argument’ in Stage 6 of SEBA.

## Results

**Fig. 3:**
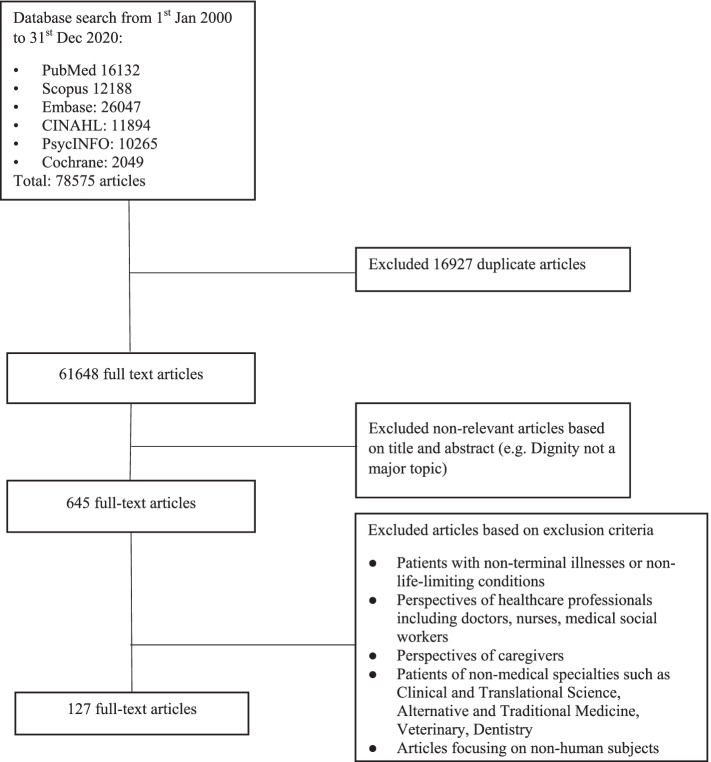
PRISMA Flowchart

78,575 abstracts were identified from the five databases, 645 articles were reviewed, and 127 articles were included (Fig. 3). The three domains identified were: definitions of dignity, factors affecting perceptions of dignity, and dignity-conserving care.

### Domain 1: Definitions of dignity

Forty-six articles proposed patient definitions of dignity. These characterisations and definitions were analysed through lens of the RToP. Subdomains one to four highlight their focus.

### Subdomain 1: The innate ring

Patients believe that being treated “as a person” is an intrinsic and inalienable right of any human being [78–82] by virtue of their ‘spiritual connections’ or as a result of their human appearance [82–86].

### Subdomain 2: The individual ring

Dignity is also characterised by respect of a patient’s individuality [6, 78, 82, 83, 87–93] and independence [78, 80, 83, 84, 86, 87, 89, 91, 93–96]. Respect for independence and individuality is evinced in the treatment of symptoms and efforts to preserve a patient’s ability for self-determination [79, 82, 85, 94, 97–100].

### Subdomain 3: The relational ring

Preservation of familial ties [80, 87, 92, 95] and roles [87] is a key aspect of dignity [84, 87, 90, 92, 95, 101]. Care and support from family members enhanced a patient’s dignity [91–93, 102] whilst being a burden to the family diminished it [87, 89, 91, 93, 95].

### Subdomain 4: The societal ring

The provision of individualised, timely and appropriate communication and support by healthcare professionals (HCP)s was important to maintaining dignity [6, 10, 78, 89, 91, 92, 95, 102].

### Domain 2: Factors affecting patients’ perceptions of dignity

Current influences upon patient’s concepts of dignity may be similarly viewed through the RToP which help focus support.

### Subdomain 1: The innate ring

The patient’s sense of self, body image and spirituality impacts their sense of dignity. Thus age-appropriate care [7, 84, 103–105] that also respects the patient’s physical characteristics [83–85, 106–111], culture [75, 78, 82, 92, 94] and beliefs [95, 112, 113] is essential to maintaining the patient’s self-image [7, 82, 84, 104, 108, 114–118] and well-being [75, 81, 83, 84, 89, 95, 98, 100, 110, 112, 119–128]. Failure to respect this holistic concept replete with physical, cultural, age, gender, spiritual and social narrative [95, 112, 113] may result in a negative body image [7, 82, 84, 104, 108, 114–118], a loss of self [6, 7, 87, 91, 102, 104, 118, 129–135] and a loss of will to live [136].

Sustaining a patient’s holistic concept of self [7, 82, 84, 104, 108, 114–118] is especially pertinent when treating oedema and cachexia, and in the management of surgical scarring, drains or other attached medical equipment [75, 81, 83, 84, 89, 95, 98, 100, 110, 112, 119–128]. Such an approach helps patient’s make sense of their illness and the dying process [81, 95, 98, 120–122, 125], attenuates existential distress [121, 122, 137] and diminishes the effects of a loss of dignity [7, 75, 108, 120, 138, 139].

### Subdomain 2: The individual ring

Dignity is conserved by sustaining their cognitive abilities [7, 83, 90–92, 96, 97, 102–105, 108, 118, 124, 132, 137, 139–143], autonomous function [6, 78, 79, 83, 88, 89, 92, 93, 96–99, 102, 103, 107, 108, 110, 111, 114, 122, 124, 138, 144–151] and independence in personal care [78, 80, 83, 84, 86, 87, 89, 91, 93–96, 105, 108, 111, 113, 116, 124, 127, 128, 139, 140, 143, 147, 150, 152, 153] and activities of daily living [83, 84, 87, 108, 111, 113, 116, 128, 139, 140, 147, 152]. This facilitates a patient’s ability to maintain control over their finances, personal affairs, care determinations including their place of care and death [79, 90, 98, 107, 110, 116, 121, 138, 144, 145, 148, 149, 154], privacy [75, 82, 83, 87, 89, 92, 93, 110, 111, 121, 140, 143, 150], individuality, and legacy are key determinants of self-concepts of dignity [7, 83, 90–92, 96–98, 102–105, 108, 118, 124, 129, 131, 132, 137, 139–143, 155].

Conversely uncertainty [7, 140, 143], changing disease trajectories and prognosis [78, 88, 93, 105, 108, 120, 147, 156, 157], functional deterioration [6, 7, 83, 84, 87, 88, 93, 95–97, 102, 104, 108, 109, 115–118, 128, 129, 132, 140, 147, 158–161] and a loss of control over their financial affairs [95, 111, 121] impairs the patient’s ability to determine their desired place of care and death [116] and predisposes them to a sense of ‘unfinished business’ [7, 98, 118, 131] and an erosion of dignity [6, 78, 82, 83, 87–93, 108, 110, 112, 121, 146–148, 152, 153, 162] and “selfhood” [6, 78, 93, 105, 140, 147]. Poor pain control [87, 90, 111, 127, 147, 152, 163, 164], physical [78, 83, 89, 94, 108, 111, 112, 127, 128, 147, 150–152, 165], and psychoemotional support [75, 78, 79, 83, 87, 89, 94, 108, 110, 111, 114, 120, 147, 150, 152, 162] have similar detrimental effects on the patient’s dignity [6, 7, 82–84, 87, 88, 90, 93, 95–97, 102, 104, 108, 109, 115–118, 128–135, 137, 140, 143, 147, 158–161, 166] and may manifest as fear [75, 79, 83, 108, 147], loneliness [102], emotional lability [112, 118, 129, 167], poor acceptance of their clinical state [88, 98, 105, 120], a loss of hope [78, 108, 147, 156, 157], self-esteem [6, 10, 88, 89, 93, 101, 110, 123] and purpose [78, 87, 105, 108, 118, 127, 129, 130, 148] as well as psychological distress [78, 87, 102, 105, 108, 118, 127, 129, 130, 148, 168, 169].

### Subdomain 3: The relational ring

The Relational Ring is influenced by reliance on family [90, 95, 101, 115, 116, 137, 147], their willingness to support the patient’s needs [90, 95, 101, 115, 116, 137, 147], the patient’s sense of connectedness [91–93, 102, 111, 113, 116, 121, 122, 124] and the quality of their relationships [82, 87, 115, 124, 131, 133, 139]. At the heart of these considerations are patients’ desire to be perceived in a positive light [87, 90, 102, 130, 146, 154] and to maintain their role and status within the family [90, 92, 114, 122, 124, 152]. Feelings of being a burden [87, 89, 91, 93, 95, 108, 154], conflict [121, 124, 125], isolation [111, 113, 124] are especially deleterious to dignity [7, 84, 95, 170]. Table 2 reveals other considerations in the Relational Ring.

**Table 2:** Factors affecting patients’ perceptions of dignity and loss of dignity

Rings	Factors	Loss
Innate	Body Image Physical appearance [82–86, 106–109, 111]	Changes in physical characteristics [7, 82, 84, 104, 108, 114–118] Negative body image [7, 82, 84, 104, 108, 114–118] Ageing [7, 84, 103–105]
	Existential considerations Recognition as a human being [75, 78, 82, 92, 94] Being treated with respect and honor as a human [89, 91, 92, 138, 148, 170, 171]	Existential distress [6, 7, 87, 91, 102, 104, 105, 108, 118, 129–136, 139] Loss of will to live [136] Loss of self [6, 7, 87, 91, 102, 104, 118, 129–135]
	Spirituality Spiritual comfort [84, 95, 100, 110, 119, 120, 122, 124, 127, 128] Beliefs and practices [75, 81, 83, 89, 95, 98, 112, 119–126]	Spiritual distress [7, 75, 108, 120, 138, 139]
Individual	Maintaining control Financial affairs [95, 111, 121] Independence [78, 80, 83, 84, 86, 87, 89, 91, 93–96, 105, 108, 111, 113, 116, 124, 127, 128, 139, 140, 143, 147, 150, 152, 153] Privacy [75, 82, 83, 87, 89, 92, 93, 110, 111, 121, 140, 143, 150] Place of death [90, 110, 116, 121, 154] Autonomy [6, 78, 79, 83, 88, 89, 92, 93, 96–99, 102, 103, 107, 108, 110, 111, 114, 122, 124, 138, 144–151] Maintaining individuality [6, 78, 82, 83, 87–93, 108, 110, 112, 121, 146–148, 152, 153, 162] Legacy [78, 93, 124, 140, 147, 153]	Loss of control over the dying process [7, 83, 90–92, 96–98, 102–105, 108, 118, 124, 129, 131, 132, 137, 139–143, 155] Loss of decision-making capacity [7, 83, 90–92, 96, 97, 102–105, 108, 118, 124, 132, 137, 139, 141–143] Uncertainty [7, 140, 143] Unfinished business [7, 98, 118, 131] Unmet needs [7, 90, 96, 118, 129, 140, 155]
	Symptom distress Physical distress [78, 83, 89, 94, 108, 111, 112, 127, 128, 147, 150–152, 165] Mental distress [75, 78, 79, 83, 87, 89, 94, 108, 110, 111, 114, 120, 147, 150, 152, 162]	Symptomatic distress [6, 7, 82–84, 87, 88, 90, 93, 95–97, 102, 104, 108, 109, 115–118, 128–135, 137, 140, 143, 147, 158–161, 166] Functional deterioration [6, 7, 83, 84, 87, 88, 93, 95–97, 102, 104, 108, 109, 115–118, 128, 129, 132, 140, 147, 158–161] Reliance on others [7, 95, 104, 109, 115, 117, 118, 129, 140, 158, 159]
	Positive emotional state Positive emotions about self [6, 10, 88, 89, 93, 101, 103, 108, 115, 123, 153] Positive emotions about prognosis [78, 88, 93, 105, 108, 120, 147, 156, 157]	Psychological distress [78, 87, 102, 105, 108, 118, 127, 129, 130, 148, 168, 169] Loss of sense of purpose/hope [78, 87, 105, 108, 118, 127, 129, 130, 148] Loss of emotional stability [112, 118, 129, 167] Loneliness [102] Anticipation [169]
Relational	Reliance on family Care and support [84, 87, 90, 92, 95, 101, 106, 108, 110, 113, 115, 116, 121, 122, 128, 144, 172] Aftermath concerns [75, 93, 111, 154]	Lack of care from family [7, 84, 95, 170] Physical care [170] Intangible care [7, 78, 84, 95, 170] Being a burden to family [87, 89, 91, 93, 95, 108, 154]
	Connectedness Engagement [91–93, 102, 111, 113, 116, 121, 122, 124] Conflicts/conflict-resolution with family [121, 124, 125] Depth of relationship [80, 87, 92, 95, 109, 115, 122, 124] Relationship with family [93, 95, 144]	Loss of familial relationships’ quality [82, 87, 115, 124, 131, 133, 139] Feeling of isolation [115] Inability to communicate concerns [124, 139] Conflict [133] Loss of familial roles [87, 131]
	Perception by family How family perceives patient and illness [87, 90, 102, 130, 146, 154] Changing role(s) in family [90, 92, 114, 122, 124, 152]	
Societal	Treatment by healthcare workers [6, 10, 78, 89, 91, 92, 95, 102, 110, 144, 154]	Healthcare system inadequacies [82, 95, 101, 102, 129, 135, 137, 155] Lack of empathy [82, 95, 137] Lack of regard as a person [101, 102, 135] Poor organisation [82, 102, 129]
	Place in society Role preservation [10, 78, 83, 92, 93, 95, 97, 102, 111, 140, 150, 152] Attitudes toward patients by others [75, 79, 84, 87, 90, 97, 102, 124, 143]	Lack of respect/support from society [7, 84, 91, 101, 130, 131] Discrimination/social isolation [90, 92, 96, 101, 170] Loss of role in society [7, 98, 108, 128, 129]
	Reliance on others Social support [75, 78, 82, 83, 89, 91–93, 95, 100, 109, 110, 113, 114, 116, 122, 128, 140]	Dependence on others [95, 114] Feeling burdensome [10, 75, 79, 83, 86, 89, 90, 92, 95, 105, 117, 125, 150, 173]

### Subdomain 4: The societal ring

Societal concepts of dignity feature geographical nuances in current concepts of dignity and reflect the influence of regnant ‘belief systems, experiences, and culture’ on these concepts [6] (Table 3). This especially evident in the differences in the role of relational ties and influences on autonomy in Western and Asian data [6, 10]. Data from China and Japan suggests the influence of relational autonomy, which prioritises familial interests, over individual interests within concepts of personhood and dignity [13, 16, 45].

**Table 3:** Definitions of dignity

Ring	Theme	Country
Innate	Intrinsic worth [78–81, 83, 87, 88, 174] Being acknowledged [175]	USA [174], Canada [78], Spain [79, 83], Iran [175], Netherlands [87], Norway [88], Sweden [80], Germany [81]
	Inalienable right [97, 129, 175]	Iran [175], Netherlands [129], Spain [97]
	Based on rationality, unique to humans [10]	Netherlands [10]
	Being worthy, honoured, or esteemed [75, 78, 89, 98, 119, 120, 158] Being treated with respect, valued by others [84, 90, 101, 103, 138, 176, 177]	Canada [78, 119], China [158], UK [89], USA [98], Italy [75, 120] Sweden [103], Norway [90], France [138], USA [101, 177], Japan [176], Denmark [102], Greece [84]
Individual	Related to physical/ functional symptoms [94, 97]	UK [94], Spain [97]
	Self-construed [6, 10, 87], self-defined [83, 84, 129, 175, 178], personal identity [6, 10, 78, 82–84, 91, 129, 175, 178]	Canada [78], Italy [82], Netherlands [10, 87, 129], Australia [6], Spain [83], Greece [84], Poland [178], Iran [175], UK [91]
	Autonomy [85, 91, 98, 99]	USA [85, 98], Brazil [99], UK [91]
Relational	Caregivers being part of care [170]	Spain [170]
	Maintaining familial ties [80, 87, 92, 95]	Netherlands [87], Singapore [92, 95], Sweden [80]
	Receiving care and support from family [91–93, 102]	Denmark [102], Sweden [93], Singapore [92], UK [91]
	Not wanting to burden family [87, 89, 91, 93, 95]	Netherlands [87], UK [91] [89], Singapore [95], Sweden [93]
	Not wanting to lose familial roles [87]	Netherlands [87]
Societal	Social position [10, 87]	Netherlands [10, 87]
	Rapport with healthcare team [6, 10, 78, 89, 91, 92, 95, 102]	Canada [78], Netherlands [10], UK [91], Denmark [102], Australia [6], Singapore [92, 95]
Multi-ring	Innate and societal worth [78, 93, 95]	Singapore [95], Sweden [93], Canada [78]
	Individual and societal role [78, 92, 101, 103, 112, 158, 179]	Denmark [179], Canada [78], USA [101], Singapore [92], Sweden [103], Italy [112], China [158]
	Innate and individual value [96]	USA [96], Canada [75], Denmark [102]
	Innate, individual, societal place [86, 114, 175, 180]	Netherlands [86], Italy [181], USA [180], Iran [175], Greece [114]
	Individual, relational [130]	China [130]
	Ambiguous [78]	Canada [78]
	Right to how and when to die [100]	Spain [100]
	Death without suffering [100]	Spain [100]

Table 3 also reiterates the notion that factors affecting patients’ perceptions of dignity are multi-faceted [182], and often impact all four rings of the RToP.

### Domain 3: Dignity conserving care

Dignity conserving care tends to be holistic and involves many if not all of the rings of the RToP. These are summarised in Table 4 for ease of review. The efficacy of these interventions rely on awareness of cultural sensitivities [85, 161], multidisciplinary team support [168, 183], effective communication [82, 96, 97] and appropriate infrastructure [93, 184]. Most of these interventions have a positive impact though five articles reported some of the negative outcomes.

**Table 4:** Dignity conserving practices

Rings	Practices	Outcomes	Facilitators	Barriers
Innate (*n* = 11)	Respect for spirituality [78, 93, 123, 147, 156, 175, 179, 185, 186] Spiritual comfort [78, 93, 123, 147, 156, 175, 179, 185, 186] Spiritual beliefs and practices [87, 110, 121, 122, 126]	Increased sense of dignity [122, 179] Improvement in quality of life [93, 121, 123, 156, 185–187]		
	Recognition as a person [97, 156, 188]	Facilitating individualism [156]		
Individual (*n* = 64)	Physical care [75, 85, 93, 143, 147, 169, 170, 173, 175, 189, 190] Symptomatic management [75, 85, 93, 143, 147, 169, 170, 173, 175, 189] Multidisciplinary/holistic care [143, 175, 189, 190]	Increased sense of dignity [143] Improvement in quality of care [189] Improvement in quality of life [175, 176]		
	Active participation in end of life [75, 78, 87, 93, 98, 101, 116, 125, 141, 147, 170, 175, 189, 191–193] Preference for care and death locations [93, 101, 116, 141, 175, 189, 191] Maintaining self-identity [78, 93, 98, 147, 170] Encouraging independence [147, 175] Self-coping mechanisms [78, 87, 147] Addressing aftermath concerns [75, 125, 192, 193]	Increased sense of dignity [110, 140, 141, 144, 194] Improvement in quality of care [124, 144, 164, 194] Improvement in quality of life [82, 93, 140] Facilitating individualism [140, 179]	Public Allowing patients to be cared for at home [80] Government legislations [145] Allowing advanced care planning [181] End-of-life regulations [145]	
	Psychosocial care [10, 75, 78, 82, 89, 92, 93, 96–98, 101, 102, 110, 112, 115–117, 119, 120, 123, 127, 131, 134, 136, 139, 142, 146, 147, 149, 150, 155, 156, 158, 164, 165, 169, 170, 173–181, 184–186, 188–190, 194–203] Good communication with patients [75, 78, 82, 92, 93, 98, 102, 139, 147, 149, 155, 173, 177–179, 188–190, 195, 196] Acknowledging personhood [75, 82, 89, 93, 98, 142, 147, 164, 170, 174–178, 188, 195, 196] Maintaining morale [93, 127, 147, 196] Environmental factors [89, 92, 93, 96, 116] Psychotherapy [10, 93, 98, 110, 115, 117, 119, 120, 123, 131, 134, 136, 139, 146, 147, 150, 156, 158, 165, 180, 181, 184–186, 194–203] Improving healthcare systems [97, 101, 112, 142, 169, 176, 190]	Increased sense of dignity [75, 110, 112, 114, 117, 131, 142, 147, 149, 150, 156, 179, 180, 185, 186, 188, 195, 197, 198, 200, 202] Improvement in quality of care [96, 142, 155, 164] Improvement in quality of life [96, 134, 156, 185, 186, 197, 202] Facilitating individualism [156, 180, 199] No significant effect of intervention [117, 120, 156] Long duration of therapy [117] Having a coherent view [131, 150] Improved respect for autonomy [119, 196] Heightened morale [93, 96, 98, 102, 117, 131, 147, 149, 150, 156, 175, 180, 186, 195–199, 201, 202] Feeling valued [93, 96, 102, 150, 156, 175, 180, 198] Increased sense of meaning [117, 131, 149, 150, 180, 186, 197, 198, 201, 202] Increased will to live [147, 149, 186, 198, 199, 202] Improved mood [115, 119, 149, 180, 186, 190, 201, 202] Increased self esteem [93, 98, 195, 196] Increased preparedness for death [75, 98, 119, 131, 147, 156, 175, 180, 194, 195, 198, 199, 202] Addressing aftermath concerns [75, 156, 180, 198, 199] Acceptance of death [98, 131, 156, 175, 194, 195, 202]		Public Conflicting views on patients’ dignity between healthcare providers and patients [204] Cultural ideologies [85, 161] oSuperstition about discussing death arrangements [161] Patients being in denial about dying [85, 161]
Relational (*n* = 31)	Preservation of familial bonds [98, 103, 122, 147, 188] Care and support from family [98, 103, 122, 188] Addressing aftermath concerns [93, 147] Retaining familial roles [93]	Increased sense of dignity [122] Making patient feel valued [103, 188] Assisting in communication [139]		Public Conflicting views on patients’ dignity between families and patients [204]
	Improving healthcare accessibility for families [75, 85, 87, 93, 124, 142, 177, 189] Availability to family [85, 93, 142] Good communication with patients’ families [75] Research involving relatives’ perspectives [142] Family engagement in patient care [87, 93, 124, 177, 189]	Increased sense of dignity [75, 87] Improvement in quality of care [142] Consoling patients [85] Improved connectedness with families [124]		
	Psychosocial care [115, 117, 119, 131, 139, 146, 156, 165, 180, 186, 194, 195, 197–202] Psychotherapy [115, 117, 131, 146, 156, 165, 180, 186, 194, 195, 197–202] Music therapy with family [139] Supporting patients’ self esteem [195]	Increased sense of dignity [117, 131, 156, 180, 186, 195, 197, 198, 200, 202] Improvement in quality of life [197, 202] Painting a distorted picture of the patient [198] Improved connectedness [139, 156, 180, 194, 195, 199] Within families [139, 156, 194, 195, 199] Discussing hopes and dreams for loved ones [180] More openness about patients’ condition [194] Increased preparedness for death [119, 180, 198] Preparing families for future [119, 180, 198]		
Societal	Social support [114, 119, 127, 142, 147, 184, 203] Psychotherapy [119, 184, 203] Prevention of demoralisation [127]	Increased sense of dignity [142] Improvement in quality of care [142] Awkward social settings [184]	Healthcare systems Good infrastructure oSocial support [93, 184] Supporting patients’ privacy [93] Quality improvement projects [96] Use of technology [168]	Public Poor social support [118] Healthcare systems Lack of psychosocial support in healthcare services [168]
	Social respect for patients [93, 97, 138, 142, 149] Good communication [149] Mutual respect [97] Preservation of patients’ roles [93, 138] Respecting social differences [93, 142]	Increased sense of dignity [138, 142] Facilitating individualism [97, 149]		Public Conflicting views on patients’ dignity between cultures [197] Patients feeling ostracised in public settings [99, 122] Patients being called an “economic burden to society” [81]
General			Healthcare systems Educational programs for healthcare providers [101] Understanding cultural differences [101] Improving communication techniques among healthcare providers [96] Standardised framework to address patient concerns [155] Multidisciplinary teamwork [168, 183]	Public Poor public policies [99] Healthcare systems Poor infrastructure [6] Poorly maintained physical environment [6] Limited human resource allocations [92, 96, 127] Long waiting times [95, 117] Lack of time for patients [96] Fast paced interactions [96] Use of technology [96] Busy schedules of healthcare workers [97] Long duration for therapy [194] Hospitals as a location for end-of-life care [124, 140]

### Stage 5 of SEBA: Analysis of data and non-data driven literature

Most of the articles included were data driven (87 out of 127), while the remaining articles were non-data-based articles (grey literature, opinion, perspectives, editorial, letters). The expert team and stakeholders raised concerns that data from grey literature, which was neither quality-assessed nor necessarily evidence-based could be a source of bias during the crafting of the discussion. As a result of these concerns, the research team thematically analysed data from grey literature and non-research-based pieces such as letters, opinion and perspective pieces, commentaries and editorials included in this review. The themes identified were compared against themes drawn from peer reviewed evidenced based data. This analysis revealed no differences in the themes from the two sources of data.

In addition, the research team employed the Medical Education Research Study Quality Instrument (MERSQI) [205] and the Consolidated Criteria for Reporting Qualitative Studies (COREQ) [206] to evaluate the quality of qualitative and quantitative studies included in this review (Supplementary File 1).

### Stage 6 of SEBA: Synthesis of the discussion

The discussion of this paper is framed around the domains identified in Stage 4 and is guided by the Best Evidence Medical Education (BEME) Collaboration guide [207] and the STORIES (Structured approach to the Reporting In healthcare education of Evidence Synthesis) statement [208].

## Discussion

In answering its primary and secondary research questions, this SSR in SEBA reveals that current patient defined concepts of dignity are intrinsically rooted within self-concepts of personhood and identity. Here there are core aspects to this sociocultural construct with concepts of dignity across different settings acknowledging that dignity be framed as the right to be treated as autonomous individual deserving of respect and care in a manner that is in keeping with their beliefs, values, self-concepts and changing needs simply by virtue of their status as a human being and irrespective of their circumstances [80, 99]. It is upon this platform that Chochinov, Krisjanson [7] ’s concept of dignity as “individualistic, transient, and often tied to personal goals and social circumstances”, and Street and Kissane [6] ’s notion of dignity as “relational and embodied ideas”, are built upon. [174] ’s concept of dignity as a function of “inherent” and “imputed” facets captures this notion. Robinson, Phipps [174], define “inherent dignity” as being intrinsic to all humans and suggest that this concept is individualized by “imputed dignity” where an individual refines and builds upon this notion using their narratives, values, beliefs and principles. The RToP provides a means of elucidating and contending with this nuanced perspective.

Echoing current concepts of dignity the RToP underscores the notion that a patient’s concept of dignity is both individual and evolving, changing over time and circumstances, and shaped by individual experiences, sociocultural circumstances, disease trajectory, setting, needs, and concepts of personhood and dignity [152, 209, 210]. However more significantly the RToP lens allows HCPs to determine which of the Innate, Individual, Relational and Societal rings dominate thinking and what elements within them need particular attention at a particular moment and context. Here the complexity of these evolving concepts underlines the need for a personalized, holistic, and longitudinal approach that is best met by a well-trained, responsive multidisciplinary team. A multidisciplinary team will also be better able to support patients, their caregivers, and their loved ones longitudinally and in a timely and holistic manner that is in a manner that is consistent with their sociocultural identities, spiritual needs, and self-concepts of their personhood [7, 88, 95, 97, 102, 103, 154].

Perhaps just as significantly a multidisciplinary team would also be better able to provide timely and regular appraisal, support and and follow-up of patients and their families throughout their illness journey [7, 88, 95, 97, 102, 103, 154]. Here the RToP may be employed as a tool to assess a patient’s concepts of dignity in different circumstances and at different timepoints along their disease trajectory. Mapping these changes over time would be especially useful at the end of life care when responsive, accessible, empathetic and personalised communications and personalised support is especially critical.

It is here in considering the design, study and longitudinal use of an adapted RToP based tool that the role of the host organisation becomes clear. It is the host organisation that must ensure an effective infrastructure that trains and supports the multidisciplinary team, an accessible and robust communication pathway and the support needed to evaluate and address the patient’s needs and goals.

### Limitations

One of the main limitations of this study was its inability to differentiate personalised concepts of dignity amongst a wide array of patients replete with their particular circumstances, sociocultural and healthcare settings. This is further limited by confining our review to publications in English or had English translations. Of the 127 included articles, most were from the West and especially the United Kingdom, United States of America, and Canada. This could skew our data collected on patients’ perceptions towards Western-centric ideals, underrepresenting perceptions more commonly seen in other areas of the world.

Moreover, whilst this study was intended to analyse the wide range of current literature on concepts of dignity, our review was limited by a lack of clear reporting of current dignity preserving measures nor of due consideration of resource limitations in a wide array of practices.

We also acknowledge that whilst taking into account the limited resources and availability of the research and experts teams in this review limiting the scope of this SSR in SEBA to the specified dates to increase the chances of completing the review, could have seen important articles excluded.

## Conclusions

This SSR in SEBA reiterates the posit that there are common elements to prevailing concepts of dignity and that a patient’s individualised concept of dignity is a refinement of this concept. In doing so this review underscores the need for a tool and a multidsicplinary approach to dignity conserving care especially at the end of life. As we look forward to continuing our engagement with this this critical aspect of clinical care, we look forward to further insights into this topic that can guide design and pilot a RToP-based as a tool to help HCPs understand their patient’s needs and attend to them in a timely, personalised, and appropriate manner.

## Supplementary Information

**Additional file 1.** Tabulated summaries.

## Data Availability

All data generated or analysed during this review are included in this published article and its supplementary files.
