# Supplementary material for: A systematic scoping review on patients’ perceptions of dignity
Source: BMC Palliat Care. 2022 Jul 4;21:118. doi: 10.1186/s12904-022-01004-4 (PMC9251939; doi:10.1186/s12904-022-01004-4)
Supplement: Supplementary file 1 — Additional file 1. Tabulated summaries. [file 12904_2022_1004_MOESM1_ESM.pdf]

Supplementary File 1. Tabulated Summaries

Author	Year Published	Study Design	Title	Intro	Methods	Results	Conclusion	Quality Assessment	
								MERSQI score	COREQ score
1. Luigi Grassi et al.	2016	Cross Sectional	Dignity and psychosocial-related variables in advanced and non-advanced cancer patients by using the Patient Dignity Inventory	The Patient Dignity Inventory (PDI) is a valid and reliable instrument to measure dignity, as a state of physical, mental, social and spiritual wellbeing in palliative care patients and an essential dimension for a comprehensive patient-centered approach. We examined the factor structure and the correlation of the Italian version of the PDI (PDI-IT) with psychosocial variables among advanced and non-advanced cancer outpatients in two Italian centers.	In a sample of 194 patients principal component analysis, reliability analysis (Cronbach's coefficient alpha), and correlation analysis of the PDI-IT were performed. Concurrent validity was evaluated with respect to the Italian versions of Patient Health Questionnaire–9 (PHQ-9), as a measure of depression, the Mini-Mental Adjustment to Cancer–Hopelessness scale (Mini-MACHH), as a measure of dysfunctional coping, and the Demoralization scale (DS-IT), as a measure of demoralization.	Three factors were extracted by exploratory factor analysis, which accounted for 64.38% of the variance, namely Existential distress (Cronbach's α=0.95), Psychological Distress (Cronbach's α=0.88), and Physical Distress (Cronbach's α=0.81), with a Cronbach's α coefficient for the PDI-IT Total score of 0.96. PDI-IT factors were significantly inter-correlated and shared between 42% and 53% of the variance. Higher scores on all the PDI-IT factors and PDI-IT Total were found among patients who were clinically depressed (PHQ-9) and among those who were demoralized on the DS-IT. Significant correlations were also found between all PDI-IT and DSIIT,	The study confirmed that the PDI-IT is a valid instrument to be applied in oncology and measuring three factors, namely existential, psychological and physical distress, as core dimensions of dignity, to be monitored and treated in clinical settings.	12.5	-

						PHQ-9, and Mini-MAC-HH.			
2.	L. Wang et al.	2019	Cross Sectional	Dignity and its influencing factors in patients with cancer in North China: a cross-sectional study	Patients with cancer experience various levels of loss of dignity. Exploring levels of loss of dignity and the factors that influence such losses for patients with cancer is rare, but important in palliative care in China.	Participants were cancer patients with early and advanced cancer recruited from a tertiary cancer hospital in North China. Patients were surveyed to assess their level of loss of dignity and potentially relevant factors. Data were collected using the Patient Dignity Inventory, the MD Anderson Symptom Inventory–Chinese, the distress thermometer, the Hospital Anxiety and Depression Scale, and the 30-question core Quality of Life Questionnaire from the European Organisation for Research and Treatment of Cancer, and were analyzed using quantitative methods.	The study included 202 cancer patients, 143 of whom experienced mild loss of dignity (71%); 37, moderate loss of dignity (18%); and 10, severe loss of dignity (5%). The problems with dignity were slightly different in patients with early-stage disease than in those with advanced-stage disease. Loss of dignity in the patients was significantly correlated with psychological distress, symptom burden, and quality of life ($p < 0.05$). Logistic regression showed that age, Karnofsky performance status, anxiety, and symptom burden were significant predictors of loss of dignity.	Most patients with early and advanced cancer experienced some level of loss of dignity. Loss of dignity was more likely for patients of younger age, high Karnofsky performance status, high symptom burden, and anxiety. Understanding the dignity of cancer patients and potentially relevant factors is of great value for implementing comprehensive palliative care in China.	11	-
3.	Harvey Max Chochinov et al.	2016	Cohort	Dignity and Distress towards the End of Life across Four Non-Cancer Populations	The purpose of this study was to identify four non-cancer populations that might benefit from a palliative approach; and describe and compare the prevalence and patterns of dignity	A prospective, multi-site approach was used.	Between February 2009 and December 2012, 404 participants were recruited (ALS, 101; COPD, 100; ESRD, 101; and frail elderly, 102). Depending on group designation, 35% to	People with ALS, COPD, ESRD and the frail elderly face unique challenges as they move towards the end of life. Knowing the intricacies of distress and how	14	-

				related distress across these diverse clinical populations.		58% died within one year of taking part in the study. While moderate to severe loss of sense of dignity did not differ significantly across the four study populations (4–11%), the number of PDI items reported as problematic was significantly different i.e. ALS 6.2 (5.2), COPD 5.6 (5.9), frail elderly 3.0 (4.4) and ESRD 2.3 (3.9) [p < .0001]. Each of the study populations also revealed unique and distinct patterns of physical, psychological and existential distress.	they differ across these groups broadens our understanding of end-of-life experience within non-cancer populations and how best to meet their palliative care needs.		
4. Annette F. Street et al.	2012	Qualitative Content Analysis	Dignity and Deferral Narratives as Strategies in Facilitated Technology-Based Support Groups for People with Advanced Cancer	This paper examines the value of facilitated telephone and online support groups for palliative care.	Telephone interviews were conducted with twenty people living with advanced cancer who had participated in either a telephone or online support group facilitated by the Cancer Council Victoria, Melbourne, Australia.	Two dominant participant narratives emerged: a focus on dying with dignity or an interest in deferring discussion of death and dying to focus on the present. Despite the different approaches, participants found the technology-based support groups to be accessible and safe environments in which to discuss difficult topics in privacy.	-	-	16

						Technology-based strategies provide opportunities for health professionals to provide social and emotional care to more people by moving beyond individualised care and facilitate peer-to-peer support at the end of life, especially to those with specific needs. Such options are feasible for palliative care services to set up and acceptable to a group of clients, especially for younger clients or those socially or geographically isolated.			
5. Andy H. Y. Ho et al.	2013	Qualitative Ethnographic	Dignity Amidst Liminality: Healing Within Suffering Among Chinese Terminal Cancer Patients	This study critically examines the concepts of dignity and liminality at the end-oflife, in an effort to better understand the processes of healing within suffering among Chinese terminal cancer patients receiving palliative care services in Hong Kong.	Meaning-oriented interviews were conducted with 18 Chinese terminal patients, aged 44 to 98, to elicit the narratives and stories of their illness experience. All interviews were analyzed using grounded theory and supplemented by ethnographic observations and field notes.	Two major themes and eight subprocesses of healing adopted by patients to achieve and maintain dignity were identified: (a) personal autonomy, which encompasses the need to (i) regain control over living environments, (ii) maintain self-sufficiency despite institutional care, (ii) make informed care decisions to reduce sense of	Implications of these themes for advanced care planning and life review interventions were discussed with the goal of enhancing patient autonomy and family connectedness, and thereby providing structure and meaning for Chinese terminal patients and their families at the end of life.	-	20

						burden, and (iv) engage in future planning to create a lasting legacy; and (b) family connectedness, which encompasses the need to (i) maintain close ties with family members to express appreciation, (ii) achieve reconciliation, (iii) fulfill family obligations, and (iv) establish a continuing bond that transcends generations.			
6. X de Voogd et al.	2020	Qualitative Thematic Analysis Of Semi-Structured Interviews	A dignified last phase of life for patients with a migration background: A qualitative study	Preserving personal dignity is an important part of palliative care. Generally, autonomy, independency and not being a burden to others are emphasised for preserving dignity. Dignity has not been studied yet from the perspective of the growing group of patients with a migration background living in Western countries. Aim: To gain insight into (1) what patients – and their relatives – with a Turkish, Moroccan or Surinamese background, living in the Netherlands, in their last phase of life find important aspects of dignity,	Design: Qualitative thematic analysis of semi-structured interviews.	Results: For respondents dignity encompassed surrender to God's or Allah's will and meaningful relationships with others, rather than preserving autonomy. Surrender to God or Allah meant accepting the illness, the situation and performing religious practice. A meaningful relationship meant being assisted or cared for by family members and maintaining a social role. Professionals could preserve dignity by showing respect and attention;	Conclusions: Religion and appropriate involvement of family members are important aspects of dignity in the last phase of life, in addition to autonomy and independency. Care professionals need to take these factors into account in order to provide person-centred care.	-	19

				and (2) how care professionals can preserve and strengthen the dignity of these patients.		guaranteeing physical integrity, hygiene and self-direction; and indirect communication about diagnoses and prognoses.			
7.	Isis E. van Gennip et al.	2013	Qualitative Interview Study	The development of a model of dignity in illness based on qualitative interviews with seriously ill patients	Background: While knowledge on factors affecting personal dignity of patients nearing death is quite substantial, far less is known about how patients living with a serious disease understand dignity. Objective: To develop a conceptual model of dignity that illuminates the process by which serious illness can undermine patients' dignity, and that is applicable to a wide patient population.	Qualitative interview study. In-depth interviews were carried out exploring the experiences of seriously ill patients with regard to their personal dignity. The interview transcripts were analyzed using thematic analysis and a conceptual model was constructed based on the resulting themes.	We developed a two-step dignity model of illness. According to this model, illness related conditions do not affect patients' dignity directly but indirectly by affecting the way patients perceive themselves. We identified three components shaping self-perception: (a) the individual self: the subjective experiences and internally held qualities of the patient; (b) the relational self: the self within reciprocal interaction with others; and, (c) the societal self: the self as a social object in the eyes of others.	The merits of the model are two-folded. First, it offers an organizing framework for further research into patients' dignity. Secondly, the model can serve to facilitate care for seriously ill patients in practice by providing insight into illness and dignity at the level of the individual patient where intervention can be effectively targeted.	-	22
8.	Ping Ying Choo et al.	2020	Systematic Review	Reciprocal Dynamics of Dignity in End-of-Life Care: A Multiperspective Systematic Review of Qualitative and Mixed	Background: Preserving terminally ill patients' dignity and well-being through dignified and holistic care has become the overarching goal in palliative care services. However, dignity is a	Design: This systematic review adhered to the Preferred Reporting Items for Systematic Reviews and Meta-Analyses guideline and used SPIDER tool to screen for appropriate and	Results: Analysis of the various concepts of dignity revealed 18 themes that were further categorized into 7 conceptual categories: (1) self-determination, (2) existential liberty, (3) relational	Conclusions: The Dynamic Reciprocity of Dignity model highlights the importance of adopting a systemic lens to address dignity-related needs and concerns at the	-	-

			Methods Research	multifaceted concept with a wide range of interpretations under different cultural contexts. Aim: The aim of this review is to understand the variations in subjective interpretations and constitutions of dignity in palliative or end-of-life care via an integrative worldview.	relevant articles for analysis.	connectedness, (4) caregiving revitalization, (5) mindful humanity, (6) patient–family care, and (7) sustainable culture. These 7 categories span across individual, familial, and institutional dimensions, forming a new Dynamic Reciprocity of Dignity model.	end of life, while providing insights on how compassionate care and self-compassion can serve as the foundation of dignified care, which in turn serve as a buffer against patients' existential suffering as well as caregivers' burnout and fatigue. Recommendations for clinical practice and future research directions are discussed.		
9. S.M. Aoun	2015	Qualitative research design	Older people living alone at home with terminal cancer	This study describes the lived experiences of older people coping with terminal cancer and living alone, focusing on how they face challenges of the biographical life changes from their disease progression. Face-to-face semi-structured interviews were conducted in two phases with palliative care clients of a community-based service in Western Australia	Brief interviews with 43 cancer patients who live alone and then in-depth interviews with 8 of them.	Using biographical disruption as the analytical framework for interpreting the qualitative data, four main themes emerged: Biographical disruption: adjusting to change; Biographical continuity: preserving normality; Biographical reconstruction: redefining normality; and Biographical closure: facing the end. Biographical disruption was a suitable framework for	Understanding the factors associated with the individual's need to maintain their own identity will enable nurses working with this population to tailor support plans that meet the individuals' needs while maintaining or restoring the person's sense of self. Interventions that directly address end-of-life suffering and bolster sense of dignity and personhood need to be considered.	-	18

						analysis, permitting identification of the biographical disruptions of the individual's world and the reframing that is undertaken by the individual to maintain autonomy and independence while acknowledging and accepting their closeness to death.			
10. Sandra Martins Pereira	2015	Case Series	Old age and forgoing treatment: a nationwide mortality follow-back study in the Netherlands	The ageing of the population raises the need to study forgoing treatment decisions among older people.	A nationwide study of a stratified sample from the Statistics Netherlands death registry to which all deaths were reported in 2010. All attending physicians<br><br>of those deaths received a questionnaire about end-of-life decisions. 6600 cases were studied. We examined<br><br>three age groups: 17–64, 65–79, and 80 and above. Logistic regression analyses were performed to identify age-related differences controlling for other patient characteristics.	Forgoing treatment occurred in 37% of the total population, with a significant increase in the incidence across age. The most common treatments withheld/withdrawn were artificial hydration/nutrition, medication and antibiotics. Age-related differences were found, especially for withholding artificial hydration/ nutrition among patients aged 65–79 (OR 2.04), and for withdrawing medication (OR 2.51) and antibiotics (OR 2.10) among the oldest when compared to the youngest patients. The most common reason for making the	Forgoing treatment occurred in a substantial proportion of older people, and more often than in younger age groups. The avoidance of burdensome treatment solely to prolong life suggests a better acceptance that these patients are nearing death.	14	-

						decision was ‘no chance of improvement’. The likelihood of forgoing treatment due to ‘loss of dignity’ was higher for the oldest (OR 2.32), as well as due to the request/ wish of the patient (OR 1.97), when compared to the youngest patients.			
11. Sue Hall	2015	Randomized Control Trial	A novel approach to enhancing hope in patients with advanced cancer: a randomised phase ii trial of dignity therapy	To assess the ability of dignity therapy to reduce distress in advanced cancer patients. Design A phase II open-label trial. Setting Two UK National Health Service trusts. Intervention Dignity therapy: a brief palliative care psychotherapy.	Participants were randomly allocated to receive the intervention plus standard care or standard care only (control group). Outcomes were collected at baseline and at 1- and 4-week follow-up.	The primary outcome was dignity-related distress (Palliative Dignity Inventory). Secondary outcomes were hope, anxiety and depression, quality of life, palliative-related outcomes, and self-reported study benefits. 45/188 (24%) patients responded. 27/45 (60%) participants remained at 1-week and 20/45 (44%) at 4-week follow-up. Baseline levels of distress were low. Groups did not differ in dignity-related distress at any time. An effect on only one secondary outcome was found: the intervention group reported more hope than the control group at	The effects of dignity therapy on people with advanced cancer are encouraging. Further investigation is warranted focusing on distressed patients and those earlier in the palliative care trajectory.	13.5	-

						both follow-ups. Effect sizes were medium (partial η2<br><br>=0.20 and 0.15) and the difference was statistically signifi cant at 1-week follow-up (difference in adjusted means 2.55; 95% CI −4.73 to 0.36; p=0.02). The intervention group was more positive than the control group on all the self-reported benefi ts ratings. Effect sizes (Cohen's d) ranged from 1.34 for feeling that dignity therapy had helped to 0.31 for increasing will to live.			
12. Mary Fran Tracy et al	2007	Editorial	Upholding Dignity in Hospitalized Elders	Caring for the elderly in today's acute care setting can be a challenging and complex process. The elderly have unique needs that may not always be obvious. In addition, elderly frequently exhibit a myriad of needs and can be frail with less resilient compensatory reserves. Regardless of whether the interventions provided by caregivers are physical or psychosocial, one of					

				the basic tenets of caring for elderly is to uphold their dignity. This article defines the concept of dignity, describes challenges to maintaining dignity for elderly in an acute care setting, and describes interventions that are key to maintaining dignity. In addition, strategies to uphold dignity are described and recommendations are made for education, practice, research, and policy development in the area of upholding dignity for hospitalized elderly.					
13. Jennifer Thorley	2015	Opinion	Too many die without dignity	Many patients in the UK do not receive the end-of-life care they need, according to a report published on May 20, by the Parliamentary and Health Service's Ombudsman Julie Mellor.	-	-	-	-	-
14. Frank Brennan	2017	Editorial	"To Die with dignity": an update on palliative care	Significant developments have occurred in the discipline of palliative care in the modern era. This paper shall explore those developments, challenge some widely held misconceptions about the role and daily practice of the	-	-	-	-	-

				discipline, highlight the growing recognition of the role of palliative care in non-malignant diseases, briefly discuss innovations in symptom management and reflect on the underlying principles, maturation and challenges faced by the discipline.					
15. Samara Gonçalves de Oliveiral et al.	2020	Systematic review	Bioethical aspects of health care provided to older adults at the end of their lives	To examine the Brazilian and international scientific production for connections between bioethical principles and the health care provided to older adults at the end of their lives.	Between October and November 2019 this integrative review searched the Medline (Pubmed), Lilacs, and Scopus databases using the descriptors: “Palliative Care”, “Aged”, and “Bioethics” for the period 2014-2019	The following categories emerged from the selected articles: therapeutic conducted with regard to end-of-life care; decision making on end-of-life care; and challenges in end-of-life care. Conclusion: of particular importance is for health professionals to uphold their commitment to older adults and their families, consider their subjectivities and preferences, and empower and equip them so that care is guided by bioethical principles in order to assure a dignified process of dying and death.	-	-	-
16. Mathias Wirth	2016	Editorial	Awareness and Dying: The Problem of Sedating ‘Existential	The number of people who die while sedated is increasing, in part due to a currently widespread conviction that dying	-	-	-	-	-

			Suffering' in Palliative Care	brings about paucity of meaning and is seen as an intolerable situation for the person affected. Palliative care tends to affirm this attitude towards dying and denying when it uses terminal sedation in cases of 'existential suffering'. We hope to launch an interdisciplinary discussion on how Meaning-Maintenance and Dignity-Therapy can help support an 'active dying phase' and encourage caregivers not to uphold sedation as a possible standard of palliative medicine. This is followed by a dialogue between ethics of palliative medicine and medical psychology, which reaffirms the last-resort character of terminal sedation.					
17. M. Gysels et al.	2016	Cross-Sectional	Dignity through integrated symptom management: lessons from the Breathlessness Support Service	Dignity is poorly conceptualized and little empirically explored in end of life care. A qualitative evaluation of a service offering integrated palliative and respiratory care for patients with advanced disease and refractory breathlessness uncovered an	Qualitative study of cross-sectional interviews with 20 patients as part of a phase III evaluation of a randomized controlled fast-track trial. The interviews were transcribed verbatim, imported into NVivo, and analysed through constant	The findings of this study underscore the applicability of the conceptual model of dignity for patients with breathlessness. There were many similarities in themes and sub-themes. Differences specifically relevant for patients suffering from severe breathlessness	Dignity is an integrated concept and can be affected by influences from other areas such as illness-related concerns. The intervention shows that targeting the symptom holistically and equipping patients with the means for	-	17

				unexpected outcome, it enhanced patients' dignity. To analyse what constitutes dignity for people suffering from refractory breathlessness with advanced disease, and its implications for the concept of dignity.	comparison. The findings were compared with Chochinov et al.'s dignity model. The model was adapted with the themes and sub-themes specific to patients suffering from breathlessness.	were: a. Physical distress and psychological mechanisms are interlinked with the disability and dependence breathlessness causes, in the illness-related concerns; b. Stigma is an important component of the social dignity inventory;<br>c. Conditions and perspectives need to be present to practice self-care in the dignity conserving repertoire.	self-care, realized the outcome of dignity.		
18. Susan McClement, et al.	2007	Cohort Study	Dignity Therapy: Family Member Perspectives	Dignity Therapy is a novel therapeutic intervention designed to address psychosocial and existential distress among the terminally ill. This brief, individualized approach to end-of-life care invites patients to discuss issues that are most important to them and to articulate things they would most want remembered as death draws near. These discussions and recollections are recorded, transcribed, and edited into a generativity document, which are usually given to	Sixty family members of deceased terminally ill patients who previously took part in Dignity Therapy completed a questionnaire to elicit feedback about the impact of Dignity Therapy on both the dying patient and themselves.	Ninety-five percent of participants reported that Dignity Therapy helped the patient;<br>78% reported that it heightened the patient's sense of dignity; 72% reported that it heightened the patient's sense of purpose; 65% reported that it helped the patient prepare for death; 65% reported that it was as important as any other aspect of the patient's care; and 43% reported that Dignity Therapy reduced the patient's suffering. Regarding	Family members endorse Dignity Therapy as a therapeutic intervention that moderates their bereavement experiences and lessens suffering and distress in terminally ill relatives.	14	-

				family or loved ones. While the marked benefits of Dignity Therapy on patients' psychosocial and existential distress have been reported elsewhere, this paper presents data on bereft family members' perspectives regarding the impact of dignity therapy on patients and themselves.		family members, 78% reported that the generativity document helped them during their time of grief; 77% reported that the document would continue to be a source of comfort for their families and themselves; and 95% reported they would recommend Dignity Therapy to other patients of family members confronting a terminal illness.			
19. Brenda Bentley et al.	2014	Cross Sectional	Feasibility, Acceptability, and Potential Effectiveness of Dignity Therapy for People with Motor Neurone Disease	Motor neurone disease (MND) practice guidelines suggest developing interventions that will promote hope, meaning, and dignity to alleviate psychological distress, but very little research has been done. This study begins to address this need by exploring the use of dignity therapy with people with MND. Dignity therapy is a brief psychotherapy that promotes hope, meaning and dignity, and enhances the end of life for people with advanced cancer. The aims of this study are to assess the feasibility,	This cross-sectional feasibility study used a one-group pre-test post-test design with 29 people diagnosed with MND. Study participants completed the following self-report questionnaires: Herth Hope Index, FACIT-sp, Patient Dignity Inventory, ALS Assessment Questionnaire, ALS Cognitive Behavioural Screen, and a demographic and health history questionnaire. Acceptability was measured with a 25-item feedback questionnaire. Feasibility was	There were no significant pre-test post-test changes for hopefulness, spirituality or dignity on the group level, but there were changes in hopefulness on the individual level. The results of the feedback questionnaire indicates dignity therapy is highly acceptable to people with MND, who report benefits similar to those in the international randomised controlled trial on dignity therapy, a population who primarily had end-stage cancer. Benefits include better family	Dignity therapy for people with MND is feasible and acceptable. Further research is warranted to explore its ability to diminish distress	13.5	-

				acceptability, and potential effectiveness of dignity therapy for people with MND.	assessed by examining the length of time taken to complete dignity therapy and how symptoms common in MND affected the intervention. Generalised linear mixed models and reliable change scores were used to analyse the data	relationships, improved sense of self and greater acceptance. Dignity therapy with people with MND is feasible if the therapist can overcome time and communication difficulties.			
20. Lori Montross et al.	2011	Cross Sectional	Dignity Therapy Implementation in a Community-Based Hospice Setting	Dignity Therapy is a brief, empirically supported, individualized psychotherapy designed to address legacy needs among patients at the end of life. To date, this psychotherapy has not been implemented in a ‘‘real-world’’ community-based hospice setting. This study was designed to offer information about the pragmatic aspects of implementing Dignity Therapy for patients receiving hospice care.	Twenty-seven patients completed Dignity Therapy as part of a clinical service newly offered at a community-based hospice. Referral and enrolment procedures, as well as the logistics of therapy implementation were monitored. Patients' legacy transcripts were also qualitatively analysed to measure emergent themes	Patients were most commonly referred by social workers, and on average produced Dignity Therapy legacy transcripts approximately 3000 words/8 pages in length. The mean number of sessions spent with patients was 4, equating to an average of 380 minutes of clinician time per patient. Qualitative analyses revealed the most commonly discussed topics among patients were (in rank order): autobiographical information, love, lessons learned in life, defining roles in vocations or hobbies, accomplishments, character traits, unfinished business, hopes and dreams,	This was the first study to implement Dignity Therapy in a community sample, with results highlighting the practical aspects of treatment as well as the most common themes discussed by clinical patients in a hospice setting. These findings provide useful data for clinicians or organizational leaders who may consider offering Dignity Therapy in their setting, and offer general insight regarding the legacy topics most frequently discussed by patients near the end of life.	13	-

						catalysts, overcoming challenges, and guidance for others.			
21. Luca Iani et al.	2020	Randomized controlled trial	Dignity Therapy Helps Terminally Ill Patients Maintain a Sense of Peace: Early Results of a Randomized Controlled Trial	Dignity Therapy (DT) is a brief, individualized, narrative psychotherapy developed to reduce psychosocial and existential distress, and promote dignity, meaning, and hope in end of life patients. Previous studies have shown that DT was effective in reducing anxiety and depression, and improving dignity-related distress. However, less is known about its efficacy on spiritual well-being. The aim of this study is to contribute to the existing literature by investigating the effects of DT on specific dimensions of spiritual well-being, demoralization and dignity-related distress in a sample of terminally ill patients.	A randomized, controlled trial was conducted with 64 terminally ill patients who were randomly assigned to the intervention group (DT + standard palliative care) or the control group (standard palliative care alone). The primary outcome measures were Meaning, Peace, and Faith whereas the secondary outcome measures were (loss of) Meaning and purpose, Distress and coping ability, Existential distress, Psychological distress, and Physical distress. All measures were assessed at baseline (before the intervention), 7–10 and 15–20 days after the baseline assessment. The trial was registered with ClinicalTrials.gov.	The MANOVA yielded a significant effect for the Group X Time interaction. ANOVA with repeated measures showed a significant effect of time on peace and a significant Group X Time interaction effect on peace. Post hoc comparisons revealed that, while there was a decrease in peace from pre-treatment to follow-up and from post-treatment to follow-up in the control group, there was no such trend in the intervention group.	This study provides initial evidence that patients in the DT intervention maintained similar levels of peace from pre-test to follow-up, whereas patients in the control group showed a decrease in peace during the same time period. We did not find significant longitudinal changes in measures of meaning, faith, loss of meaning and purpose, distress and coping ability, existential, psychological and physical distress. The findings of our study are of relevance in palliative care and suggest the potential clinical utility of DT, since they offer evidence for the importance of this intervention in maintaining peace of mind for terminally ill patients.	15.5	-

22.	Samar M. Aoun et al.	2015	Cross Sectional	Dignity Therapy for People with Motor Neurone Disease and their Family Caregivers: A Feasibility Study	Background: There are calls to explore psychological interventions to reduce distress in patients with motor neurone disease (MND) and their family caregivers. Dignity therapy is a short-term psychotherapy intervention shown to alleviate distress for people with life-limiting illnesses. Objectives: To assess the acceptability, feasibility, and effectiveness of dignity therapy to reduce distress in people with MND and their family caregivers.	The study used a repeated-measures design pre- and post-intervention. Acceptability and feasibility were assessed using participants' ratings of the helpfulness of the intervention across several domains and time and resources required. Effectiveness measures for patients included: dignity-related distress, hopefulness, and spiritual well-being; and those for family caregivers included burden, hopefulness, anxiety, and depression.	Twenty-seven patients and 18 family caregivers completed the intervention. Dignity therapy was well accepted, including those patients who required assisted communication devices. The feasibility may be limited in small or not well-resourced services. There were no significant differences in all outcome measures for both groups. However, the high satisfaction and endorsement of dignity therapy by patients suggests it has influenced various important aspects of end-of-life experience. Family caregivers overwhelmingly agreed that the dignity therapy document is and will continue to be a source of comfort to them and they would recommend dignity therapy to others in the same situation.	This is the first dignity therapy study to focus on MND and on home-based caregiving. Results established the importance of narrative and generativity for patients with MND and may open the door for other neurodegenerativ e conditions.	8	-
23.	Melissa B. Korman et al.	2021	Randomized Controlled Trial	Dignity therapy for patients with brain tumours: qualitative reports from patients, caregivers and practitioners	Background: Most individuals with brain tumours experience distress or cognitive impairment during the illness trajectory, potentially causing	Methods: Participants were recruited from the Odette Cancer Centre in Toronto. One of five Dignity Therapists	Results: Fifteen out of the 17 recruited participants (88%) completed the intervention; 2 were unable to complete the	Conclusions: The low attrition rate for the intervention suggests that DT is feasible in this population,	13	-

				decreased quality of life, strain on interpersonal relationships and altered sense of self or of the world. Symptoms of brain tumour and treatment can cause increased reliance on others and decreased in sense of dignity. Dignity is an important consideration when caring for patients, as it can influence decisions at end-of-life. Dignity therapy (DT) is a therapeutic intervention that was developed for patients near the end of life. DT encourages the patient to reflect on the life lived, including important roles and sources of pride, resulting in the development of a 'Legacy Document'. DT has been shown to enhance quality of life and dignity, and reduce psychological and existential distress for patients at the end-of-life. There is little literature on the effectiveness of DT, or other quality of life interventions, in brain tumour populations; This paper reports on the	conducted the intervention; time data was logged. Immediately after the intervention, patient participants, their caregivers, and Dignity Therapists were sent an open-ended, self-report survey about their experience with DT. Qualitative content analysis was conducted by an impartial reviewer. Average time taken to conduct the intervention was determined.	intervention due to progressing disease. Qualitative data was categorized according to two main areas of interest: Acceptability and Impact. Four participants, 5 caregivers and 4 care providers completed the qualitative surveys. All 4 patient participants reported benefits of DT that related to communication and/ or advanced care planning (ACP). Dignity therapists felt that the impact on their patients was positive, and reported satisfaction as a clinician.	though the required time to complete DT might be difficult for healthcare practitioners to provide within the recommended timeframe for this therapy. Positive qualitative reports on the effect of DT from patients, caregivers and dignity therapists alike indicate that DT is a promising intervention for this demographic.		

				feasibility of conducting DT with this population, and presents qualitative data gathered from patients with brain tumours who participated in DT, their caregivers, and their Dignity Therapists.					
24.	Harvey M. Chochinov et al.	2006	Cohort Study	Dignity in the Terminally Ill: Revisited	Background: Several studies have been conducted examining the notion of dignity and how it is understood and experienced by people as they approach death.<br><br>Objective: The purpose of this study was to use a quantitative approach to validate the Dignity Model, originally based on qualitative data.	Design: Themes and subthemes from the Dignity Model were used to devise 22 items; patients were asked the extent to which they believed these specific issues were or could be related to their sense of dignity.	Results: Of 211 patients receiving palliative care, “not being treated with respect or under-standing” (87.1%) and “feeling a burden to others” (87.1%) were the issues most identified as<br><br>having an influence on their sense of dignity. All but 1 of the 22 items were endorsed by more that half of the patients; 16 items were endorsed by more than 70% of the patients. Demographic variables such as gender, age, education, and religious affiliation had an influence on what items patients ascribed to their sense of dignity. “Feeling life no longer had meaning or purpose” was the only variable to enter a logistic	Conclusions: This study provides further evidence supporting the validity of the Dignity Model. Items contained within this model provide a broad and inclusive range of issues and concerns that may influence a dying patient’s sense of dignity. Sensitivity to these issues will draw care providers closer to being able to provide comprehensive, dignity conserving care.	15	-

						regression model predicting overall sense of dignity.			
25. Harvey Max Chochinov et al.	2002	Cross Sectional	Dignity in the terminally ill: a developing empirical model	Despite use of the term dignity in arguments for and against a patient's self-governance in matters pertaining to death, there is little empirical research on how this term has been used by patients who are nearing death. The objective of this study was to determine how dying patients understand and define the term dignity, in order to develop a model of dignity in the terminally ill.	semi-structured interview was designed to explore how patients cope with their advanced cancer and to detail their perceptions of dignity. Interviews were audiotaped and transcribed verbatim. A consecutive sample of 50 consenting patients with advanced terminal cancer were recruited over a 15-month period of time from an urban extended care hospital housing a specialized unit for palliative care. This unit provides both inpatient services, and coordinates end-of-life care community-based programming. Data were analysed using latent content analysis and constant comparison techniques. Four members of the research team independently coded the transcribed data, to develop conceptually	Three major categories emerged from the qualitative analysis, including illness-related concerns; dignity conserving repertoire; and social dignity inventory.	These broad categories and their carefully defined themes and sub-themes form the foundation for an emerging model of dignity amongst the dying. The concept of dignity and the dignity model offer a way of understanding how patients face advancing terminal illness.	-	21

					meaningful categories of responses. Operational definitions were written for major categories, themes and sub-themes.				
26. Harvey Max Chochinov et al.	2002	Cross Sectional	Dignity in the terminally ill: a cross-sectional, cohort study	Considerations of dignity are often raised in reference to the care of dying patients. However, little research that addresses this issue has been done. Our aim was to identify the extent to which dying patients perceive they are able to maintain a sense of dignity, and to ascertain how demographic and disease-specific variables relate to the issue of dignity in these individuals.	We did a cross-sectional study of a cohort of terminally ill patients with cancer, who had a life expectancy of less than 6 months. We enrolled 213 patients from two palliative care units in Winnipeg, Canada, and asked them to rate their sense of dignity. Our main outcome measures included: a 7-point sense of dignity item; the symptom distress scale; the McGill pain questionnaire; the index of independence in activities of daily living (IADL); a quality of life scale; a brief battery of self-report measures, including screening for desire for death, anxiety, hopelessness, and will to live; burden to others; and	16 of 213 patients (7·5%; 95% CI 4–11) indicated that loss of dignity was a great concern. These patients were far more than likely than the rest of the cohort to report psychological distress and symptom distress, heightened dependency needs, and loss of will to live.	Loss of dignity is closely associated with certain types of distress often seen among the terminally ill. Preservation of dignity should be an overall aim of treatment and care in patients who are nearing death.	13	-

					requirement for social support.				
27. Tove Bylund-Grenklo et al.	2019	Cross Sectional	Dignity in life and care: the perspectives of Swedish patients in a palliative care context	Background: How patients preserve their sense of dignity in life is an important area of palliative care that remains to be explored. Aims: To describe patients' perspectives of what constitutes a dignified life within a palliative care context.	Methods: Twelve palliative care patients were interviewed about their views on living with dignity. Data were analysed using qualitative content analysis.	Results: What constitutes a dignified life during end-of-life care was captured by the theme 'I may be ill but I am still a human being' and presented under the categories 'preserving my everyday life and personhood', 'having my human value maintained by others through 'coherence' and 'being supported by society at large'.	Conclusion: Patients' sense of dignity can be preserved by their own attitudes and behaviours, by others and through public support. Health professionals need to adopt a dignity-conserving approach, for which awareness of their own attitudes and behaviours is crucial.	-	13
28. Andrea Bovero et al.	2018	Cross Sectional	Dignity in cancer patients with a life expectancy of a few weeks. Implementation of the factor structure of the Patient Dignity Inventory and dignity assessment for a patient-centred clinical intervention: A cross-sectional study	Hospice is a favoured setting for dignity care. Studies on dignity dimension in end-of-life patients are growing. The Patient Dignity Inventory (PDI) is a tool that can lead to interesting information on dignity-related aspects of suffering. The study aimed to investigate dignity among end-of-life cancer patients, by examining the Italian version of the PDI factor structure and assessing the relationship between dignity and other	This is a cross-sectional study. Data were collected using a battery of self-administered validated rating scales. The sample included 127 hospice patients with a life expectancy of a few weeks and a Karnofsky Performance Status ≤40. Factor structure and concurrent validity of PDI and correlations between dignity and anxious and depressive symptomatology, quality of life,	Factor analysis highlighted a five-factor solution, accounting for 60% of the overall variance. The factors were labeled Psychological Distress, Social Support, Physical Symptoms and Dependency, Existential Distress, and Loss of Purpose/Meaning. Dignity assessment evidenced that self-blame coping style, emotional and physical well-being, and depression were the loss of dignity significant predictors	The results point out the intercultural validity of the PDI and empower an accurate detection of dignity-related distress sources in the daily clinical practice. Personality traits seem to have an active role in the loss of dignity, whereas spirituality is confirmed to be positively involved in dignity enhancement.	13	-

				patients' psychosocial and spiritual variables to improve a patient-centered clinical practice. Hospice is a favoured setting for dignity care. Studies on dignity dimension in end-of-life patients are growing. The Patient Dignity Inventory (PDI) is a tool that can lead to interesting information on dignity-related aspects of suffering. The study aimed to investigate dignity among end-of-life cancer patients, by examining the Italian version of the PDI factor structure and assessing the relationship between dignity and other patients' psychosocial and spiritual variables to improve a patient-centered clinical practice.	demoralization, personal coping styles, spiritual well-being, and spiritual daily experience were analyzed.	($R2 = 0.605$; $p < 0.01$).			
29. Sabine Pleschberger	2007	Cross Sectional	Dignity and the challenge of dying in nursing homes: the residents' view	Background: human dignity is discussed in almost all public debates on the care of the dying, as well as in international policies for health and social care of older people. Because nursing homes are gaining importance as places where	Methods: this qualitative study is based on the Grounded Theory Approach, and the design included three steps of data generation; narrative interviews with residents of nursing homes constitute the main	Results: dignity was differentiated into intrapersonal dignity and relational dignity, socially constructed by the act of recognition. Social relations and encounters are a prerequisite for relational dignity,	Conclusion: the study emphasizes the high vulnerability of nursing home residents with regard to dignity. They place their dignity under the constraints of the need for help and care into question.	-	18

				residents live out their lives in modern western societies and since there is evidence that end-of-life care in nursing homes lacks quality, there is a growing discussion on introducing improved end-of-life care in these institutions. In order to accomplish this, the view of those who are most affected is of utmost importance. Objective: to explore the meaning of dignity with regard to end-of-life issues from the perspective of older nursing home residents in western Germany.	data pool (n = 20) of the results presented in this paper. Theoretical sampling was aimed at maximising the variety of organisational as well as residents' characteristics. Analysis of the transcripts was supported by Atlas/ti program and followed several different coding procedures and aimed at generating a concept of dignity.	which underlines the vulnerability of nursing home residents' who increasingly lack social networks. A broad spectrum of attitudes and behaviour, which aimed at recognising dignity, was bundled under the category 'not being a burden'. In this light, dignity was challenged most by the threat of illness and having care needs. This was fostered by the perception of insufficient care in the nursing homes. In the light of this concept, death with dignity meant 'death at the right time', though the residents in the sample did not want to comment on the time of death, other than aspects like (i) being active to the very last, (ii) respecting one's will and being allowed to die, (iii) not being in pain, (iv) being amongst persons close to one (valediction and showing respect).	This appears alarming, if one does not manage to, with the help of different ethics, obtain a new perspective on these phases of life. It is evident that the understanding of dignity is not solely individualistic and personal, but rather has a close relationship to social ideas of value, which ultimately influence the basic requirements of institutions in which 'frail old people' live.		
30. Harvey Max Chochinov et al.	2004	Editorial	Dignity and Psychotherapeutic	The basic tenets of palliative care are frequently subsumed under the	-	-	-	-	-

			Considerations in End-of-Life Care	goal of helping patients to die with dignity. Our research group has studied the issue of dignity, with dying patients serving as the primary informants. This paper reviews some of our findings, including an overview of the Dignity Model that derives from our empirical work. Furthermore, this paper summarizes various psychotherapeutic approaches which have been considered for this vulnerable patient population. Finally, we provide the rationale based on the Dignity Model for a psychotherapeutic intervention we have coined Dignity Therapy. This brief, individualized therapeutic approach has been informed by our dignity work, and specifically designed for application in patients nearing death.					
31.	Mariska G. Vlug et al.	2011	Cohort Study	The Development of an Instrument To Measure Factors that Influence Self-Perceived Dignity	Background: Preserving dignity can be considered as a goal of palliative care. To provide dignity-conserving	Methods: Data were collected in the Advance Directives Cohort Study. In 2008, the cohort received a	Results: The 31 items fell into four domains: evaluation of self in relation to others, functional status, mental state,	Conclusion: Reducing the instrument to 26 items and dichotomizing the answer option for	16.5	-

				care, it is relevant to identify the factors that influence a patient's self-perceived dignity. This study aims to develop an instrument to measure factors affecting self-perceived dignity that has good content validity and is appropriate for use in practice.	ques- tionnaire with 31 items that might influence one's self-perceived dignity. For a subsample of people with poor health (n 1⁄4 292), we analyzed which items could be removed because of the mean scores for presence of the item and its influence on dignity.	and care and situational aspects. Mean scores for presence and influence on dignity showed large dif- ferences and were not correlated. Six items were scarcely present and did not substantially affect self-perceived dignity. Because three of these were expected to influence dignity in other settings, only three items could be removed and two items could be combined into one. After calculating correlations between conceptually similar items, one extra item could be removed.	presence increases its feasibility for use in practice. The instrument offers an important step to better understanding the phenomenon of self-perceived dignity by gaining information directly from patients.		
32. Aude Lagarriguea et al.	2014	Editorial	Deprivation of liberty and end of life: Does die with dignity mean to die free?	More than 10 years after the establishment of the law of 4 March 2002 on patients' rights and quality of health system, the application of one of its measures, suspension of sentence for medical reasons, remains poor. This arrangement should enable inmates with serious diseases to benefit from a release to be cared for and die free. The low effectiveness of the	-	-	-	-	-

				measure questioned its necessity and sufficiency to meet the goal of a dignified end of life. There are two opposing views regarding its necessity. For proponents of Kantian inherent dignity, detainee's release is not a prerequisite to a dignified end of life. Conversely, it becomes necessary when one adheres to a modern vision of autonomy and 'good death', defined by the expression of their own will. Anyway, caregivers are not responsible for the decision to grant the release, but participate daily to a third way. The terminally ill prisoner is particularly vulnerable. Serious illness affects drastically the identity and lifestyle, individualization factors already weathered by years of detention. Attention and kindness of the caregiver involved in restoring the deteriorated autonomy of the patient in the twilight of his/her life. Only acceptance					

				of our responsibility towards this person, even transgressive, can promote more than the application of the law, the defence of the dignity of the terminally ill prisoner.					
33. Irma Lindstrom et al.	2010	Cross Sectional	Patients' participation in end-of-life care: Relations to different variables as documented in the patients' records	Patients' participation in care is crucial for assuring patients a high quality of care based on values such as autonomy. The patients are supposed to be actively involved in care and treatment, even though these situations are complex, as in the context of end-of-life-care. The aim in this study was to identify demographic and health-related variables' relation to patients' participation during the last three months in life as documented in patients' records.	The population in the present study consists of 229 patients from 49 municipalities in a county in Sweden. Data were collected from all available documentation about deceased patients who were >18 years of age at the time of death and who had received healthcare services during the last 3 months of their life.	This article demonstrates patients' participation in end-of-life care as it was noted in the patients' documentation. Demographic variables such as age, gender, and residence did not differ between those who participated and those who did not. Patients with dementia and disorientation were separated from those who were not disoriented. There was no information about the wishes of the patients with dementia and disorientation and they were not described as participating in care and treatment. Cognitive intact patients were participating significant more often. These patients had also more symptom describes in the records. These	The results of the presents study call attention to the importance of finding innovative solutions to make patients with cognitive dysfunction involved in their care and treatment at end-of-life. Improvement of documentation showing patients' involvement in care is necessary, as is a discussion of how healthcare professionals can assure patients a high quality of care at end-of-life even if patients voices are not heard.	11.5	-

						results can indicate that a patient's participation depends upon either the patient's cognitive capability or the healthcare professionals' competence to communicate and provide adequate documentation regarding patients' participation at end-of-life. The documentation about the participation of patients with cognitive dysfunction is poor and needs further investigation, to achieve the goal of dignified end-of-life care for all patients.			
34. Alison Chapple et al.	2011	Cross Sectional	Patients with pancreatic cancer and relatives talk about preferred place of death and what influenced their preferences: a qualitative study	To explore reasons why people with pancreatic cancer, who are reaching the end of their lives, say they wish to die at home or elsewhere, and why preferences may change.	Qualitative study using semi-structured interviews followed by thematic analysis.<br><br>Respondents recruited from different parts of the UK during 2009/2010.<br><br>16 people with experience of pancreatic cancer (8 patients and 8 bereaved relatives) who discussed place of death in detail during an in-depth interview	People's preferences were affected by their perceptions and previous experiences of care available at home, in a hospice or hospital. Preferences were also shaped by fears about possible loss of dignity, or fears of becoming a burden. Some people thought that a home death might leave bad memories for other members of the family. People with pancreatic cancer and their	The National Health Service End of Life Care Strategy for England seeks to meet the needs of people who are dying and promotes better support for home deaths. More information is needed about why patients hold different views about place of care and place of death, why patients' preferences change and what importance	-	18

					(from a total sample of 32 people with pancreatic cancer and eight relatives of others who had died of this disease).	relatives were aware that preferences might change (or had changed) as death approached.	patients attach to place of death. Health professionals should bear this in mind if the subject is raised during advance care planning.		
35. Edwina M Gerry	2011	Cross Sectional	Privacy and dignity in a hospice environment—the development of a clinical audit	1. A clinical audit was developed and conducted at St Gemma's Hospice, Leeds, to provide evidence of the standard of privacy and dignity afforded to patients in the wards and Day Hospice.<br><br>2. St Gemma's Hospice in the UK provides patient-centred, holistic care, but there had been no formal investigation of privacy and dignity at the hospice. Hence, an audit was conducted to provide evidence of the standard of privacy and dignity afforded to patients.	It involved setting standards from key documents and including patients, carers, and staff in developing questionnaires. The questionnaires were administered to 30 patients and 130 members of the multidisciplinary team.	The response rates were 91% (patients) and 78% (staff). Evaluation of the questionnaires showed that 70% of patients rated their satisfaction with privacy and dignity as 'excellent', with the remaining 30% rating it 'very good'. For the most part, the ratings of staff and patients were in agreement and indicated achievement of the expected standard. However, some areas of concern were identified, including providing opportunity for hand washing prior to meals, closer monitoring of visitor numbers, avoiding interruption to staff, and maintaining privacy during conversations with staff.	Disseminating the results of the audit resulted in some important discussions in the clinical teams and the formulation of an action plan to address the concerns.	-	17
36. Karen H Dening et al.	2013	Cross Sectional	Preferences for end-of-life care: A nominal group study of people with dementia	The wishes and preferences of people with dementia should inform decisions on future care. However, such decisions are	Design: Nominal group technique. Setting/participants : People with dementia (n = 6), carers (n = 5) and	Results: Quality of care, family contact, dignity and respect were ranked as significant themes by all groups. The	Wishes and preferences of people with dementia and their family carers may differ. To	-	15

			and their family carers	often left to family carers and may not reflect those the person with dementia would have made for themselves. We know little about what influences agreement between people with dementia and their carers.<br><br>Aim: To explore whether people with dementia and their carers were able to generate and	dyads of people with dementia and carers (n = 6) attending memory assessment services.<br>Methods: Three modified nominal group technique groups were conducted in five stages: (1) silent generation of ideas, (2) discussion, (3) further generation of ideas, (4) discussion and themeing and (5) ranking. The discussions were recorded, transcribed and analysed for thematic content using NVIVO8.	analysis of transcripts revealed three main themes: quality of care, independence and control and carer burden. People with dementia had difficulty considering their future selves. Carers wanted much control at the end of life, raising issues of assisted dying and euthanasia.	ensure the wishes of people with dementia are respected, their views should be ascertained early in the disease before their ability to consider the future is compromised.		
37. Sue Hall et al	2014	Cross Sectional	Patterns of dignity-related distress at the end of life: A cross-sectional study of patients with advanced cancer and care home residents	Background: To provide effective palliative care in different settings, it is important to understand and identify the sources of dignity related distress experienced by people nearing the end of life.<br><br>Aim: To describe and compare the sources of dignity-related distress reported by cancer patients and care home residents.	Design: Secondary analysis of merged data. Participants completed the Patient Dignity Inventory (assessing 25 sources of dignity-related distress) and measures of quality of life and depression.<br><br>Setting/participants: A total of 45 adult patients with advanced cancer referred to hospital-	Care home residents were older and had poorer functioning. Both groups reported a wide range of dignity-related problems. Although the number or problems reported on the Patient Dignity Inventory was similar for the two groups (mean (standard deviation): 5.9 (5.5) for cancer patients and 4.1 (4.3) for care home residents, p = 0.07),	Although characteristics of the samples differed, similarities in the dignity-related problems reported by cancer patients and care home residents support research suggesting a common pathway towards death for malignant and non-malignant disease. A wider understanding of the sources of	13.5	-

					based palliative care teams in London, United Kingdom, and 60 residents living in one of 15 care homes in London.	there was a tendency for more cancer patients to report some existential problems. Experiencing physically distressing symptoms and functional limitations were prevalent problems for both groups. Patient Dignity Inventory problems were associated with poorer performance status and functioning for residents, with age and cognitive impairment for cancer patients and with poorer quality of life and depression for both groups.	dignity-related distress would help clinicians provide more effective end-of-life care.		
38. Lise J Houmann et al.	2014	Cross Sectional	A prospective evaluation of Dignity Therapy in advanced cancer patients admitted to palliative care	Background: Dignity Therapy is a brief, psychosocial intervention for patients with incurable disease. Aim: To investigate participation in and evaluation of Dignity Therapy and longitudinal changes in patient-rated outcomes.	Design: A prospective (pre/post) evaluation design was employed. Evaluation questionnaires were completed when patients received the generativity document (T1) and 2 weeks later (T2). Changes from baseline (T0) were measured in sense of dignity, Structured Interview for Symptoms and Concerns items,	Results: Over 2 years, 80 of 341 eligible patients completed Dignity Therapy. At T1, 55 patients completed evaluations, of whom 73%–89% found Dignity Therapy helpful, satisfactory and of help to relatives; 47%–56% reported that it heightened their sense of purpose, dignity and will to live. Quality of life decreased (mean = −9 (95% confidence interval: −14.54; −2.49)) and	Conclusions: This study adds to the growing body of evidence supporting Dignity Therapy as a valuable intervention in palliative care; a substantial subset of patients facing end of life found it manageable, relevant and beneficial.	14	-

					Patient Dignity Inventory, Hospital Anxiety and Depression Scale and European Organisation for Research and Treatment of Cancer QLQ-C15-PAL<br><br>Setting/participants : Consecutive patients with incurable cancer, ≥18 years, informed of prognosis and not having cognitive impairment/physica l limitations precluding participation were included at a hospice and a hospital palliative medicine unit.	depression increased (mean = 0.31 (0.06; 0.57)) on one of several depression measures. At T2 (n = 31), sense of dignity (mean = −0.52 (−1.01; −0.02)) and sense of being a burden to others (mean = −0.26 (−0.49; −0.02)) improved. Patients with children and lower performance status, emotional functioning and quality of life were more likely to report benefit.			
39. Parpa E, Kostopoulou S et al.	2017	Cross Sectional	Psychometric properties of the Greek version of the Patient Dignity Inventory in advanced cancer patients	Context. The Patient Dignity Inventory (PDI) is an instrument to measure dignity distressing aspects at the end of life.<br><br>Objectives: The aims of the current study was the translation of the PDI in Greek language as well as to measure its psychometric aspects in a palliative care unit.	Methods: A back-translation method was obtained at the Greek version. 120 advanced cancer patients completed the Greek version of the PDI, the Greek Hospital Anxiety and Depression Scale (Gr-HADS), The Greek Schedule of attitudes towards hastened death (SAHD-Gr) and the Greek SF-12 (Short Form Health Survey: SF-12).	Results: Confirmatory Factor Analysis failed to fit to the original instrument's structure and . EFA was conducted revealing 5 factors ('Psychological Distress', 'Body Image and Role Identity', 'Self-Esteem', 'Physical Distress and Dependency', 'Social Support'). The psychometric analysis of the PDI-Gr demonstrated a good concurrent validity and the	Conclusion. The Greek version of the PDI showed good psychometric properties in advanced cancer patients supported the usefulness of the instrument assessing the sense of dignity distressing aspects of the terminally ill cancer patients.	11.5	-

						instrument discriminated well between subgroups of patients regarding age differences. Cronbach's alphas were between 0.71-0.9 showing a good internal consistency.			
40. Pamela McDermott	2019	Cross Sectional	Patient Dignity Question	While each of the 19 PDQ interviews was unique, there were many consistencies regarding accomplishments (eg, being a good parent), hopes (eg, one's need of being respected), and fears (eg, concerns about pets) that emerged from participants' stories. Hospice staff found the documents from PDQ interviews to be very valuable in their understanding of patients. Staff and patients unanimously wanted the program to continue. An unexpected benefit was the response of the patients' families who were deeply moved by the legacy documents, often sharing them following their family member's death.	Participants completed the Patient Dignity Inventory and modified versions of the Edmonton Symptom Assessment Scale and Integrated Palliative Care Outcome Scale before and after the PDQ interviews.	While each of the 19 PDQ interviews was unique, there were many consistencies regarding accomplishments (eg, being a good parent), hopes (eg, one's need of being respected), and fears (eg, concerns about pets) that emerged from participants' stories. Hospice staff found the documents from PDQ interviews to be very valuable in their understanding of patients. Staff and patients unanimously wanted the program to continue. An unexpected benefit was the response of the patients' families who were deeply moved by the legacy documents, often sharing them following their family member's death.	The PDQ is a dignity-conserving intervention that serves as a meaningful end-of-life legacy document that benefits patients, staff, and families. Using the PDQ at the hospice helped patients feel truly heard, and increased caregivers' compassion and understanding of patients' needs.	-	18

41. Marta Łabuś-Centek	2020	Cross Sectional	The meaning of dignity patient question and changes in the approach to this issue of cancer patients during home hospice care	M.H. Chochinov's dignity question: What do I need to know about you as a person to take the best care of you that I can? is a brief diagnostic and therapeutic intervention. The aim of the study was to assess how cancer patients assess the relevance of the question, how they answer and whether the evaluation of this method changes with the duration of home palliative care.	The study involved 200 patients of the home hospice, who were divided into 2 groups. Group A comprised 100 patients receiving palliative care for up to 7 days, group B included 100 patients under care exceeding 7 days. All patients were posed a dignity question and 2 related ones: whether they consider this question important and whether it should be recommended in practice. In group A, the study was repeated after at least 21 days. Competent judges were then selected and the answers were assigned to specific categories.	The most frequently chosen answer was the one from the category of request for medical staff's<br><br>help or support, which was characterised by the greatest variability under the influence of time — ex-change for an answer: nothing, you already know everything about me. The vast majority of the surveyed<br><br>patients answered affirmatively to the question about the significance of interventions regarding the care for patients and agreed that the question should be recommended in practice.	In most patients the answers to the dignity question change with the duration of home palliative care, which may be related to deepening relations with medical staff. The dignity question has been considered significant regarding the patient care.	10.5	-
42. Vibeke Bruun Lorentsen	2019	Cross Sectional	The meaning of dignity when the patients' bodies are falling apart	People with advanced cancer disease experience great bodily changes due to disease or treatment. They tend to feel ashamed when their bodies are subjected to such changes and they feel their dignity is threatened.	Individual in-depth interviews and participant observations were conducted with 13 patients with advanced cancer disease at a hospice inpatient unit in Norway. Gadamer's ontological hermeneutics inspired the interpretation.	The patients' unpredictable, sick bodies forced the patients, or gave them the opportunity, to relate to their bodies in an honest way. The patients, living in interaction between suffering and health, strove to find dignity. The patients had a will to live and they experienced a	The patients' unpredictable, sick bodies forced the patients, or gave them the opportunity, to relate to their bodies in an honest way. The patients, living in interaction between suffering and health, strove to find dignity. The patients	-	16

						love in their unruly bodies that both helped alleviate their suffering and give them an experience of enhanced dignity. It is important that nurses have insight into the consequences of bodily changes for the patients' experiences of dignity in health and suffering to provide good, dignified care.	had a will to live and they experienced a love in their unruly bodies that both helped alleviate their suffering and give them an experience of enhanced dignity. It is important that nurses have insight into the consequences of bodily changes for the patients' experiences of dignity in health and suffering to provide good, dignified care.		
43. David Rudilla	2016	Cross Sectional	A new measure of home care patients’ dignity at the end of life: The Palliative Patients’ Dignity Scale (PPDS)	This study aimed to develop a new and brief instrument to be employed in dignity measurement, one based on the perceptions of patients, relatives, and professionals about dignity.	Surveys of patients receiving palliative care, family caregivers, and palliative care professionals were first carried out (sample 1). In the second step, palliative care patients were surveyed with a pilot questionnaire (sample 2). Finally, a survey design was used to assess patients admitted into a home care unit (sample 3). Sample 1 included 78 subjects, including patients, family caregivers, and professionals.	After analyzing data from steps 1 and 2, an eight-item questionnaire was presented for validation. The new scale showed appropriate factorial validity (x 2 (19) 1⁄4 21.43, p 1⁄4 0.31, CFI 1⁄4 0.99, GFI 1⁄4 0.92, SRMR 1⁄4 0.07, and RMSEA 1⁄4 0.04), reliability (internal consistency estimations of 0.75 and higher), criterial validity (significant correlations with the hypothesized related variables), and a cut-off criteria of 50 on the overall scale.	The new PPDS has appropriate psychometric properties that, together with its briefness, encourages its applicability for dignity assessment at the end of life.	-	15

					Some 20 additional palliative patients participated in sample 2. Finally, 70 more patients admitted to a home care unit participated were surveyed (sample 3). Together with the Palliative Patients' Dignity Scale (PPDS), our survey included other measures of dignity, anxiety, depression, resilient coping, quality of life, spirituality, and social support.				
44. Deborah L. Volker et al.	2004	Cross sectional	Patient Control and End-of-Life Care Part II: The Patient Perspective	Purpose/Objectives: To explore the nature of what people with advanced cancer want regarding personal control and comfort at the end of life.	Research Approach: Descriptive, naturalistic, using Denzin's model of interpretive interactionism.	A number of cases representative of each trajectory is presented in our results. A fluctuating sense of dignity (dynamic equilibrium) was observed in the majority of participants. Some participants experienced a decline in their sense of dignity as their disease progressed (downward trend), and a few patients exhibited a stable sense of dignity over time (stability).	Conclusions: Participants expressed a wide variety of preferences for personal control and comfort. Their desires reflected personal values and beliefs about how they spend their time and how they want control over their care.	-	18
45. Ilora G. Finlay	2005	Review paper	Quality of life to the end	Quality of life is a subjective concept, yet there have been some real and	-	-	-	-	-

				constructive attempts to measure the quality of a person’s life so that meaningful comparisons can be made during treatment and as disease progresses. In this paper the multifaceted nature of quality of life is explored and the ways that the communication of healthcare professionals with a patient can dramatically influence the patient’s sense of personal dignity and worth. Inherent in this are the relationships with the family, particularly with children in the family, who may suffer greatly in bereavement. Pressures on patients may make them feel a burden, as if they would be better off dead and certainly as if others may be better off without them still alive. This sense of being a burden is often behind requests for death hastening acts such as euthanasia. The fundamental difference between euthanasia and the cessation of futile treatments is also explored. The					

				pivotal role of good communication is the route to ensuring that issues are addressed, with hope maintained for the patient to live as well as possible until they die, and that patients' quality of life is maximized.					
46. L. Balducci	2012	Editorial	Death and dying: what the patient wants	A good death and a death with dignity may be achieved when death is congruent with the personal values of the patient. It behooves the practitioner to recognize these values and to cater to them. This paper describes effective communication with the dying person, and the partnership of treatment team, patient and family in face of the patient death. To identify and define the patient wishes it is necessary to learn how to interpret the patient's non verbal as often patients are unable to formulate the questions they wish to ask concerning their passing. These difficulties stem from several cultural factors including concern about disturbing the practitioner. It is the	-	-	-	-	-

				treatment team's responsibility to facilitate this discussion. A good death is achieved when symptoms are controlled and when patients and family recognize death as a unique living experience to be treasured as any other living experience. A death with dignity brings healing, that is always possible even when cure is out of reach. Patient's and practitioner's values may be at odd in face of controversial issues including euthanasia, assisted suicide and terminal sedation. Though he/she should not be compelled to execute these requests, the practitioner should be able to entertain an open discussion with the patient concerning these issues. Open communication and reflective listening even in presence of disagreements are the venue of healing. The study of death and dying requires novel approaches including personal narrative and qualitative research to complement traditional research					

				instrument, such as questionnaire that cannot embrace the whole human dimension.					
47. Emily A. Meier et al.	2016	Systematic review	Defining a Good Death (Successful Dying): Literature Review and a Call for Research and Public Dialogue	There is little agreement about what constitutes good death or successful dying.	We conducted a literature search for published, English-language, peer-reviewed reports of qualitative and quantitative studies that provided a definition of a good death. Stakeholders in these articles included patients, pre-bereaved and bereaved family members, and health care providers (HCPs). The definitions found were categorized into core themes and sub-themes and the frequency of each theme was determined by stakeholder (patients, family, HCPs) perspectives. Thirty-six studies met our eligibility criteria with 50% of patient perspective articles including individuals over the age of 60 years.	We identified 11 core themes of good death: preferences for a specific dying process, pain-free status, religiosity/spiritualty, emotional well-being, life completion, treatment preferences, dignity, family, quality of life, relationship with HCP, and other. The top three themes across all stakeholder groups were: preferences for dying process (94% of reports), pain-free status (81%), and emotional well-being (64%). However, some discrepancies among the respondent groups were noted in the core themes e.g., family perspective articles included dignity (70%), life completion (80%), and presence of family (70%) more frequently than did patient perspective articles regarding those items (55% for each	While there was agreement among stakeholders in the importance of many aspects of dying well, the presence of discrepancies in certain areas suggests a need for research on the impact of divergent perspectives on end of life care. Dialogues among the stakeholders for each individual must occur to ensure a good death from the most critical viewpoint – the patient's.	-	-

						of these three themes). Religiosity/spirituality was reported more often in patient articles (65%) than in HCP (59%) and family (50%) articles.			
48. Robert M. Lawrence	2007	Editorial	This is the revised text of a lecture presented at the Joint Meeting of the Faculty of Old Age Psychiatry and the Special Interest Group in Spirituality and Psychiatry on 14 December 2005. The paper explores the interface of clinical, ethical, and spiritual dimensions in the promotion of communication in dementia. Themes are derived from personal anecdotal experience, principles of logotherapy, and ethical frameworks. It is argued that individuals with dementia remain at the centre of meaningful interaction through the period of illness and that it is	-	-	-	-	-	-

			incumbent upon formal carers to be aware of the lasting needs of the whole individual, and not to dismiss a spiritual approach. Finally, the paper introduces suggestions for the development of an integrated framework. Strategies and tools are being developed jointly by clinicians and spiritual advisors, and are aimed at preserving the dignity of the person with dementia, their right to be accepted at different stages of decline, in recognition of a meaning to their life and experience even beyond the point where normal language skills are irreparably lost.						
49. Sue Hall et al.	2009	RCT	Assessing the feasibility, acceptability and potential effectiveness of Dignity Therapy for people with advanced cancer referred to a hospital-based	Loss of dignity for people with advanced cancer is associated with high levels of psychological and spiritual distress and the loss of the will to live. Dignity Therapy is a brief psychotherapy,	A randomised controlled open-label trial. Forty patients with advanced cancer are randomly allocated to one of two groups: (i) Intervention (Dignity Therapy	Dignity Therapy is brief, can be delivered at the bedside and may help both patients and their families. This detailed exploratory research shows if it is feasible to offer	-	-	15

			palliative care team: Study protocol	which has been developed to help promote dignity and reduce distress. It comprises a recorded interview, which is transcribed, edited then returned to the patient, who can bequeath it to people of their choosing. Piloting in Canada, Australia and the USA, has suggested that Dignity Therapy is beneficial to people with advanced cancer and their families. The aims of this study are to assess the feasibility, acceptability and potential effectiveness of Dignity Therapy to reduce psychological and spiritual distress in people with advanced cancer who have been referred to hospital-based palliative care teams in the UK, and to pilot the methods for a Phase III RCT.	offered in addition to any standard care), and (ii) Control group (standard care). Recipients of the 'generativity' documents are asked their views on taking part in the study and the therapy. Both quantitative and qualitative outcomes are assessed in face-to-face interviews at baseline and at approximately one and four weeks after the intervention (equivalent in the control group). The primary outcome is patients' sense of dignity (potential effectiveness) assessed by the Patient Dignity Inventory. Secondary outcomes for patients include distress, hopefulness and quality of life. In view of the relatively small sample size, quantitative analyses are mainly descriptive. The qualitative analysis uses the Framework method.	Dignity Therapy to patients with advanced cancer, many of whom are likely to be in the terminal stage of their illness, whether it is acceptable to them and their families, if it is likely to be effective, and determine whether a Phase III RCT is desirable.			

50. Shelley A. Johns	2013	Single group pre-test & post-test	Translating Dignity Therapy Into Practice: Effects and Lessons Learned	Dignity Therapy (DT) is an intervention designed to lessen existential suffering as death draws near. DT has a growing evidence base, with positive outcomes for patients and their family members; however, it is not yet widely available in community settings. The purpose of this project was to translate DT into clinical practice in a cancer centre in the midwestern United States.	DT was provided to 10 patients with metastatic cancer who completed baseline and post-intervention measures of depression, existential distress, health-related quality of life, and satisfaction with the intervention.	DT was found to be feasible and acceptable to the majority of patients and their families.	-	14.5	-
51. Lise-Lotte Franklin et al.	2006	Single group post-test	Views On Dignity Of Elderly Nursing Home Residents	Discussion about a dignified death has almost exclusively been applied to palliative care and people dying of cancer. As populations are getting older in the western world and living with chronic illnesses	-	-	-	-	21

				affecting their everyday lives, it is relevant to broaden the definition of palliative care to include other groups of people. The aim of the study was to explore the views on dignity at the end of life of 12 elderly people living in two nursing homes in Sweden. A hermeneutic approach was used to interpret the material, which was gathered during semi-structured interviews. A total of 39 interviews were transcribed. The analysis revealed three themes: (1) the unrecognizable body; (2) fragility and dependency; and (3) inner strength and a sense of coherence.					
52. Thomas F Hack et al.	2010	Cross-sectional	Learning from dying patients during their final days: life	Dignity therapy is a novel therapeutic approach	One of the products of dignity therapy is a transcript of the	The transcripts revealed that dignity therapy serves to provide a safe,	The findings are discussed in terms of values theory, the role of dignity therapy, and	-	19

			reflections gleaned from dignity therapy	designed to decrease suffering, enhance quality of life and bolster a sense of dignity for patients approaching death. The benefits of dignity therapy were previously documented in a sample of 100 terminally ill patients	edited therapy session(s). In this qualitative study, 50 of the 100 (17 from Winnipeg, Manitoba, Canada, and 33 from Perth, Australia) dignity therapy transcripts were randomly drawn, and independently coded and analysed by three investigators using a grounded theory approach.	therapeutic environment for patients to review the most meaningful aspects of their lives in such a manner that their core values become apparent. The most common values expressed by the patients included 'Family', 'Pleasure', 'Caring', 'A Sense of Accomplishment', 'True Friendship', and 'Rich Experience'. Exemplars of each of these values illustrate the pervasive, defining role of values in our lives	consideration of values clarification in clinicians' efforts to enhance the dignity of terminally ill patients.		
53. Louise Laursen et al	2019	Single group post-test	Table in the corner: a qualitative study of life situation and perspectives of the everyday lives of oesophageal cancer patients in palliative care	Incurable oesophageal cancer patients are often affected by existential distress and deterioration of quality of life. Knowledge about the life situation of this patient group is important to provide relevant palliative care and support. The purpose of this study is to illuminate the ways in which incurable oesophageal	Seventeen patients receiving palliative care for oesophageal cancer were interviewed months after diagnosis. The epistemological approach was inspired by phenomenology and hermeneutics, and the method of data collection, analysis and interpretation consisted of individual qualitative interviews and meaning condensation,	The study reveals how patients with incurable oesophageal cancer experience metaphorically to end up at a "table in the corner". The patients experience loss of dignity, identity and community. The study illuminated how illness and symptoms impact and control daily life and social relations, described under these subheadings: "sense of isolation"; "being in a zombie-like state"; "one day at a time"; and "at sea". Patients feel	The patients' lives are turned upside down, and they experience loss of health, function and familiar, daily habits. The prominent issues for the patients are loneliness and lack of continuity. As far as their normal everyday lives, social networks and the health system are concerned, patients feel they have been banished to a "table in the corner". These patients have a particular need for healthcare professionals who are dedicated to identifying what can be done to support the patients in their everyday lives, preserve dignity and	-	24

				cancer disrupts the patients' lives and how the patients experience and adapt to life with the disease.	inspired by Kvale and Brinkmann.	alone with the threat to their lives and everyday existence; they feel isolated due to the inhibiting symptoms of their illness, anxiety, worry and daily losses and challenges.	provide additional palliative care.		
54. Hai Shan Huang et al.	2018	Single group post-test	The Understanding of Death in Terminally Ill Cancer Patients in China An Initial Study	Patient's needs and rights are the key to delivering state-of-the-art modern nursing care. It is especially challenging to provide proper nursing care for patients who are reaching the end of life (EOL). In Chinese culture nursing practice, the perception and expectations of these EOL patients are not well known.	This article explores the feelings and wishes of 16 terminally ill Chinese cancer patients who are going through the dying process. An open-ended questionnaire with eight items was used to inter- view 16 terminally ill Chinese cancer patients, and was then analyzed by a combined approach employing grounded theory and interpretive phenomenological analysis.	Four dimensions were explored: first, patient's attitudes towards death, such as accepting the fact calmly, striving to survive, and the desire for control; second, the care desired during the dying process, including avoiding excessive treatment and dying with dignity; third, the degree of the patient's acceptance of death; and fourth, the consequences of death.	This cognitive study offers a fundamental understanding of perceptions of death of terminally ill cancer patients from the Chinese culture. Their attitude toward death was complex. They did not prefer aggressive treatment and most of them had given a great deal of thought to their death.	-	23
55. Andrea Rodríguez-Prat et al.	2017	Meta analysis	Understanding patients' experiences of the wish to hasten death: an updated and expanded systematic review and meta-ethnography	Patients with advanced disease sometimes express a wish to hasten death (WTHD). In 2012, we published a systematic review and meta-	The search strategy combined subject terms with free-text searching of PubMed MEDLINE, Web of Science, CINAHL and PsycInfo. The qualitative synthesis followed the methodology	14 studies involving 255 participants with life-threatening illnesses were identified. Five themes emerged from the analysis: suffering (overarching theme), reasons for and meanings and functions of the	-	-	-

				ethnography of qualitative studies examining the experience and meaning of this phenomenon. Since then, new studies eligible for inclusion have been reported, including in Europe, a region not previously featured, and specifically in countries with different legal frameworks for euthanasia and assisted suicide. The aim of the present study was to update our previous review by including new research and to conduct a new analysis of available data on this topic.	described by Noblit and Hare, using the 'adding to and revising the original' model for updating a meta-ethnography, proposed by France et al. Quality assessment was done using the Critical Appraisal Skills Programme checklist.	WTHD and the experience of a timeline towards dying and death. In the context of advanced disease, the WTHD emerges as a reaction to physical, psychological, social and existential suffering, all of which impacts on the patient's sense of self, of dignity and meaning in life.			
56. Harvey Max Chochinov et al.	2005	Cross-sectional	Understanding the Will to Live in Patients Nearing Death	-	-	-	-	12	-
57. Allison Kestenbaum et al.	2017	Single group pre-test & post-test	What Impact Do Chaplains Have? A Pilot Study of Spiritual AIM for Advanced Cancer Patients	Spiritual care is integral to quality palliative care. Although chaplains are	Patients with advanced cancer (n=31) who were receiving outpatient palliative care	From baseline to post-Spiritual AIM, significant increases were found on the FACIT- Sp-12 Faith subscale, the Mini-	Spiritual AIM, a brief chaplain-led intervention, holds potential to address spiritual needs, as well as religious and general	10.5	21

			in Outpatient Palliative Care	uniquely trained to provide spiritual care, studies evaluating chaplains' work in palliative care are scarce. The goals of this pre-post study, conducted among patients with advanced cancer receiving outpatient palliative care, were to evaluate the feasibility and acceptability of chaplain-delivered spiritual care, utilizing the Spiritual Assessment and Intervention Model ("Spiritual AIM"); and to gather pilot data on Spiritual AIM's effects on spiritual well-being, religious and cancer-specific coping, and physical and psychological symptoms.	were assigned based on chaplains' and patients' outpatient schedules, to one of three professional chaplains for three individual Spiritual AIM sessions, conducted over the course of approximately six to eight weeks. Patients completed the following measures at baseline and post-intervention: Edmonton Symptom Assessment Scale (ESAS), Steinhauser spirituality, Brief Religious Coping (Brief RCOPE), Functional Assessment of Chronic Illness Therapy—Spiritual (FACIT-Sp-12), Mini-Mental Adjustment to Cancer (Mini-MAC), Patient Dignity Inventory, Center for Epidemiological Studies – Depression (CES-D, 10-item), and Spielberger State Anxiety Inventory (STAI-S).	MAC Fighting Spirit subscale, and Mini-MAC Adaptive Coping factor. Two trends were observed, i.e., an increase in Positive religious coping and an increase in Fatalism (a subscale of the Mini-MAC).	coping in patients with serious illnesses.		

58. J. Uwimana et al	2008	Cross-sectional	What is the preferred place of care at the end of life for HIV/AIDS patients in countries affected by civil war and genocide: the case of Rwanda?	The main aim of palliative care is to improve the quality of life of people facing threatening diseases by allowing them a dignified death. Therefore, it is crucial to take into account the user's preference of where to be taken care of during the terminal phase of illness. Palliative care in Rwanda is at an early phase of development; therefore, effective planning and implementation that considers the user's view on where to be taken care of is important. This study investigated where HIV/AIDS patients wanted to be cared for in the terminal phase of illness.	-	The results form part of a larger study on met and unmet palliative care needs of people living with HIV/AIDS in selected areas in Rwanda.	-	14	15
59. S. Aoun et al	2008	Single group post-test	A qualitative investigation of the palliative care needs of	1. This paper investigates the support needs of	1. In depth, interviews were conducted with 11 clients from Silver	1. Findings provided useful insights with respect to many of the motivations,	The needs of these individuals are practical, emotional, physical and existential. At the heart of	-	19

			terminally ill people who live alone	people living alone with a terminal illness from a client perspective.<br><br>2. Community and home-based palliative care services are facing increasing challenges in service provision due to an ageing population, an increase in the number of people living alone who require care, a decrease in the availability of family caregivers and a preference to be cared for and die at home	Chain Hospice Care in Western Australia to capture their personal experiences of managing at home alone and to assess their physical, social and emotional needs.<br><br>2. The methodology consisted of face-to-face interviews with clients from both metropolitan and rural settings, to capture their personal experiences of managing at home alone.<br><br>3. In addition to the interviews, participants completed two instruments to assess their functional status at the time of the interview: the Barthel Index and the Australian Modified Karnofsky Performance Scale.	beliefs and wishes of individuals who endeavour to cope on their own with minimal assistance. The needs of these individuals are practical, emotional, physical and existential. At the heart of these concerns is a strong need to be independent and maintain a sense of dignity at end of life.<br><br>2. Results from the client interviews revealed issues in four domains that were important to palliative care clients in the community who were managing at home alone: physical needs, social needs, emotional needs and existential considerations.	these concerns is a strong need to be independent and maintain a sense of dignity at end of life.		
60. Harvey Max Chochinov et al.	2008	Meta analysis	The Patient Dignity Inventory: A Novel Way of Measuring Dignity-Related Distress in Palliative Care	Quality palliative care depends on a deep understanding of distress facing patients	-	-	-	-	-

				nearing death. Yet, many aspects of psychosocial, existential and spiritual distress are often overlooked. The aim of this study was to test a novel psychometric the Patient Dignity Inventory (PDI), designed to measure various sources of dignity-related distress among patients nearing the end of life. Using standard instrument development techniques, this study examined the face validity, internal consistency, test-retest reliability, factor structure and concurrent validity of the PDI. The 25-items of the PDI derive from a model of dignity in the terminally ill.					

				To establish its basic psychometric properties, the PDI was administered to 253 patients receiving palliative care, along with other measures addressing issues identified within the Dignity Model in the Terminally Ill. Cronbach's coefficient alpha for the PDI was 0.93; the test-retest reliability was r ¼ 0.85. Factor analysis resulted in a five-factor solution; factor labels include Symptom Distress, Existential Distress, Dependency, Peace of Mind, and Social Support, accounting for 58% of the overall variance. Evidence for concurrent validity was reported by					

				way of significant associations between PDI factors and concurrent measures of distress. The PDI is a valid and reliable new instrument, which could assist clinicians to routinely detect end-of-life dignity-related distress. Identifying these sources of distress is a critical step toward understanding human suffering and should help clinicians deliver quality, dignity-conserving end-of-life care.					
61. Sue Hall, Harvey Chochinov et al.	2009	RCT	A Phase II randomised controlled trial assessing the feasibility, acceptability and potential effectiveness of Dignity Therapy for older people in care homes: Study protocol	Although most older people living in nursing homes die there, there is a dearth of robust evaluations of interventions to improve their end-of-life care. Residents usually have	A randomised controlled open-label trial. Sixty-four residents of care homes for older people are randomly allocated to one of two groups: (i) Intervention (Dignity Therapy offered in addition to any standard care), and (ii)	This detailed exploratory research shows if it is feasible to offer Dignity Therapy to residents of care homes, whether it is acceptable to them, their families and care home staff, if it is likely to be effective, and determine whether	As recommended by new Medical Research Council guidance, we plan to explore how Dignity Therapy works in this new context, who it works for, and if it fails, why it fails and any unexpected consequences.	-	13

				multiple health problems making them heavily reliant on staff for their care, which can erode their sense of dignity. Dignity Therapy has been developed to help promote dignity and reduce distress. It comprises a recorded interview, which is transcribed, edited then returned to the patient, who can bequeath it to people of their choosing. Piloting has suggested that Dignity Therapy is beneficial to people dying of cancer and their families. The aims of this study are to assess the feasibility, acceptability and potential effectiveness of Dignity Therapy to reduce psychological and spiritual	Control group (standard care). Recipients of the "generativity" documents are asked their views on taking part in the study and the therapy. Both quantitative and qualitative outcomes are assessed in face-to-face interviews at baseline and at approximately one and eight weeks after the intervention (equivalent in the control group). The primary outcome is residents' sense of dignity (potential effectiveness) assessed by the Patient Dignity Inventory. Secondary outcomes for residents include depression, hopefulness and quality of life. In view of the relatively small sample size, quantitative analysis is mainly descriptive. The qualitative analysis uses the Framework method.	a Phase III RCT is desirable.			

				distress in older people reaching the end of life in care homes, and to pilot the methods for a Phase III RCT.					
62. Andrea Rodríguez-Prat et al.	2016	Meta analysis	Patient Perspectives of Dignity, Autonomy and Control at the End of Life: Systematic Review and Meta-Ethnography	Research in the end-of-life context has explored the sense of dignity experienced by patients with advanced disease, examining the factors associated with it. Whereas certain perspectives regard dignity as an intrinsic quality, independent of external factors, in the clinical setting it is generally equated with the person's sense of autonomy and control, and it appears to be related to patients' quality of life. This study aims to explore the relationship between perceived dignity,	We conducted a systematic review and meta-ethnography using reciprocal translation and line-of-argument synthesis. The search strategy used MeSH terms in combination with free-text searching of the PubMed, Web of Science, CINAHL, PsycINFO and Cochrane databases, from their inception until 2015. This identified 186 articles, after excluding duplicates. The inclusion criterion was primary qualitative studies in which dignity, autonomy and control at the end of life were explored. Studies were evaluated using the CASP guidelines.	Twenty-one studies recording the experiences of 400 participants were identified. Three themes emerged: a) dignity mediated by the loss of functionality, linked to the loss of control; b) dignity as identity; and c) autonomy as a determining factor of perceived dignity, understood as the desire for control over the dying process and the desire for self-determination. We propose an explanatory model which highlights that those patients with an intrinsic sense of dignity maintained a positive view of themselves in the face of their illness.	This synthesis illustrates how dignity and autonomy are intertwined and can be perceived as a multidimensional concept, one that is close to the notion of personal identity. The ability to regard dignity as an intrinsic quality has a positive impact on patients, and the design of care strategies should take this into account.	-	-

				autonomy and sense of control in patients at the end of life.					
63. Y. Eskigulek	2019	Cross-sectional	CANCER NURSING: PALLIATIVE AND END-OF-LIFE CARE	Dignity is an important issue for delivering high quality palliative care. This descriptive, methodological study was conducted to evaluate Turkish validity and reliability of The Patient Dignity Inventory (PDI) among palliative care patients and to explore the views of palliative care patients and nurses about dignified care.	This study was approved by Baskent University Institutional Review Board (Project No: KA17/280). Patient demographic form, Palliative Performance Scale and,<br><br>Hospital Anxiety and Depression Scale (HADS) were used for data collection.Ten pal-liative care patients and 10 nurses were interviewed. The semi-structured, face to face<br><br>interviews were recorded digitally, transcribed verbatim and analyzed. Turkish validity and reliability study of the PDI was conducted with 127 palliative care patients with<br><br>advanced cancer. Face validity, factor structure, concurrent validity, internal consistency and	Cronbach's coefficient alpha for the PDI was 0.94 and test-retest reliability was r 1⁄4 0.75. Concurrent validity tests demonstrated positive significant correlations between factors of PDI and HADS. Factor analysis demonstrated 5 factors accounting<br><br>for 68.7% of the overall variance. The factors were labeled as symptom distress, existen-tial distress, self-confidence, dependency and, support and care requirements. Three<br><br>themes emerged through data obtained from palliative care patients: respectability, caring practices, and usefulness. Three themes emerged through data obtained from palliative care nurses: maintaining one's respectability; barriers and	Turkish version of the PDI is a valid and reliable instrument among palliative care patients. Education may be useful for raising awareness of healthcare professionals about dignified care.	-	8

					test-retest reliability analysis were performed.	recommendations; benefits of care.			
64. Aleix Vilalta et al.	2014	Cross-sectional	Evaluation of Spiritual Needs of Patients with Advanced Cancer in a Palliative Care Unit	Spiritual needs play an important role in palliative care as both a clinical dimension and a therapeutic strategy. However, recent studies have shown that the management of this dimension still remains a challenge at the clinical level of palliative care.	An observational study was conducted that involved 50 patients who were recruited between May 2007 and January 2008. A questionnaire was used which included 28 items selected from a review of the literature; the responses were analyzed using a five-point Lickert scale. The results were grouped in 11 categories corresponding to different spiritual needs.	Two spiritual needs emerged as the most relevant for the patients: their need to be recognized as a person until the end of their life and their need to know the truth about their illness. The least important spiritual needs were identified as those: for continuity and an afterlife; to get rid of obsessions; to achieve freedom from blame and to be able to forgive others; and the need for reconciliation and to feel forgiven by others.	When patients knew the truth about their illnesses and they were treated with dignity, their most important needs were likely to be covered. These results suggest that patients receiving palliative care wish to live for the present with as much normality as possible and show only minor concern for their past and future.	13	-

Author	Year Published	Study Design	Title	Intro	Methods	Results	Conclusion	Quality Assessment	
								MERSQI score	COREQ score
65. Daniel Kelly	2009	Cross sectional study	Dying patients with cancer reflected on implications of euthanasia	How do dying patients with cancer talk about making end-of-life medical decisions, including euthanasia?	Patients were interviewed about making end-of-life treatment decisions, starting with questions about do-not-resuscitate decisions. Patients who brought up the	Positive talk. 24 patients spoke positively about euthanasia. Supporters of euthanasia discussed it as a personal choice and viewed opposers as being against individual freedom of	Dying patients with cancer described euthanasia as a personal choice, a compassionate way to end pain and suffering, or a way to preserve dignity in death. Many also noted	-	9

					issue of euthanasia (n = 13) were encouraged to talk about it; those who did not were invited to talk about it. Patients were invited to provide their own definition of euthanasia, and discussions about it ranged from 200 to 2000 words. Interviews concluded with questions about other topics (eg, complementary and alternative medicine and hope). Interviews were audiotaped, transcribed verbatim, coded, and analysed. Analysis focused on how patients talked about euthanasia, used language to justify a position taken, and wider social implications.	choice. However, euthanasia was typically discussed in the abstract context of a hypothetical future or for a hypothetical other person. Euthanasia was considered appropriate for the compassionate ending of extreme pain and suffering. Many patients used the ‘‘dog analogy’’—‘‘you wouldn’t do it to a dog, you’d have it put down’’—to support euthanasia as a medical intervention. The dying process was consistently depicted as inherently distasteful and painful, causing loss of dignity to self and suffering to self and others. Patients concluded that it is better for the individual (and those around him/her) to die than to live with a body that is neither well-	significant moral implications and the potential for abuse of euthanasia in practice.		

						controlled nor dignified. Negative talk. Although many spoke positively about euthanasia, 19 patients expressed concerns and 4 were against it. Patients envisioned difficulties with it as a practice, with a potential for it ‘‘being abused by professionals,’’ practised on those who did not request it, or exploited by patients who might not meet stipulated criteria (referencing a case in recent popular press), further suggesting the need for rigorous guidelines. Some patients confused euthanasia with ‘‘homicide,’’ ‘‘suicide,’’ or ‘‘do not resuscitate’’ orders. Patients noted that those requesting euthanasia might be ‘‘depressed,’’ ‘‘lacking the will			

						to live,'' or ''taking the easy way out.'' They noted vested interests as inevitably at play in the case of complex decisions such as euthanasia and referenced negative moral judgements as a serious potential problem. Some observed that a preference for death could be reversed once pain was brought under control. Others referenced a fine line between decisions made on the basis of adult competence and those made in circumstances of psychological distress. Finally, some opposed euthanasia on the basis that death was not a matter over which individuals ought to exercise choice, believing that life and death were ''God's will.''			
66. Katrine Staats et al.	2020	Cross-sectional study	Dignity and loss of dignity: Experiences of older women living with	In this study, we explored and identified crucial experiences that constitute dignity	In-depth interviews with 13 women, and participant observations of	Crucial experiences that preserved the women's dignity included having	The findings suggest that dignity preservation should be a core	-	18

			incurable cancer at home	and loss of dignity among older women living with incurable cancer at home.	five of these women, were performed. Hermeneutical interpretations of inter- view texts and field notes were conducted	a sense of control, making one's own decisions, experiencing hope and meaningfulness, feeling valued as a human being and having the opportunity to be in a treasured and nurturing environment. Dignity loss was related to losing the opportunity for self-determination, sensing hopelessness and worthlessness in a shroud of illness, experiencing violation of their personal life and being situated in surroundings that enhanced their sense of disconnection and alienation. Quality of care was experienced as more important than the physical place in which to spend their final stage of life.	dimension in care for older women living with incurable cancer at home. Future research should investigate how dignity-preserving care can be organized and practiced within municipal palliative care services.		
67. Malene Missel et al.	2020	Cross sectional study	The Understanding of Dignity Among In-Hospital Patients Living With	Dignity is an inherent value in palliative care, but understanding dignity among	A qualitative hermeneutic approach, inspired by Gadamer, guided the	The meaning of dignity was revealed as reverential response in care relationships	A dialogical and understanding approach is significant in making the patient feel	-	16

			Incurable Esophageal Cancer	people living with and hospitalized for incurable esophageal cancer has not been explored. The aim of this study was to empirically explore the meaning of dignity in people hospitalized with incurable esophageal cancer.	research process and interpretation of the transcribed interviews. Eighteen patients participated in the study.	and eating as an undignifying activity. A balance of the healthcare system's framework with the lifeworld of the patient was significant in preserving dignity and gave patients a sense of reverent response. Patients were unable to eat ordinary daily meals, which affected their perception of own body and identity, including interactions with others. The resulting bodily changes and social consequences were of crucial importance to the perceived dignity.	worthy, consequently helping to uphold a sense of dignity. The changing and decaying body due to eating difficulties bears witness to illness, altering the individual's sense of self, in turn threatening the dignity of identity.		
68. Jack Coulehan	2005	Case study	"They wouldn't pay attention": Death without dignity	"Death with dignity" is a common catchphrase, especially in conversations about medical intervention near the end of life. Stripped to a sound bite, it may be used as a rallying cry	-	-	The case of Joyce Evans exemplifies a common problem in American end-of-life care. On the surface, it appeared as if she was fully informed and had control	-	5

				for physician-assisted suicide and euthanasia or a condemnation of the American way of dying. While the phrase conveys deep beliefs about how persons face—or ought to face—the final phase of life, these beliefs are often not clearly formulated. Since death is a subject we like to avoid, we find it comfortable to use code in speaking about it. “Death with dignity” can serve as one such code. Unfortunately, patients, families, and healthcare professionals may use the code in conversations without realizing that each person involved interprets it differently. In this essay, I explore some of the possible meanings of “death with dignity.”			of the situation. The processes of informed consent ensured her dignity as a rational decision maker. Nonetheless, she and her family claimed that she died without dignity because her healthcare professionals didn’t respect or pay attention to her. From the dignity as choice perspective, she was clearly complicit, because her choices continually undermined her avowed goals.		

69. Mariska G Oosterveld-Vlug et al.	2008	Cohort	Assessing the validity and intra-observer agreement of the MIDAM-LTC; an instrument measuring factors that influence personal dignity in long-term care facilities	Patients who are cared for in long-term care facilities are vulnerable to lose personal dignity. An instrument measuring factors that influence dignity can be used to better target dignity-conserving care to an individual patient, but no such instrument is yet available for the long-term care setting. The aim of this study was to create the Measurement Instrument for Dignity AMsterdam - for Long-Term Care facilities (MIDAM-LTC) and to assess its validity and intra-observer agreement.	Thirteen items specific for the LTC setting were added to the earlier developed, more general MIDAM. The MIDAM-LTC consisted of 39 symptoms or experiences for which presence as well as influence on dignity were asked, and a single item score for overall personal dignity. Questionnaires containing the MIDAM-LTC were administered face-to-face at two moments (with a 1-week interval) to 95 nursing home residents residing on general medical wards of six nursing homes in the Netherlands. Constructs related to dignity (WHO Well-Being Five Index, quality of life and physical health status) were also measured. Ten residents answered the questions while	Nine of the 39 items barely exerted influence on dignity. Eight of them could be omitted from the MIDAM-LTC, because the thinking aloud method revealed sensible explanations for their small influence on dignity. Residents reported that they missed no important items. Hypotheses to support construct validity, about the strength of correlations between on the one hand personal dignity and on the other hand well-being, quality of life or physical health status, were confirmed. On average, 83% of the scores given for each item's influence on dignity were practically consistent over 1 week, and more than 80% of the residents gave consistent scores for the single item score	The MIDAM-LTC has good content validity, construct validity and intra-observer agreement. By omitting 8 items from the instrument, a good balance between comprehensiveness and feasibility is realised. The MIDAM-LTC allows researchers to examine the concept of dignity more closely in the LTC setting, and can assist caregivers in providing dignity-conserving care.	10	15

					thinking aloud. Content validity, construct validity and intra-observer agreement were examined.	for overall dignity.			
70. Linda Mah et al.	2013	Cross sectional	Association between patient dignity and anxiety in geriatric palliative care	Preservation of patient dignity is increasingly an important focus in end-of-life care and research. However, the experience of terminally-ill older adults has rarely been systematically examined. We previously reported results of a pilot study using Chochinov's Patient Dignity Inventory1 in a Geriatric Palliative Care Unit (PCU).2 While greater dignity-related distress was experienced amongst geriatric PCU patients, the specific PDI items identified as concerns were remarkably consistent with studies of cancer patients across the adult lifespan in community and	Twenty-seven patients admitted to the PCU in a tertiary care geriatric hospital were studied (14 males, mean age = 80 years (SD = 9.8), mean Palliative Performance Scale score = 49.2% (SD 9.3)). Primary admitting diagnoses were cancer (n = 23), congestive heart failure (n = 3) and beta-thalassemia disease (n = 1). Mean duration of survival was 106 days (SD = 72.9) from the time of testing, with 25 patients deceased by the study end. All patients completed the Edmonton Symptom Assessment Scale (ESAS),5 a standard 10-item symptom screening tool used in palliative	Correlational analyses using Pearson's r were per- formed to determine associations between clinical symptomatology and dignity-related distress using total PDI and subscale scores (Symptom Distress, Existential Distress, Dependency, Peace of Mind, and Social Support). A Bonferroni correction was applied to interpret findings from correlational analyses of specific PDI subscales and clinical measures (results reported for p < .0025, two- tailed). SPSS version 20.0 (IBM Corporation, Armonk, NY) was used for the analyses. Mean	Our preliminary findings suggest that dignity-related distress and anxiety are highly prevalent in geriatric palliative care, and that dignity concerns are associated with greater distress as manifested by anxiety symptoms, in contrast to previous reports1. Anxiety is a frequent indication for palliative sedation and a well-recognized clinical problem in terminally-ill patients.10,11,12 Yet, there is a paucity of research aetiologies and treatment of anxiety in palliative care settings.12 A comprehensive evidence-based guideline for screening and treatment of depression in European	6	-

				hospital settings.3,4 We extend our previous findings with a report on associations amongst dignity-related concerns and clinical severity of mood, anxiety, and physical symptomatology .	care settings with subscales for psychological (ESAS-Psy) and physical symptoms (ESAS-Phy); Hospital Anxiety and Depression Scale (HADS),6 a well-validated measure of severity of anxiety and depression symptoms in medically-ill populations7 and the Patient Dignity Inventory (PDI).1 The PDI is a 25-item self-report questionnaire which assesses the degree to which terminally-ill patients experience concerns relevant to personal dignity. Items cluster within five domains: Symptom Distress, Existential Distress, Dependency, Peace of Mind, and Social Support.1 The PDI has been implemented in other clinical settings.8,9	total score on the PDI was 51.7 (SD = 22.5). One-third of the PDI items were rated by patients as being problematic, falling primarily within Dependency (42%), Symptom Distress (38.9%), and Existential Distress (34.6%) domains (see table in the study by Mah et al.2 for mean item ratings). Approximately one-fifth of the patients reported moderate to severe anxiety and depressive symptomatology (HADS score > 11; HADS-A: 19%; HADS-D: 22%). In all, 15% had mild anxiety and 30% had mild depressive symptoms (score: 8–10). Mean ESAS total score was 29.7 (SD 12.6) with mean ESAS-Psy score of 9.4 (SD 6.7) and mean ESAS-Phy score of 20.2 (SD 9.3).	palliative cancer settings has become available,13 but practice guidelines do not yet exist for management of anxiety in end-of-life care. Our findings of an association between anxiety and dignity-related distress suggest a need for dignity-conserving care in particular. One model of care is the Dignity Care Pathway (DCP), an individualized approach to understanding and managing concerns related to dignity.14,15 Future research focused on evaluating the impact of approaches such as the DCP on anxiety severity would guide management of psychosocial distress in palliative care.		

						Individual patients' PDI total scores were strongly correlated with their self-report of anxiety and depression severity (HADS-A: $r = .70$, $p < .001$; HADS-D: $r = .58, p = .002$; ESAS-Psy: $r = .61$, $p = .001$), and moderately with severity of physical symptoms (ESAS-Phy: $r = .45$, $p = .018$). Correlational analyses using specific PDI sub-scales showed significant associations between anxiety severity as assessed using HADS-A and the domains Symptom Distress, Existential Distress, Peace of Mind, and Social Support ($r = .61–.72$; $p \leq .001$), and no associations with Dependency. Similarly, anxiety severity as assessed using ESAS was significantly correlated with			

						PDI subscales of Symptom Distress, Existential Distress, and Peace of Mind (r = .54–.58, p ≤ .003). However, ESAS anxiety was not correlated with either Dependency or Social Support (r = .38, p = .054). In contrast to the associations between anxiety severity and multiple PDI domains, depressive symptomatology was correlated with Peace of Mind concerns only (HADS-D: r = .59, p = .001; ESAS depression, not significant). The Dependency sub-scale correlated only with severity of physical symptoms (ESAS-Phy: r = .60, p = .001).			
71. Linda Mah et al.	2012	Cross sectional	ASSESSMENT OF PATIENT DIGNITY IN GERIATRIC PALLIATIVE CARE	End-of-life care over the last decade has increasingly focused on preservation of dignity to improve quality of life and	The PDI was administered to 27 older adults (14 men, mean age 80.4 ± 9.8, mean Palliative Performance Scale score = 49.2 ± 9.3%)	Participants reported an average of 7.8 ± 7.5 problems on the PDI. Mean ratings of each item on the PDI, the proportion of	Contrary to previous investigations of dignity in terminally ill individuals in hospital and community settings,3,4 this	6	-

				reduce distress, but the prevalence of dignity-related distress in individuals receiving palliative care has received little research attention. It has been reported that fewer than 10% of individuals with cancer rated loss of dignity a problem.1 Similarly, studies using the Patient Dignity Inventory (PDI)2 suggest that individuals with cancer receiving palliative care in the community or hospital experience little dignity-related distress.3,4 Whether these findings are applicable in the setting of an inpatient geriatric palliative care unit is unknown.	admitted to an inpatient palliative care unit in a tertiary care geriatric hospital. Primary admitting diagnoses were cancer (n = 23), congestive heart failure (n = 3), and beta-thalassemia disease (n = 1). Twenty-five individuals had died by study end (mean duration of survival 106 ± 72.9 days from time of testing). The PDI is a 25-item self-report questionnaire that assesses the degree to which terminally ill individuals have concerns relevant to personal dignity (Table 1). The scale has been demonstrated to have high internal consistency and test–retest reliability. Factor analysis of the PDI identified five components: symptom distress, existential distress,	individuals who rated the item as being a problem (rating 3), and means for each subscale on the PDI are summarized in Table 1. Items on the PDI associated with dependency (42%) and symptom distress (39%) were most frequently identified as significant problems, followed by existential distress (35%) and peace-of-mind concerns (25%). Few identified social support as a problem. Mean PDI total scores significantly correlated with anxiety and depression (HADS anxiety; correlation coefficient r = 0.69, P < .001; HADS depression; r = 0.56, P = .003; ESAS-Psy; r = 0.62, P = .001) and physical symptoms (ESAS-Phy; r = 0.45, P = .01).	pilot study suggests that dignity-related distress is highly prevalent in geriatric palliative care inpatients, although the specific dignity-relevant concerns identified were remarkably consistent with those found in previous studies.3,4 In all three studies, the five most problematic issues that individuals receiving palliative care experienced were physically distressing symptoms, followed by “no longer feeling like who I was,” “not able to carry out important roles,” “not able to perform tasks of daily living,” and “not able to continue usual routines.” The five least problematic in all three studies were “not feeling supported by healthcare providers,” “not feeling		

					dependency, peace of mind, and social support. The PDI has been used to study dignity- related distress in residents of nursing homes5 and in individuals with advance directives.6 Participants also completed the Hospital Anxiety and Depression Scale (HADS), measure with high reliability and validity in a broad range of medically ill populations, including palliative care settings,7 and the Edmonton Symptom Assessment Scale (ESAS),8 a standard 10-item symptom screening tool used in palliative care settings to rate physical (ESAS-Phy; pain, activity, nausea, drowsiness, appetite, dyspnea) and psycho- logical (ESAS-Psy; depression, anxiety, well-		supported by friends or family," "not being treated with respect," "concerns about spiritual life," and "not being able to accept things as they are." The consistency in frequency of problems endorsed on the PDI across studies is striking and surprising, given the differences in sample size, clinical settings, primary diagnoses, and age range. These similarities suggest the feasibility of developing standardized mental health protocols as part of palliative care, similar to the pain or sedation protocols used in hospice or inpatient palliative care settings. A number of psychotherapies have been developed to address end-of-life concerns in terminally ill		

					being items) symptoms. The Baycrest research ethics board approved this study. All participants provided written informed consent before study entry.		individuals, but their efficacy and applicability in a range of palliative care settings have yet to be established (see review9). Determining the prevalence of specific dignity-related concerns may help identify common core themes to target in developing psychotherapeutic interventions for palliative care. Furthermore, given that the controversial use of palliative sedation in managing existential distress is, in part, due to a poorly defined construct,10 objective measures of psychological suffering such as the PDI may help establish clearer guidelines for the use of palliative sedation for psychological symptoms in terminally ill individuals.		

72. Anne Hughes et al.	2008	Focus group	‘‘Can You Give Me Respect?’’ Experiences of the Urban Poor on a Dedicated AIDS Nursing Home Unit	In this interpretive phenomenology study, 10 impoverished adults with advanced HIV disease, ages 35 to 58 years, described their understanding of dignity and their everyday experiences in an urban AIDS-dedicated nursing home unit. Three group interviews, along with field notes, were audiotaped, transcribed, and analyzed. For most, dignity meant respect received by others and respect for oneself; some did not understand the word dignity. Receiving respectful care enhanced a sense of dignity for some, and for others, difficulties with caregivers led to feeling unrecognized or disrespected. Everyday experiences of living on the unit	-	-	-	-	18

				included narratives about how they got there, taking antiretrovirals, escaping, the unit as a community of caring, witnessing deaths, relationships with nurses, the unit as a place of safety, and knowing when to leave. This study contributes to knowledge of marginalized minority groups with advanced HIV disease whose experiences often remain silenced.					
73. Thomas F. Hack et al.	2018	Cross sectional	Assessing Symptoms, Concerns, and Quality of Life in Noncancer Patients at End of Life: How Concordant Are Patients and Family Proxy Members?	It has become commonplace to use family caregivers as proxy responders where patients are unable to provide information about their symptoms and concerns to health care providers.	Sample dyads included a mix of patients residing at home, in a nursing home, in a long-term care facility, or in hospice. Diagnoses included patients with amyotrophic lateral sclerosis (n = 75), chronic obstructive pulmonary disease (n = 52), end-stage renal disease (n = 42), and institutionalized,	Concordance was less than 70% for seven of the 25 PDI items, with the lowest concordance (65.1%) for the item "not being able to continue with my usual routines." For all but one PDI item, discordance was in the direction of family members reporting that the patient was worse off than	Understanding discordance between patients and family member reports of symptoms and concerns is a valuable step toward minimizing patient and family burden at end of life.	9.5	-

					cognitively intact frail elderly (n = 49). Dyads completed the Patient Dignity Inventory (PDI), the modified Structured Interview Assessment of Symptoms and Concerns in Palliative Care, and Graham and Longman's two-item Quality of Life Scale.	the patient had indicated. Where discordance was observed on the Structured Interview Assessment of Symptoms and Concerns in Palliative Care and Quality of Life Scales, the trend toward family members overreporting patient distress and poor quality of life continued.			
74. Dennis D. Waskul et al	2002	Cross sectional	The Abject Embodiment of Cancer Patients: Dignity, Selfhood, and the Grotesque Body	The body is the empirical quintessence of the self. Because selfhood is symbolic, embodiment represents the personification and materialization of otherwise invisible qualities of personhood. The body and experiences of embodiment are central to our sense of being, who we think we are, and what others attribute to us. What happens, then, when one's body is humiliating? How does the self-handle the implications of a	-	-	-	-	16

				gruesome body? How do people manage selfhood in light of grotesque physical appearances? This study explores these questions in the experiences of dying cancer patients and seeks to better understand relationships among body, self, and situated social interaction.					
75. Ulrika Östlund et al.	2019	Cross sectional and focus group	How to conserve dignity in palliative care: suggestions from older patients, significant others, and healthcare professionals in Swedish municipal care	An essential aspect of palliative care nursing is to conserve the dignity of the patient. A Dignity Care Intervention (DCI) has been developed in Scotland to facilitate this role for nurses. The DCI is now being adapted to a Swedish context (DCI-SWE) and a central step is to identify culturally relevant, dignity-conserving care actions. These care actions will be incorporated into the DCI-SWE. Therefore,	This study used a descriptive design with a qualitative approach. Data from 20 participants were collected through semi-structured individual interviews with patients (n = 3), SOs (n = 4), two focus groups with nurses (n = 9) and one focus group with physicians (n = 4) in two Swedish municipalities. These data were deductively analysed using qualitative content analysis with the	With the Chochinov model of dignity as a framework, care actions based on suggestions from the participants were identified and presented under three themes: Illness related concerns, Dignity conserving repertoire, and Social dignity inventory. The study found both specific concrete care actions and more general approaches. Such general approaches were found to be	As part of the adaption of the DCI from a Scottish to a Swedish context, this study added relevant care actions for collaborative planning of individualised care in mutual dialogues between nurses and those they care for. The adapted intervention, DCI-SWE, has the potential to help the nurses in providing palliative care of evidence-based quality.	-	13

				the aim of this study was to suggest care actions for conserving dignity in palliative care from the perspectives of the patients, significant others (SOs), and health care professionals (HPs) in municipality care in Sweden.	Chochinov model of dignity as framework.	relevant for several dignity related issues as all-embracing attitudes and behaviours. However, these general approaches could also be relevant as specific care actions to conserve dignity in relation to certain issues. Care actions were also found to be linked to each other, showing the importance of a holistic perspective in conserving dignity.			
76. Iris Crespo et al.	2020	Cross sectional	Health-related quality of life in patients with advanced cancer who express a wish to hasten death: A comparative study	Some evidence suggests the wish to hasten death is related to poor health-related quality of life. Deficits in perceived dignity and self-efficacy are risk factors for wish to hasten death that also impact health-related quality of life. This study seeks to compare perceived health-related quality of life, dignity and self-efficacy in	A total of 153 adult patients with advanced cancer were assessed for wish to hasten death using the Desire for Death Rating Scale. Scores ⩾1 indicate some degree of wish to hasten death (case group, n = 51), and score = 0 implies no wish to hasten death (control group, n = 102). Assessments included health-related quality of	Patients with a wish to hasten death had worse emotional functioning ($p < 0.001$), greater perceived loss of dignity ($p < 0.001$) and lower self-efficacy ($p = 0.001$). There was no difference in most physical symptoms. Perceived overall health-related quality of life was significantly worse for those with a clinically	Patients with wish to hasten death showed lower perceived dignity, self-efficacy and emotional quality of life than patients without wish to hasten death without necessarily perceiving worse physical symptoms.	7	-

				patients with advanced cancer who either do (case group) or do not (control group) express a wish to hasten death. Cases and controls were matched on sociodemographic and functional characteristics.	life using the European Organization for Research and Treatment of Cancer Quality-of-Life Core 15-Item Palliative Questionnaire, perceived loss of dignity using the Patient Dignity Inventory and self-efficacy using the General Self-Efficacy Scale.	relevant wish to hasten death (p = 0.023) and marginally worse for the case group than the control group (p = 0.052).			
77. David M. Clarke	2007	Grey lit	Growing old and getting sick: Maintaining a positive spirit at the end of life	End of life throws up significant mental health challenges. A high proportion of people in the terminal stages of illness experience depressive symptoms. This paper integrates a theory of hierarchy of human needs and empirical research describing experiences of grief and depression in terminal illness, to develop a model of care aimed at reducing depression and suffering. This	-	-	-	-	-

				care attends to physical, psychological, social and spiritual aspects, taking into account the concerns of patients and their families. Professional help can be offered to patients to restore dignity and hope, strengthen their ways of coping, and encourage social connections. To offer this, a well-resourced and coordinated, multidisciplinary and skilled workforce is needed.					
78. Doris Leung	2007	Systematic review	Granting death with dignity: patient, family and professional perspectives	Dignity is a complex construct lacking clear meaning. While conceptualising dignity as a basic right is useful in determining and justifying social and economic costs of health care, it is insufficient in considerations of personal dignity at the	-	-	-	-	-

				end of life. There is a dissonance between how dignity is shown to matter to healthcare professionals compared to patients. Furthermore, dignity is not clearly linked in the empirical literature to variables of quality of life and to a dignified death. Current studies about the construct of dignity enhance understanding of how we extrinsically construct moral worth, but not of how individuals interpret intrinsic moral worth through maintaining their personal integrity and attitudes of being cared for. References to key qualitative studies illuminate how clinicians ethically negotiate a creation of dying with dignity. As one’s					

				personal integrity fades, caregivers (i.e. healthcare providers, family and friends) are challenged to recognise and attend to the individual's vulnerability. I suggest that caregivers nurture personal integrity – through gestures that remember and honour aspects of the other as he/she was once known. Perhaps only through others can dying people be granted death with a sense of personal dignity					
79. Ross Fewing	2014	Grey lit	A Fading Decision	An advance directive and a durable power of attorney for health care, supported by good ongoing family communication, can make the decisions for end-of-life care much easier to navigate.	-	-	-	-	-
80. Li-Shan Ke et al.	2016	Systematic review	Experiences and perspectives of older people regarding	Studies have indicated that family members or health	Design: A systematic review of qualitative	A total of 50 articles were critically appraised and a	Older people's perspectives and experiences of advance care	-	-

			advance care planning: A meta-synthesis of qualitative studies	professionals may not know or predict their older relatives' or patients' health preferences. Although advance care planning is encouraged for older people to prepare end-of-life care, it is still challenging.	studies and meta-synthesis was conducted. Data sources: CINAHL, MEDLINE, EMBASE, and PsycINFO databases were searched.	thematic synthesis was undertaken. Four themes were identified: life versus death, internal versus external, benefits versus burdens, and controlling versus being controlled. The view of life and death influenced older people's willingness to discuss their future. The characteristics, experiences, health status, family relationship, and available resources also affected their plans of advance care planning. Older people needed to balance the benefits and burdens of advance care planning, and then judge their own ability to make decisions about end-of-life care.	planning were varied and often conflicted; cultural differences amplified variances among older people. Truthful information, available resources, and family support are needed to enable older people to maintain dignity at the end of life. The views of life and death for older people from different cultures should be compared to assist health professionals to understand older people's attitudes toward advance care planning, and thus to develop appropriate strategies to promote advance care planning in different cultures.		

Author	Year Published	Study Design	Title	Intro	Methods	Results	Conclusion	Quality Assessment	
								MERSQI score	COREQ score

81. Ana Isabel García Pérez et al.	2014	Case Report	Case Report Of A Computer-Assisted Psychotherapy Of A Patient With Als	This case describes a psychotherapy intervention in a patient in advanced stages of ALS. The inability for verbal communication at these stages necessitated the inclusion of a computational system to favor augmentative and alternative communication (AAC) to provide psychological care. The association of this device and software with ongoing psychotherapy acted in a synergistic manner. AAC devices made it possible to maintain patient-therapist communication and provided material support for psychotherapy despite severe speech limitations. This bimodal protocol of intervention resulted in better symptom control, improved communication with the team and family,	-	-	-	-	9

				reduction of psychological distress, promotion of autonomy, dignity, and self-esteem. The novelty of this communication is to report how the regular psychological care could be adapted to the patient circumstances using a computer device. Clinical trials will be required to evaluate the effectiveness of this mode of psychotherapy for the general population of ALS patients.					
82. Lucille Marchand et al.	2016	Randomized Control Trial	Existential Suffering in Advanced Cancer: The Buffering Effects of Narrative	Dignity-enhancing therapy is a narrative life review intervention shown to improve well-being in hospice and palliative care patients. Our previous pilot study using a modified dignity-enhancing, life-story intervention suggested that pre-hospice, advanced cancer patients could benefit from this intervention. This randomized	117 patients with stage 3 or 4 cancer were recruited from the University of Wisconsin Carbone Cancer Center and randomized 1:1 to My Living Story or to an active control group with access to accurate online cancer resources. Existential wellbeing was assessed with the FACIT-Sp subscales for	At four months, the intervention group had a significantly increased greater sense of peace (p¼ .029) than the active control group. The intervention group had higher scores for existential wellbeing (p¼.096) and lower scores for depression (p¼.102) than the control group, whose scores	My Living Story, a modified Dignity-Enhancing Story program, improved advanced cancer patients' sense of peace and buffered against the worsening depression and existential wellbeing seen in the online cancer information control group.	11	-

				control trial was designed to test the effects of My Living Story on existential wellbeing and psychological distress in advanced cancer patients. My Living Story included a life review interview, the delivery of the life story, and online cancer information and personalized tools to revise and share the story with loved ones.	peace and wellbeing, and the POMS-SF subscales for anger, depression, anxiety at baseline, 2 and 4 months after intervention. Linear Mixed Model, controlling for pre-test, tested for group comparisons of repeated outcome measures and their subscales at 2 and 4 months.	worsened while the intervention group maintained or slightly improved.			
83. Sylvia Patricia Duarte	2003	Cross-Sectional Study	An exploration of dignity in palliative care	This paper presents a qualitative study exploring the meaning of ’dignity‘ to patients, relatives and professionals. It examines the impact of advanced illness and treatment and the issues pertinent to caring for dignity.	Depth interviews were conducted with eight patients, six relatives and seven members of the multi-professional team. A phenomenological approach to data analysis was adopted.	Dignity was found to be a complex phenomenon. It is composed of the dimensions ’bein g human‘, ’having control‘, ’relation ship and belonging‘ and ’ maintaining the individual self‘. These dimensions seemed to be held in equilibrium by each individual. The importance of each may alter in response to threats to dignity such as advancing	Caring for dignity is challenging because it involves balancing the multiple needs of both users and providers of palliative care and the different perceptions and dimensions of dignity itself.	-	16

						illness and how one is treated within it, in an attempt to cope and adapt.			
84. Andrea Bovero et al.	2019	Cross-Sectional Study	Exploring demoralization in end-of-life cancer patients: Prevalence, latent dimensions, and associations with other psychosocial variables	Demoralization is an existential distress syndrome that consists of an incapacity of coping, helplessness, hopelessness, loss of meaning and purpose, and impaired self-esteem. It can affect cancer patients, and the Demoralization Scale is a valid instrument to assess it. The present study aimed to investigate the prevalence of demoralization in end-of-life cancer patients and its associations with the medical and psychosocial variables. In addition, the latent dimensions of demoralization emerging in this distinctive population were explored.	The study is cross-sectional. The sample consisted of 235 end-of-life cancer patients with a Karnofsky performance status (KPS) lower than 50 and a life expectancy of a few weeks. For each patient, personal and medical data was gathered by a palliative physician and a set of validated rating scales, assessing demoralization, anxiety, depression, physical symptoms, pain, spiritual well-being, and dignity, was administered by a psychologist during the first consultation.	Sixty-four participants (27.2%) had low demoralization, 50.2% (n = 118) had medium demoralization, and 22.6% (n = 53) had high demoralization. Factor analysis evidenced a five- factor solution that identified the following demoralization factors: Emotional Distress and Inability to Cope, Loss of Purpose and Meaning, Worthlessness, Sense of Failure, and Dysphoria. All the considered variables were associated with demoralization, except for pain, nausea, breathing problems, and sociodemographic and clinical variables.	End-of-life cancer patients showed higher levels of demoralization than has been reported in other studies with advanced cancer. These data could suggest that demoralization could increase in proximity to death and with impaired clinical condition. In particular, the five demoralization dimensions that emerged could represent the typical concerns around which the syndrome evolves in end-of-life cancer patients. Finally, spiritual well-being could play a protective role with respect to demoralization.	12	-
85. Muhammad M. Hammami et al.	2015	Cross-Sectional Study	Exploring end of life priorities in Saudi males: usefulness of Q-methodology	Quality end-of-life care depends on understanding patients' end-of-life choices.	We explored Saudi males' forced-ranked, end-of-life priorities and	Respondents' mean age was 32.1 years (range, 18–65); 52 % reported	1) Transcendence was the extreme end-of-life priority, and	12	-

				Individuals and cultures may hold end-of-life priorities at different hierarchy. Forced ranking rather than independent rating, and by-person factor analysis rather than averaging may reveal otherwise masked typologies.	dis-priorities. Respondents (n = 120) rank-ordered 47 opinion statements on end-of-life care following a 9-category symmetrical distribution. Statements' scores were analyzed by averaging analysis and factor analysis (Q-methodology).	average religiosity, 88 and 83 % ≥ very good health and life-quality, respectively, and 100 % ≥ high school education. Averaging analysis revealed that the extreme five end-of-life priorities were to, be at peace with God, be able to say the statement of faith, maintain dignity, resolve conflicts, and have religious death rituals respected, respectively. The extreme five dis-priorities were to, die in the hospital, not receive intensive care if in coma, die at peak of life, be informed about impending death by family/friends rather than doctor, and keep medical status confidential from family/friends, respectively. Q-methodology classified 67 % of respondents into five highly	dying in the hospital was the extreme dis-priority. 2) Quality of life was conceptualized differently with less emphasize on its physiological aspects. 3) Disclosure of terminal illness to family/close friends was preferred as long it is through the patient. 4) Q-methodology identified five types of constellations of end-of-life priorities and dis-priorities that may be related to respondents' demographics and are partially masked by averaging analysis.		

						transcendent opinion types. Type-I (rituals-averse, family-caring, monitoring-coping, life-quality-concerned) and Type-V (rituals-apt, family-centered, neutral-coping, life-quantity-concerned) reported the lowest and highest religiosity, respectively. Type-II (rituals-apt, family-dependent, monitoring-coping, life-quantity-concerned) and Type-III (rituals-silent, self/family-neutral, avoidance-coping, life-quality & quantity-concerned) reported the best and worst life-quality, respectively. Type-I respondents were the oldest with the lowest general health, in contrast to Type-IV (rituals-apt, self-			

						centered, monitoring-coping, life-quality/quantity-neutral). Of the extreme 14 priorities/dis-priorities for the five types, 29, 14, 14, 50, and 36 %, respectively, were not among the extreme 20 priorities/dis-priorities identified by averaging analysis for the entire cohort.			
86. Geok Ling Lee et al.	2013	Cross-Sectional Study	Exploring the experience of dignified palliative care in patients with advanced cancer and families: A feasibility study in Singapore	Dignity is an important concept in palliative care. Yet, the concept is ambiguous and there does not seem to be agreement on how care can be delivered that preserves dignity at the end of life. The aim of the study was to conduct a feasibility study to explore the experience of dignity in palliative care in Singapore.	We conducted qualitative interviews with four patients with advanced cancer and their primary family caregiver from a local largest home hospice service.	he preliminary findings showed that the experience of dignity in Singapore context consists of three factors, which reflected 12 themes. These are (1) social factor, which comprised of family, friends, relatives, and employers; (2) organizational factor, which comprised of the healthcare system, voluntary welfare organization, and private sector, and (3) spiritual factor,	In conclusion, the preliminary findings suggest that using systems perspective, it is feasible to conduct a study to develop an empirical model on dignified palliative care in the Singapore context.	-	20

						which was associated with existential values, cultural and religious belief systems of the patients, and their families. The preliminary findings suggest similarity to the Western findings: dignity can be preserved by holistic care that focuses on psychosocial, physical, and spiritual aspects. In addition, dignified patient care should encompass the knowledge and sensitivity to the multi-cultural and multi-ethnic practices and health beliefs of the patients and their families.			
87. Ann Marie Dose et al.	2017	Pilot descriptive study	Feasibility and Acceptability of a Dignity Therapy/Life Plan Intervention for Patients With Advanced Cancer	To determine the feasibility and acceptability of a dignity therapy/ life plan intervention in the outpatient oncology setting.	Research Approach: Pilot descriptive study. Setting: Outpatient clinic in a tertiary oncology center. Participants: 18 patients within 12 months after diagnosis undergoing treatment for advanced pancreatic	Among the 18 patients completing the intervention, almost all felt it was worth-while, would do it again, had their expectations met or exceeded, would recommend it to others, and said the timing was just right.	Nurses may be in an ideal position to offer a dignity therapy/life plan intervention to patients with advanced cancer during treatment.	-	20

					cancer or non-small cell lung cancer. Methodologic Approach: Patients received dignity therapy, consisting of a focused life review/values clarification interview session and two subsequent sessions to produce a generativity document, which they can use later as they wish. Participants also wrote a life plan, in which they listed future hopes and dreams. Intervention feasibility and accept- ability for patients and oncology clinician satisfaction were assessed.	This psychosocial intervention was found to be feasible and acceptable to patients with cancer undergoing active treatment.			
88. Steven D. Passik et al.	2004	Feasibility Study	A feasibility study of Dignity Psychotherapy delivered via telemedicine	Dignity Psychotherapy has shown great promise as a value-affirming intervention for patients with advanced disease. We delivered the Dignity Psychotherapy intervention in a	Once eligible patients were consented on this IRB-approved study, they completed baseline assessments and were scheduled to have the videophone	Participants had a mean age of 56.32 years (range = 41-66, SD = 7.65) and were diagnosed with lung (n = 5, 62.5%), breast (n = 2, 25%), or colon cancer (n = 1, 12.5%). They reported	Telemedicine can greatly extend the benefits of Dignity Psychotherapy by bringing it to patients who are dying at home. Our very preliminary work suggests that	-	17

				feasibility study of a series of eight cancer patients via videophone technology to deliver the therapy into their homes.	placed in their homes. The Dignity Therapy sessions then encompassed a first session, which was transcribed and edited, followed by a second session to go over the edited transcript and allow the patient to make changes. Patients then filled out follow-up questionnaires and had the telemedicine equipment removed from their homes, and their legacy document delivered.	overall benefit from the intervention along with a high level of satisfaction. We were able to deliver the intervention in a timely fashion, with minimal length between sessions and transcript delivery and few technical difficulties.	delivering the intervention to patients who are too ill to leave their homes or who are in rural locations may be a feasible way to help them.		
89. Sue Hall et al.	2011	Randomized Controlled Trial	Feasibility, acceptability and potential effectiveness of Dignity Therapy for older people in care homes: A phase II randomized controlled trial of a brief palliative care psychotherapy	A pilot study of Dignity Therapy conducted with hospice patients reported high levels of self-reported benefits of the psychotherapy. This study aims To assess the feasibility, acceptability and potential effectiveness of Dignity Therapy to reduce distress in older people in care homes.	A randomized controlled open-label trial (ISRCTN37589515). Setting and participants: 60 residents aged 65 with no major cognitive impairment living in care homes in London, UK. Potential effectiveness (dignity-related distress, depression, hopefulness, quality of life at baseline and 1	We randomized 60/755 (8%) residents: 29 – control, 31 – intervention. We found no significant differences between groups on measures of potential effectiveness at any time. There was a reduction in dignity-related distress across both groups (p 1⁄4 0.026). The intervention group	Although Dignity Therapy took longer to deliver than expected, this intervention may be a way of enhancing the end-of-life experiences of residents.	15	-

					and 8-week follow-up); acceptability (residents' views on self-reported benefits of Dignity Therapy/the Dignity Therapy study); and feasibility (time taken to deliver the intervention).	outperformed the control group on all the acceptability items at both follow-ups. Effect sizes (Cohen's d) ranged from small (0.25) to large (0.85). These were significant for feeling that Dignity Therapy/the Dignity Therapy study had made their life more meaningful at 2-week follow-up (p 1⁄4 0.04), and that it would help their families at both follow-ups (p 1⁄4 0.02 and p 1⁄4 0.01, respectively). Although it was feasible to deliver the intervention in this setting, it took longer to complete than anticipated.			
90. Kei Hirai et al.	2006	Cross-Sectional Study	Good Death in Japanese Cancer Care: A Qualitative Study	One of the most important goals of palliative care is achieving a ''good death'' or a ''good dying process.'' The primary aim of this study was to identify the components of a Japanese ''good death'' through	Semistructured interviews were conducted. Thirteen advanced cancer patients, 10 family members of such patients, 20 physicians, and 20 nurses were recruited	Content analysis was applied to answers, and 58 attributes were extracted and classified into 17 categories as follows: Freedom from pain or physical/psychol	This study identified important components of a good death in Japan. A future quantitative survey is planned to clarify the generalizability of these findings	-	23

				qualitative interviews with cancer patients, their families, physicians, and nurses.	from five regional cancer institutions in Japan.	ogical symptoms, Having a good family relationship, Dying in one's favorite place/environment, Having a good relationship with medical staff, Not being a burden to others, Maintaining dignity, Completion of life, Maintaining a sense of control, Fighting against cancer, Maintaining hope, Not prolonging life, Contributing to others, Control of future, Not being aware of death, Appreciating others, Maintaining pride, and Having faith. The most frequently cited category was ''Freedom from pain or physical/psychological symptoms'' and the least common was ''Having faith.''	as the primary endpoint of palliative care in Japan.		

91. Rayan Alsuwaigh et al	2015	Mixed Methods study	How do English-speaking Cancer Patients Conceptualise Personhood?	Understanding personhood or “what makes you, you” is pivotal to the provision of person-centred care. Yet the manner that personhood is conceived amongst patients varies significantly. This study aims to investigate conceptions of personhood in a multiracial, multicultural, multireligious setting.	A mixed-methods study was conducted at National Cancer Centre Singapore, from January 2013 to April 2013. We used a validated questionnaire where English-speaking oncology patients rated the importance of 26 features of “personhood” on a 10-point Likert scale from 0 to 9, with 9-points being extremely important. This was followed by a semi-structured interview. Analysis of transcripts using the Grounded Theory revealed original data that inspired novel ideas about the nature of personhood, which precipitated a further study in April 2014.	Our initial study of 100 patients revealed that personhood is conceived in a unique and novel manner. To study this, we interviewed a further 40 patients using a supplemental question to our original questionnaire. Our data affirmed our initial findings and evidenced a change in conceptions of personhood.	Our evidence supports the Ring Theory of Personhood, which suggests that personhood is defined by innate, individual, relational, societal elements. It also evidences that personhood is temporally and contextually sensitive allowing for better appreciation of the evolving goals of care that frequently occur at end- of-life. Most importantly, this study reminds healthcare professionals on the importance of “treating persons” and looking beyond familial interests in maintaining the interests and dignity of the patient.	9.5	23
92. Virginia L Bartlett et al	2018	Grey lit	An Actual Advance in Advance Directives: Moving from Patient Choices to Patient Voices in Advance Care Planning	Since the concept of the living wills emerged nearly 50 years ago, there have been practical challenges in translating the concept of an	-	-	-	-	4

				advance directive (AD) into documents that are clinically useful across various healthcare settings and among different patient populations and cultures. Especially, challenging has been the reliance in most ADs on pre-selected Bchoices^ about specific interventions which either revolve around broad themes (e.g., Bprolong life / do not prolong life^) or whether or not to utilize particular interventions (e.g., CPR, mechanical ventilation), both of which about most laypersons know little and, more importantly, lacking context, prove to be of limited meaningfulness. Moreover, whether by foundational frame, decade-long mis-understanding in medicine and bioethics, or different societal customs, these ADs present					

				decision-making responsibility for initiating, continuing, or withdrawing med- ical interventions as a patient responsibility—creating a burden for which most patients are unprepared—and hence reducing healthcare providers' responsibility to mere tech- nical application or customer service. At our institution, significant efforts have focused on embracing the unique and complementary responsibilities of patients (articulating their goals, values, and preferences) and physicians (using medical expertise to reach patient goals) for enabling appropriate plans of care. This includes re- structuring our AD form to more accurately represent patient's values as the frame within which physicians are responsible for determining appropriate care. Rather than					

				specifying interventions, the AD makes patients responsible for specifying what matters to them as well as what they value in terms of function, interaction, and level of acceptable burden, thus providing clear goals for clinicians to pursue—or when goals are not reachable by available medical interventions, to acknowledge and allow for logical shifts to what may be achieved, including, in end-of-life contexts, care focused on respect and dignity.					
93. Gwenda Albers	2011	Cohort Study	Analysis of the construct of dignity and content validity of the patient dignity inventory	Maintaining dignity, the quality of being worthy of esteem or respect, is considered as a goal of palliative care. The aim of this study was to analyse the construct of personal dignity and to assess the content validity of the Patient Dignity Inventory (PDI) in people with an advance directive in the Netherlands.	Data were collected within the framework of an advance directives cohort study. This cohort study is aiming to get a better insight into how decisions are made at the end of life with regard to advance directives in the Netherlands. One half of the cohort (n = 2404) received	The majority of the PDI items were found to be relevant for the construct to be measured, the study population, and the purpose of the study but the items were not completely comprehensive. The responses to the open-ended question indicated that communication and care-related	This study demonstrated that the PDI items were relevant for people with an advance directive in the Netherlands. The comprehensiveness of the items can be improved by including items concerning communication and care.	-	17

					an open-ended question concerning factors relevant to dignity. Content labels were assigned to issues mentioned in the responses to the open-ended question. The other half of the cohort (n = 2537) received a written questionnaire including the PDI. The relevance and comprehensiveness of the PDI items were assessed with the COSMIN checklist ('COnsensus-based Standards for the selection of health status Measurement INstruments').	aspects were also important for dignity.			
94.	David Alvargonzález	2012	Review	Alzheimer's disease and euthanasia	Employing the tenets of philosophical materialism, this paper discusses the ethical debate surrounding assisted suicide for persons suffering end-stage Alzheimer's. It first presents a classification of the dissociative	-	-	-	-	3

				situations between "human individual" and "human person". It then moves on to discuss challenges to diagnosed persons and their caregivers in relation to the cardinal virtues of Spinozistic ethics — strength of character (fortitudo), firmness (animositas) and generosity (generositas). Finally, a number of ideas attached to the debate – "right of choice", "death with dignity", "quality of life" and "compassion in dying" – are discussed in order to clarify their foundations.					
95. Silvana Bastos Cogol et al	2015	Integrative review	Anticipated directives and living will for terminal patients: an integrative review	characterizing the national and international scientific literature about the advanced directives of living will as applied to the terminally ill patient.	integrative review considering the articles published in Portal Capes, SciELO, LILACS, MEDLINE, Journal of Bioethics and Bioethikos, with the descriptors: Advanced directives, Wills regarding life and Advance	three categories emerged: Students and professionals facing the advance directives of living will: Perceptions, opinions and practices; Patient's receptivity to the Advance Directives of Living Will; The family facing the	the relevance of the topic became evident as a guarantee of respect for the dignity and autonomy of the patient, as well as to reduce ethical conflicts faced by families and health professionals facing care at the end of life.	-	4

					Directives, Living Will and Terminally Ill, totaling 44 articles submitted to content analysis.	advance directives of living will.			
96. Marjorie C. Dobratz	2012	Cross-Sectional	“All my saints are within me”: Expressions of end-of-life spirituality	With spirituality being one of the most important components of end-of-life (EOL) care, this study explored the oral responses of 44 dying persons who expressed spirituality.	Four identified spiritual themes: religious systems of beliefs and values, life meaning, purpose and connections with others, nonreligious systems of beliefs and values, and metaphysical or transcendental phenomena served as a framework for a content analysis of 91 spiritual references.	From the content analysis, eight interrelated and separate themes emerged. Although the highest number of responses centered on religious beliefs and values, nonreligious beliefs and values that included reason, dignity, mental discipline, and communion were expressed. The themes of life meaning, purpose, and connections with others also surfaced as important aspects of EOL spirituality.	The findings support the need for hospice/palliative care professionals to approach spirituality from other than a Judeo-Christian viewpoint, help dying persons create meaning and purpose within the context of their lives, and assist them in their desire for connectedness to faith communities and other significant individuals in their lives.	-	17

Author	Year Published	Study Design	Title	Intro	Methods	Results	Conclusion	Quality Assessment	
								MERSQI Score	COREQ Score
97. Sotiria Kostopoulou et al.	2018	Cross-sectional Study	Advanced Cancer Patients' Perceptions of Dignity: The Impact of Psychologically Distressing Symptoms and Preparatory Grief	The present study assesses the relationship between patient dignity in advanced cancer and the following variables: psychological distress, preparatory grief, and sociodemographic and clinical characteristics.	The sample consisted of 120 patients with advanced cancer. The self-administered questionnaires were as follows: the Preparatory Grief in Advanced Cancer Patients (PGAC), the Patient Dignity Inventory–Greek (PDI-Gr), the Greek Schedule for Attitudes toward Hastened Death (G-SAHD), and the Greek version of the Hospital Anxiety and Depression Scale (G-HADS)	Moderate to strong statistically significant correlations were found between the 4 subscales of PDI-Gr (psychological distress, body image and role identity, self-esteem, and social support) with G-HADS, G-SAHD, and PGAC ($P < .005$), while physical distress and dependency was moderately correlated with depression. Multifactorial analyses showed that preparatory grief, depression, and age influenced psychological distress, while preparatory grief, depression, and performance status influenced body image and role identity.	Preparatory grief, psychological distress, and physical symptoms had significant associations with perceptions of dignity among patients with advanced cancer. Clinicians should assess and attend to dignity-distressing factors in the care of patients with advanced cancer.	13	-

98. Douglas MacDonald	2000	Grey lit	Beyond “death with dignity”: A hospice vignette	Patients are allowed to have hospice care when they are terminally ill, have a probable life expectancy of six months, and are not receiving curative medical treatment. However, people rarely consider hospice care six months before they die. If they are not in active treatment—receiving chemotherapy, radiation, or surgery—they are almost always in active denial, and the last thing they want to hear about is how well hospice will take care of them when they are bed-bound, incontinent, and in severe pain.	-	-	-	-	-
99. Kunihiko Wantanabe et al	2005	Grey lit	Care for dying patients with primary malignant brain tumour (respecting dignity)	Patients with primary malignant brain tumor experience deterioration of multi-focal neurological deficits such as hemiparesis, aphasia, visual field defects,	-	-	Even if patients do not suffer from complete expressive aphasia, they often have difficulty verbalizing their thoughts and feelings. Sadly, disturbance of vocal expression	-	-

				dysphagia, and disturbance of recent memory at the advanced stage of disease. With these advancing neurological deficits, many patients will inevitably prepare for death and may experience psychological and spiritual distress.			is a common accompaniment of this pathology. Unless the pathophysiology is understood, an observer may fail to comprehend the patient's non-verbal communication. Seeking to understand these issues is a prerequisite of the preservation of dignity and provision of ethical care for such patients.		
100. Karma Doma Bhutia et al.	2019	Cross-Sectional Study	Beliefs, Preferences and Practices of End-of-Life Care among Elderly	According to the modern health care needs, it is important to explore the end-of-life beliefs, preferences and practices so that dignified death is assured which is the right of every human being. It is a way that the elderly share and communicate their wishes regarding end of life that helps the health care professionals to provide better end of life care. The objectives of the study were to assess the beliefs, preferences and	An exploratory survey approach with non-probability convenient sampling was adopted for the study. The samples in the study were 384 elderly above 60 years of age residing in the adopted villages of MCON Manipal Academy of Higher Education, Manipal.	The results of the study revealed that majority (52.1%) were females with a mean age of 67years (range 60-82 years), almost (79.2%) belonged to Hindu religion. Overall (88.8%) discussed the end of life wishes with their family and friends, (100%) wants to die at home, and majority (96.40%) are taken care of by their children. Most of them are	Understanding end of life beliefs, preferences and practices will help health care professionals take appropriate decisions in caring for the elderly in their end of life.	9	-

				practices about end of life care among the elderly as measured by self-administered structured questionnaires		not aware of living wills and never heard of durable power of attorney. Most of them (96.4%) expressed their desire to travel to places of religious pilgrimage and (85.7%) desires mantra to be chanted during death and dying process. Almost (63.5%) of the elderly wishes to get I.V pain medications during the end of life and apparently (52.3%) of the elderly do not want to plan their funeral but (70.1%) elderly expects that their spiritual rituals to be conducted during the end of life.			
101. Sigrun Vehling et al	2014	Cross-sectional study	Symptom burden, loss of dignity, and demoralization in patients with cancer: a mediation model	Demoralization is a syndrome of existential distress that occurs in a substantial minority of cancer patients and is	This cross-sectional study examined N = 112 inpatients with mixed tumor sites at early and advanced disease stages using the following	Patients reported M=4.7 (SD=6.0) dignity-related problems; 20% showed moderate to severe	By supporting the mediation hypothesis, our results indicate that loss of dignity partially explains the association between physical problems and	10.5	-

				associated with a higher number of physical problems. Loss of dignity refers to a range of specific existential concerns. This study examines whether the association between number of physical problems and demoralization is mediated by loss of dignity.	standardized self-report questionnaires: Physical problems list of the National Comprehensive Cancer Network (NCCN) Distress Thermometer (DT), Patient Dignity Inventory (PDI), Demoralization Scale (DS), Patient Health Questionnaire-9 (PHQ-9) and Illness-Specific Social Support Scale Short Version-8 (ISSS-8). The mediation hypothesis was tested by multiple regression analyses controlling for age, gender, curative versus palliative treatment phase, and social support.	demoralization. Loss of dignity significantly mediated 81% of the effect of the number of physical problems on demoralization (Sobel zs=4.4, p<.001). Testing the reverse direction, we found that demoralization mediated only 53% of the association between physical problems and loss of dignity (zs = 3.7, p < .001).	demoralization. Early recognition of dignity-related existential concerns and interventions to enhance the sense of dignity may prevent demoralization in patients with cancer. Results provide a conceptual link between existential concerns (loss of dignity) and existential distress (demoralization) as two approaches to existential suffering in patients with cancer.		
102. Marinete Esteves Franco	2019	Cross-sectional study	PERCEPTION OF DIGNITY OF PATIENTS IN PALLIATIVE CARE	to understand the perception of dignity of patients in palliative care and to identify factors that may increase or decrease the sense of dignity.	an exploratory study with a qualitative approach, carried in a Palliative Care Center in São Paulo (Brazil), with the Chochinov's Dignity Model as theoretical framework and content analysis as methodological framework. The participants of this	the analysis of the perception of dignity allowed the identification of three categories: Correct person, Autonomy/independence and Socio-political factors. The factors that increased the	the perception of dignity of patients in palliative care was influenced by health professionals and caregivers. Being a "correct person", maintaining autonomy, being cared for and respected has increased the sense of dignity. Urban violence and the lack of	-	12

					study were 20 patients in palliative care, assessed through semi-structured interviews based on three questions: “What is dignity for you?”, “What increases your dignity?”, “What decreases your dignity?” The interviews were recorded with the patients’ authorization, from September to November 2017, and transcribed for content analysis.	sense of dignity were the following: Care, Independence/ autonomy, Leisure/positiv e thinking/being with friends. And those that decreased it were the following: Behaviors/attit udes, Health status and Economic situation.	compliance with accessibility policies have reduced the sense of dignity among palliative care patients.		
103. Tatsuo Akechi	2012	Cross-sectional Study	Dignity therapy: Preliminary cross-cultural findings regarding implementation among Japanese advanced cancer patients	Dignity therapy is a novel, brief, and individualized psychotherapeut ic intervention developed in Western countries1,2 and appears to be a feasible and effective approach for addressing the existential distress experienced by advanced cancer patients	This study consisted of a hospice/palliative care inpatient setting (Study 1) and an inpatient setting in a regional cancer center and/or general hospital (Study 2). The subjects were adult advanced cancer patients whose estimated prognosis was of less than six months. In Study 1, potentially eligible subjects were consecutively enrolled for the study	Regarding the DTFQ, the following findings were obtained (percentages indicate the proportion of positive responses): usefulness for improving dignity (67%), benefits (56%), improvement of meaning of current situation (56%), improvement of purpose of life (44%), usefulness for ameliorating suffering	Although dignity therapy should not be routinely recommended to all terminally ill Japanese cancer patients, this therapy may be promising for patients who hope to leave a legacy.	8.5	-

					in hospice/palliative care units. In Study 2, trained psychiatrists sampled potentially eligible participants who were expected to benefit from dignity therapy.	(44%), helpfulness for family (78%), usefulness for sense of well-being (56%), burden to physical condition (0%), and recommendation for other patients (33%).			
104. Harvey Max Chochinov	2002	Case Report (Secondary paper)	Dignity-Conserving Care— A New Model for Palliative Care	This process of genre negotiation may help to explain the positive psychotherapeutic results of dignity therapy.	This process of genre negotiation may help to explain the positive psychotherapeutic results of dignity therapy.	This process of genre negotiation may help to explain the positive psychotherapeutic results of dignity therapy.	This process of genre negotiation may help to explain the positive psychotherapeutic results of dignity therapy.	-	-
105. Susan E McClement	2004	Meta-Analysis (Secondary paper)	Dignity-conserving care: application of research findings to practice	A central tenet of palliative care is to help people die with ‘dignity’. The widespread use of this term presupposes that this construct is well understood from the perspective of the terminally ill, and that the factors that bolster or erode dignity are known. However, the paucity of research related to these issues	Over the past 5 years, this research team, headed by Dr Chochinov, has undertaken a programme of research aimed at explicating what dignity means to those who are terminally ill, and identifying those factors that support and undermine dignity in this patient population. This article will provide a synopsis of that work, with an emphasis on the	The model suggests that patient perceptions of dignity are related to, and influenced by three major thematic areas:<br>- Illness-related concerns, i.e. those issues deriving from the illness that relate to one’s level of independence and symptom experiences	The dignity-conserving model of care provides healthcare providers with an understanding of dignity from the perspective of the terminally ill, and provides direction for a range of patient-centred therapeutic interventions to be included as part of the patient’s over- all plan of comfort care. Such interventions	-	-

				suggests otherwise.	application of research findings for practice.	- The patient's dignity-conserving repertoire, i.e. the personal approaches that individuals use to maintain their sense of dignity, and the internally held views or perspectives of their inherent qualities<br>- Social dignity inventory, i.e. factors external to the patient that influence the quality of his or her interactions with others, that may bolster or undermine the person's sense of dignity.	are tangible ways of providing humane care for patients in the end stages of their life.		
106. Andrea Bover	2018	Cross-Sectional Study	Dignity-related existential distress in end-of-life cancer patients: Prevalence, underlying factors, and associated coping strategies	Cancer patients often have to face increasing levels of existential distress (ED) during disease progression, especially when nearing death. This cross-sectional study aimed to assess the	Two hundred seven cancer inpatients with a Karnofsky Performance Status ≤50 and a life expectancy of 4 months or less have been examined with the following self-report measures: PDI-IT, Demoralization	Dignity-related existential distress was a problem/major problem for 18.8% of the patients, especially for the younger ($F (1, 205) = 3.40$; $P = 0.020$) and more	This study showed how DR-ED is a relevant problem for patients nearing death and furthermore highlighted 2 underlying factors. Finally, the research has shown that positive reframing and self-blame	7	-

				prevalence of the dignity-related existential distress (DR-ED) in a sample of end-of-life cancer patients, and to explore the “existential distress” Patient Dignity Inventory (PDI-IT) subscale internal structure and its associations with different coping strategies.	Scale (DS-IT) and Brief Coping Orientation to Problem Experienced (Brief-COPE). The existential distress PDI-IT subscale factor structure was explored through principal component analysis, and the DR-ED associations with the other considered variables were examined through X2 tests, MANOVA, and multivariate regression analysis	demoralized ($F (1, 205) = 20.36$; $P < 0.001$) individuals. Factor analysis supported 2 dimensions labelled “self-discontinuity” and “loss of personal autonomy,” accounting for 58% of the variance. Positive reframing ($\beta = -0.146$, $P < 0.05$) and self-blame ($\beta = 0.247$, $P < 0.001$) coping styles emerged as DR-ED significant predictors. Conclusions: This study showed how DR-ED is a relevant problem for patients nearing death and furthermore highlighted 2 underlying factors. Finally, the research has shown that positive reframing and self-blame	coping styles might be clinically relevant elements for interventions on ED.		

						coping styles might be clinically relevant elements for interventions on ED.			
107. Gwenda Albers et al.	2013	Cohort Study	Does Health Status Affect Perceptions of Factors Influencing Dignity at the End of Life?	Context. More people are surviving into old age, and chronic diseases tend to become more common with age. Ill health and disability can lead to concerns about loss of personal dignity. Objectives. To investigate whether health status affects the perceptions of factors influencing personal dignity at the end of life, and the relationship between those perceptions and sociodemographic characteristics.	Methods. A subsample (n ¼ 2282) of a large advance directives cohort study was used. Three different health status groups (good, moderate, and poor) were defined based on the Euroqol-5D and a question on whether they had an illness. For each health status group, we calculated the percentage of respondents who indicated the extent to which the items of the Patient Dignity Inventory would influence their dignity as (very) large. Logistic regression analyses were used to investigate the associations between the perceptions of factors influencing personal dignity and sociodemographics.	The percentage of respondents who indicated the factors as having a (very) large influence on dignity at the end of life were not significantly different for the three health status groups, except for three physical items on symptoms, roles, and routines. Those items were significantly more influential on dignity for people with a poor health status. Gender, old age, having a partner, and having a belief or religion that is important to one's life were associated with an	Health status seems only to affect the perceptions of physical factors maintaining dignity at the end of life. This might suggest that the understanding of dignity will not substantially change as health status changes and may support starting advance care planning early	12	-

						understanding of factors influential to dignity.			
108. Zeinab Hemati et al.	2016	Systematic Review	Dying with dignity: a concept analysis	Dignity is an important component of providing care for dying patients and their families. Nevertheless, given that this concept is poorly defined, concept analysis is one of the best ways to define and clarify the concept of death with dignity with the aim to enhance its application in clinical practice, research and education.	A search of multiple nursing and social sciences databases was undertaken, including Academic Search Complete, Science Direct, ProQuest, Scopus, Medline, PubMed, EBSCO, Ovid, Noormage, Cinahl, Magiran, PsycINFO and SID.<br>After an extensive review of the literature from 1998–2014, about 14 related articles were included in the study. Based on these articles, the applications, attributes and experimental results of and references to death with dignity were recorded.	Based on this analysis, the most important attributes of this concept included respect for privacy, respect, spiritual peace and hope. The antecedents of this concept included consideration of moral values during caregiving, preservation of human dignity as a patient right and professional ethics, and belief in the dignity of self and others, consideration of culture in providing end-of-life care. The consequences of this concept included a sense of peace in the patient and their family, peaceful death and provision of patient privacy and comfort.	The concept of patient dignity has been referred to in many contexts. However, considering the dignity of dying patients commensurate with their culture is the most important component of care provided by nurses to facilitate a peaceful death.	-	-

109. Mahsa Zaki-Nejad et al.	2020	Quasi-Experimental Study	The Effect of Dignity Therapy on the Quality of Life of Patients with Cancer Receiving Palliative Care	Patients with cancer deal with physical, psychosocial, spiritual, and existential problems that impact on their quality of life. This study aimed to assess the effect of dignity therapy on the quality of life of mentioned patients.	In this quasi-experimental study, 50 patients with cancer hospitalized in a palliative care center in Tehran, Iran, in 2017-18 who fulfilled inclusion criteria were selected through convenience sampling. The European Organization for Research and Treatment of Cancer Quality of Life-C15-Palliative (EORTC-QLQ-C15-PAl) questionnaire was filled by patients before and 2 weeks after dignity therapy. Data were analyzed using descriptive statistics such as frequency, mean, and standard deviation, as well as inferential statistics, including independent t-test and Chi-square test.	Results showed that dignity-therapy led to more improvement in the quality of life of the intervention group ($t_{35,18} = 4.82$, $p = 0.001$). There was also a significant difference between the two groups in terms of the physical functioning scale ($t_{32,96} = -2.60$, $p = 0.01$) and emotional functioning ($t_{45,69} = 6.54$, $p < 0.001$). We also found that dignity-therapy led to more improvement in nausea and vomiting ($\chi^2 = 5.71$, $p = 0.02$), insomnia ($\chi^2 = 15.78$, $p < 0.001$), appetite ($\chi^2 = 5.09$, $p = 0.02$), and constipation ($\chi^2 = 12.50$, $p < 0.001$).	The application of new approaches like-dignity therapy could benefit patients with cancer in terms of reducing their distress, improving symptom severity, physical and emotional functioning, and total quality of life.	10	12
110. Annetie F. Street et al	2001	Discourse analysis/Grey lit	Constructions Of Dignity In End-of-Life Care	The meaning of dignity is commonly	This paper is derived from a discourse analysis	These concepts provided the	Dignity is socially constructed, individually	-	-

				assumed but rarely examined in palliative care. Dying With dignity often forms the basis of clinical decision making at the end of life, but is constructed differently depending upon setting and context. A dis-course analysis of patient and family case studies found that relationships and embodiment were important aspects of dignity that have been neglected In the literature, although these constructions of dignity matter to dying people and their families. An understanding of these constructions can assist clinicians in providing sensitive palliative care across a range of community and medical settings.	concerning portrayals of dignity at the end of life. The study drew upon the published texts provided by the literature, Internet sites, media reports, legislative and policy statements on euthanasia. and palliative care in a number of countries, along with the created texts of narratives and case studies from interviews with patients and family members. The interviews and literature were subjected to a qualitative content analysis to code the text into designated core concepts, chosen because of their faithfulness to the data and their relationship to the literature on dignity.	basis for a discourse analysis that identified discourses pertaining to dying with dignity.	perceived, embodied, and relational. In contemporary society, where the authority concerning the "right" way to die has become increasingly vested in the dying individual, dignity becomes paramount to their mode of dying. Health practitioners need to become more aware of the hidden dimensions of the kinds of dignified death that matter to patients and their families, particularly in environments where resource constraints and staff cuts limit the possibility of providing the five-star palliative care death.		
111. Ellen M. Robinson et al.	2006	Case Report/Case Series	Complexities in Decision Making for Persons with	Good end-of-life care requires that clinicians,	The first section presents three case	Three categories of patients with	Through an interdisciplinary process	-	-

			Disabilities Nearing End of Life	families, and ethicists be aware of biases that influence patient cases, particularly in the acute care setting where the aim is primarily cure and return to optimal functional level. Persons with disabilities may pose unique challenges; their potential for quality of life is viewed through the lens of highly functional clinicians who might have a biased view of the disabled person's quality of life. The authors aim to present three categories of disability that do not claim to be absolute but rather offer clinicians and ethicists a lens through which to reflect on bias that unconsciously may influence their approach to the patient who is seriously ill and may be nearing the end of life. The	examples that provide a proposed framework for thinking about similarities and differences in varying situations of patients with disabilities. The second section provides a template for applying theoretical approaches in ethics to such cases.	disabilities who might be approaching the end of life were described to help better illustrate differences in situations that can have bearing on end-of-life decision making. For patients who have lived all or most of their lives with disabilities, the disability may not be perceived as such. Although the patient's circumstances may evoke a sense of intense sympathy and possible feelings of hopelessness in the eyes of clinicians, this may not be the patient's perception. In such cases, it is key that clinical teams are aware of their bias and	that considers each case respectfully, options for extending life or allowing a peaceful death can be well thought out and considered by all involved. A commitment to such a process can only bring about good. The decisions will continue to be difficult, but with a commitment to a process based upon considerations outlined, persons with disabilities who may be approaching the final years of their lives will be afforded the dignity that each and every human being deserves.		

				categories include (a) a person who has lived with a disability from birth or early life, due to trauma or disease, and is now faced with a serious illness that requires that life-sustaining treatment; (b) the otherwise healthy person who acquires a disability through an acute event of disease or trauma and whose condition requires that life-sustaining treatment decisions be made; and (c) the person who has lived with a progressive chronic illness, such as lung or heart disease or amyotrophic lateral sclerosis, and may have gradually adjusted to disabilities imposed by the condition and now is faced with life-sustaining treatment decisions		consciously open themselves to the patient's sense of the clinical situation and future possibilities in terms of quality of life. Conversely, for the patients with an acute event leading to a disability, clinicians ought to be aware of their roles as advocates in persuading patients and families to allow time to integrate the disability with a new sense of self – a self that over time might be satisfying. Rushing to judgment in such cases might deny the patient the opportunity for rehabilitation and leave clinicians feeling badly as they retrospectively reflect upon the case.			

112. Catherine Schryer et al.	2012	Discourse Analysis	Creating Discursive Order at the End of Life: The Role of Genres in Palliative Care Settings	This article investigates an emerging practice in palliative care: dignity therapy. Dignity therapy is a psychotherapeutic intervention that its proponents assert has clinically significant positive impacts on dying patients. Dignity therapy consists of a physician asking a patient a set of questions about his or her life and returning to the patient with a transcript of the interview.	After describing the origins of dignity therapy, the authors use a rhetorical genre studies framework to explore what the dignity interview is doing, how it shapes patients' responses, and how patients improvise within the dignity interview's genre ecology	Based on a discourse analysis of the interview protocol and 12 dignity interview transcripts (legacy documents) gathered in two palliative care settings in Canadian hospitals, the findings suggest that these patients appear to be using the material and genre resources (especially eulogistic strategies) associated with dignity therapy to create discursive order out of their life events.	This process of genre negotiation may help to explain the positive psychotherapeutic results of dignity therapy.	-	18

Author	Year Published	Study Design	Title	Intro	Methods	Results	Conclusion	Quality Assessment	
								MERSQI score	COREQ score
113. Sue Hall et al	2012	Case Reports and Series	Exploring the impact of dignity therapy on distressed patients with advanced cancer: three case studies	Dignity therapy (DT) has been developed to help reduce distress experienced by people nearing the end of life; however,	We used a case study approach. Three patients with the highest levels of dignity-related distress who received DT were explored in depth.	These patients were experiencing a wide range of major or overwhelming physical and psychosocial problems when	The extent to which DT can help these patients and their families, either as a stand-alone therapy or as an adjunct to other	-	13

				evaluations of this novel intervention have largely involved non-distressed samples. The objective of this study was to explore in detail the impact often distressed patients with advanced cancer.	We collected quantitative and qualitative outcomes from patients in face-to-face interviews at baseline and at 1 and 4 weeks after completion of the intervention. We assessed dignity-related distress using the Patient Dignity Inventory. Patients rated the benefits of DT at completion of the intervention and at both follow-ups. We conducted qualitative interviews exploring experiences of DT with patients and with two recipients of generativity documents produced by patients.	they received the intervention, most of which would not be expected to be helped by DT. All felt that DT had helped them and had helped or would help their families; however, patients' concerns about their current situation made delivering the intervention challenging, and DT-relevant problems returned when a patients' condition deteriorated.	therapies, needs to be determined in studies focussing on distressed patients, particularly those with problems likely to be helped by the therapy.		
114. Ludmilla Sneesby	2014	Grey lit	Home is where I want to die: Kelly's journey	The definition of a 'good death' is centred on being peaceful, dignified and pain free. The preferred place of death has also been highlighted as an important concept in defining a good death (Cox, Almack, Pollack, & Seymour, 2011). Seventy percent of Australians express the desire	-	-	-	-	-

				to spend their last days at home. In reality only 16% of people die at home (Preferred Place of Death, 2008). With 10% of Australians dying in residential aged care facilities and approximately 20% in hospices, the rest die in hospitals (Parish et al., 2006). Family support and the family's care giving ability play a major role in determining whether a person is able to die at home. Other factors include the availability of medical and nursing care.					
115. Cheng-Pei Lin et al.	2019	Grey lit	2019 Taipei Declaration on Advance Care Planning: A Cultural Adaptation of End-of-Life Care Discussion	Every person has the right to attain a high-quality humane health care from birth to death. To assist a patient achieve high quality of life toward the end of life and ensure high quality of dying and death (e.g., a ''good death''), it is imperative to honor patient's rights of autonomy and respect their preferences regarding care decisions in health	-	-	-	-	-

				care circumstances. Advance care planning is an initiative to respect patient's values and ensure quality care in accordance with his or her preferences, usually followed by the completion of advance directives, serving as a significant means to preserve patient's dignity at the end of life. It is widely recognized as an indicator for high-quality palliative care and endorsed by many professional bodies (e.g., American, British, and Australian medical associations) around the world.					
116. Adrienne Beck	2018	Cross-sectional study	Abbreviated dignity therapy for adults with advanced-stage cancer and their family caregivers: Qualitative analysis of a pilot study	Dignity therapy (DT) is designed to address psychological and existential challenges that terminally ill individuals face. DT guides patients in developing a written legacy project in which they record and share important memories and messages with those they	Qualitative methods were used to analyse postintervention interviews with 11 participants and their legacy recipients as well as the created legacy projects. Direct content analysis was used to assess feedback from the interviews about benefits, barriers, and recommendations	Findings suggest that abbreviated DT effectively promotes (1) self-expression, (2) connection with loved ones, (3) sense of purpose, and (4) continuity of self. Participants observed that leading the development of their legacy projects promoted independent reflection,	Abbreviated DT reduces resource barriers to conducting traditional DT while promoting similar benefits for participants and recipients, making it a promising adaptation warranting further research. The importance that patients place on family and	-	22

				will leave behind. DT has been demonstrated to ease existential concerns for adults with advanced-stage cancer; however, lack of institutional resources limits wide implementation of DT in clinical practice. This study explores qualitative outcomes of an abbreviated, less resource-intensive version of DT among participants with advanced-stage cancer and their legacy project recipients.	regarding abbreviated DT. The legacy projects were coded for expression of core values.	autonomy, and opportunities for family interaction when reviewing and discussing the projects. Consistent with traditional DT, participants expressed “family” as the most common core value in their legacy projects. Expression of “autonomy” was also a notable finding.	autonomy should be honoured as much as possible by those caring for adults with advanced-stage cancer.		
117. Ilora Finlay	2009	Grey lit	The art of medicine: Dying and choosing	Calls to legalise euthanasia are not new. Only a handful of states around the world have been convinced enough to legislate it. What is new is the nature of the case that is being put forward. Historically, the case made for legalisation was that it was necessary so that dying people could avoid having to suffer pain and distress. 30 years	-	-	-	-	-

				ago, when palliative care was almost non-existent, prolonged symptomatic suffering was common among terminally ill patients, especially among those with malignant or degenerative illnesses.					
118. Mitsuyasu Kurosu	2009	Grey lit	Argument on removal of respirator in Japan	In 2006, some newspapers reported that a surgeon, removed respirators from some patients without their consent. This event precipitated debate on the issues of respirator removal and death with dignity in terminal care in Japan.	I discussed the ethical arguments pertaining to respirator removal in the following order: (1) selected reports on terminal care from academies, government, etc.; (2) three viewpoints of this issue; (2a) respect for the sanctity of life (SOL); (2b) respect for the quality of life (QOL) and (2c) respect for autonomy; (3) prerequisite conditions.	-	Even if a patient decides autonomously that he wishes his life to be prematurely terminated, he is not in a position to carry out this act himself. Therefore, the conduct of physicians has become an important issue.	-	-
119. Carlo Leget	2013	Grey lit	Analysing dignity: a perspective from the ethics of care	The concept of dignity is notoriously vague. In this paper it is argued that the reason for this is that there are three versions of dignity that are often confused. First we will take a short look at the history	-	-	-	-	-

				of the concept of dignity in order to demonstrate how already from Roman Antiquity two versions of dignity can be distinguished. Subsequently, the third version will be introduced and it will be argued that although the three versions of dignity hang together, they should also be clearly distinguished in order to avoid confusion. The reason for distinguishing the three versions is because all three of them are only partially effective. This will be demonstrated by taking the discus-sion about voluntary ‘dying with dignity’ as an example. Inspired by both Paul Ricoeur’s concept of ethics and the ethics of care a proposition will be done as to how the three versions of dignity may sustain each other and help achieve what neither one of the versions can do on its own.					

120. Andrea Rodríguez-Prat et al	2017	Systematic review	Assumptions and moral understanding of the wish to hasten death: a philosophical review of qualitative studies	It is not uncommon for patients with advanced disease to express a wish to hasten death (WTHD). Qualitative studies of the WTHD have found that such a wish may have different meanings, none of which can be under- stood outside of the patient's personal and sociocultural background, or which necessarily imply taking concrete steps to ending one's life. The starting point for the present study was a previous systematic review of qualitative studies of the WTHD in advanced patients.	The starting point for this study was the aforementioned systematic review and meta-ethnography on the WTHD in patients with life-threatening illness. The search strategy used in that review applied a combination of MeSH and free-text terms to the PubMed, Web of Science, CINAHL and PsycINFO data- bases. Fourteen qualitative studies were finally included, involving a total sample of 280 participants (patients, families and healthcare professionals).	Our analysis shows how a philosophical perspective can add to an understanding of the WTHD by taking into account cultural and anthropological aspects of the phenomenon.	We conclude that the knowledge gained through exploring patients' experience and moral understandings in the end-of-life context may serve as the basis for care plans and interventions that can help them experience their final days as a meaningful period of life, restoring some sense of personal dignity in those patients who feel this has been lost.	-	-
121. Jeremy Sugarman	2015	Grey lit	Toward Treatment With Respect and Dignity in the Intensive Care Unit	Despite concern that patients in the intensive care unit (ICU) may not be treated with respect and dignity, there is not conceptual clarity regarding what constitutes such treatment. In addition, measures specific to treatment with respect and dignity	Accordingly, a multidisciplinary group developed a conceptual model for treatment with respect and dignity in the ICU and used mixed methods to gather data on this issue. This effort included interviews with patients and families, focus groups with health care professionals,	-	Finally, pilot quantitative patient and family survey data were collected and analysed. Each of these approaches, which comprise the papers in this supplement to Narrative Inquiry in Bioethics, is briefly described in this article.	-	-

				in the ICU are unavailable.	and direct observations. These data were then compared and contrasted to synthesize what was learned.				
122. Merav Ben Natan et al	2010	Cross-sectional study	End-of-life needs as perceived by terminally ill older adult patients, family and staff	A comparison of inpatient end-of-life needs as perceived by terminally ill older adult patients, family, physicians and nurses, is lacking. This study aimed to compare the importance attributed to different end-of-life needs by terminally ill older adult patients in long-term care facilities, their families and care providers (physicians and nurses).	This descriptive, cross-sectional study recruited a convenience sample of 451 subjects, including 73 terminally ill older adult patients, 58 family members, 71 physicians and 249 nurses, from two Israeli geriatric centers.	This study found a high congruence between the staff and terminally ill older adults and their families regarding most needs identified as important to dying people. The five needs identified as most important by all subjects were: not suffering pain, having no difficulty breathing, maintaining dignity, having someone who listens, and receiving adequate nursing care. The results of this survey suggest that for terminally ill older adult patients and their families, physical care is crucial. In contrast, nurses attribute higher significance to spiritual needs, but this finding may be affected by the fact that the rate of religiosity among nurses was much higher than among	Identifying terminally ill older adults' end-of-life needs may enable nurses and physicians to modify and improve end-of-life care. This could result in a substantial decrease in suffering amongst nursing home terminally ill older adult patients and their families.	9	-

						all other research groups.			
123. Xiaocheng Liu et al.	2020	Cross-sectional study	Effects of meaning in life and individual characteristics on dignity in patients with advanced cancer in China: a cross-sectional study	This study was conducted to evaluate the effects of meaning in life and individual characteristics on dignity in patients with advanced cancer.	One hundred sixty-seven patients with advanced cancer participated in this study. Dignity was assessed with the Patient Dignity Inventory (PDI), meaning in life was assessed with the Meaning in Life Scale (MiLS), and performance status was defined as the Karnofsky Performance Status (KPS). Sociodemographic and clinical variables were also measured. Independent T tests and one-way ANOVA were performed for the PDI scores and sociodemographic and clinical variables. Relationships among the PDI, MiLS, and KPS scores were evaluated with bivariate analyses (Spearman rank correlation). A multiple linear regression analysis was conducted to determine the predictors the of PDI score.	Patients reported a mean of 4.2 (SD 4.9) problems affecting their sense of dignity; 21.6% reported moderate to severe loss of their sense of dignity. Multivariable regression analyses revealed that a lower MiLS score, younger age, inpatient status, and a lower KPS score predicted the loss of dignity. Stepwise regression showed that 49.8% of dignity-related distress could be explained by the MiLS score, age, inpatient status, and the KPS score.	Self-perceived dignity is significantly negatively associated with meaning in life, age, inpatient status, and performance status. The early recognition of risk factors for the loss of dignity and interventions to enhance meaning in life may prevent the loss of dignity in patients with advanced cancer.	9	-

124. Cayetano Fernández-Sola et al.	2018	Grey lit	Characterization, conservation and loss of dignity at the end-of- life in the Emergency Department. A qualitative protocol	to explore and understand the experiences of terminally-ill patients and their relatives regarding dignity during end-of-life care in the emergency department.	the protocol was approved in December 2016 and will be carried out from December 2016 - December 2020. The Gadamer's philosophical underpinnings will be used in the design and development of the study. The data collection will include participant observation techniques in the emergency department, in-depth interviews with terminally-ill patients and focus groups with their relatives. For the data analysis, the field notes and verbatim transcriptions will be read and codified using ATLAS.ti software to search for emerging themes.	-	The development of the protocol could contribute to the incorporation of the participants' experiences in the development of Plans for admission, placement, care and accompaniment of terminal patients and relatives in the ED.	-	-
125. David Rudilla et al.	2015	Cross-sectional study	Comparing counselling and dignity therapies in home care patients: A pilot study	Several studies have successfully tested psychosocial interventions in palliative care patients. Counselling is the technique most often employed. Dignity therapy	We developed a pilot randomized controlled trial at the Home Care Unit of the General University Hospital of Valencia (Spain). Some 70 patients were assigned to two therapy	The results of repeated-measures t tests showed statistically significant differences with respect to the dimensions of dignity, anxiety, spirituality, and quality of life for	Our study provided evidence for the efficacy of dignity therapy and counselling in improving the well-being of palliative home care patients, and it found better results in the	14	-

				(DT) has recently emerged as a tool that can be utilized to address patients' needs at the end of life. The aims of our study were to examine the effects of DT and counselling and to offer useful information that could be put into practice to better meet patients' needs.	groups. The measurement instruments employed included the Patient Dignity Inventory (PDI), the Hospital Anxiety and Depression Scale (HADS), the Brief Resilient Coping Scale (BRCS), the GES Questionnaire, the Duke–UNC-11 Functional Social Support Questionnaire, and two items from the EORTC Quality of Life C30 Questionnaire (EORTC–QLQ–C30).	both groups. However, depression increased in the DT group after the intervention, and there were no differences with respect to resilience. Therapy in the counselling group did not negatively affect depression, and resilience did improve. When post-intervention differences between groups were calculated, statistically significant differences in anxiety were found, with lower scores in the counselling group (t(68) 1⁄4 –2.341, p 1⁄4 0.022, d 1⁄4 0.560).	counselling therapy group with respect to depression, resilience, and anxiety.		
126. Hui-Ching Li et al.	2014	Cross-sectional study	Conceptualizations of dignity at the end of life: exploring theoretical and cultural congruence with dignity therapy	To explore the conceptualization of patients' dignity in the context of end-of-life care in Taiwan.	Nine people with terminal cancer and ten health professionals were recruited from palliative care services in 2008. In-depth interviews were audiotaped and transcribed verbatim. A hermeneutic approach was employed to analyse and interpret data.	Nine people with terminal cancer and ten health professionals were recruited from palliative care services in 2008. In-depth interviews were audiotaped and transcribed verbatim. A hermeneutic approach was employed to analyse and interpret data.	The concept of dignity is culturally bound and understood differently in the Chinese and Western context; such differences should be considered when planning and delivering care. Modifications should be made to dignity therapy to ensure it is culturally	-	24

							congruent with Taiwanese patients' beliefs.		
127. Dorte Toudal Viftrup et al.	2020	Cross-sectional study	Dignity in end-of-life care at hospice: An Action Research Study	The aim of this study was to explore and improve dignity in care through an action research study with patients and hospice staff at two different hospices in Denmark. This was done by exploring how patients and healthcare professionals expressed their understandings and needs concerning dignity and involving participants in the research process with the goal of improving dignity in care.	An action research method with reflection-of-praxis and action-in-praxis was applied. It was combined with methods of semi-structured individual interviews with twelve patients, five staff and nine focus-group interviews with staff.	Three themes emerged from the analysis of data. The themes were as follows: (1) being understood, (2) contributing and (3) holistic care. Deeper analysis indicated that staff understandings of dignity mostly focused on preserving patients' autonomy, whereas patients expressed needs for relational and spiritual aspects of dignity. Staff were mostly concerned about preserving patients' autonomy when providing dignity in care, how-ever, through the action-in-praxis they increased their awareness on their own praxis and patients' needs and understanding concerning dignity. The theoretical model on dignity presented in the study also worked as a map to guide staffs' reflections	-	-	22

						on dignity in praxis and facilitated a broader focus on supporting and caring for patients' dignity in care. We believe this study has improved dignity in care at the two hospices involved in the study.			
